# Discovery of indole analogue Tc3 as a potent pyroptosis inducer and identification of its combination strategy against hepatic carcinoma

**DOI:** 10.7150/thno.102228

**Published:** 2025-01-01

**Authors:** Xiao Hu, Xiaomei Tang, Xiaoman Tian, Xing Lv, Yuanyuan Zhang, Yingyue Pang, Weilong Deng, Yali Wang, Changliang Shan, Luqing Shang

**Affiliations:** 1 State Key Laboratory of Medicinal Chemical Biology, College of Pharmacy, Nankai University, Tianjin 300353, People's Republic of China.; 2 Asymchem Pharmaceuticals (Tianjin) Co., Ltd., Tianjin 300457, People's Republic of China.

**Keywords:** hepatic carcinoma, Tc3, pyroptosis, gasdermin E, synergy effect

## Abstract

**Rationale:** Hepatic carcinoma, one of the most malignant cancers in the world, has limited success with immunotherapy and a poor prognosis in patients. While pyroptosis is considered as a promising immunotherapy strategy for tumors, it still suffers from a lack of effective inducers.

**Methods:** We designed, synthesized and screened an indole analogue, **Tc3**, featuring a 2, 4-thiazolidinedione substituted indole scaffold. Western blotting, qPCR and immunofluorescence were employed to detect the levels of pyroptosis pathway induced by **Tc3**. RNA sequencing was used to identify the mechanisms of **Tc3** in hepatic carcinoma. To validate anti-tumor effect of **Tc3**, we used CDXs and PDXs mouse models *in vivo*. Then, the syngeneic effects of **Tc3** with cisplatin and anti-PD-1 antibody were verified via western blotting, immunofluorescence, flow cytometry and ELISA.

**Results:** Treatment with **Tc3** notably inhibited the growth of hepatic carcinoma both *in vitro* and *in vivo*. Mechanistically, **Tc3** inhibited the function of PRDX1 and up-regulated excessive ROS. Then, **Tc3** induced gasderminE-mediated pyroptosis by activating the endoplasmic reticulum stress. Tumor cells with high expression of GSDME achieved better responses to** Tc3**-therapy. **Tc3** also improved the efficacy of cisplatin against hepatic carcinoma. Additionally, superior synergistic treatment was observed when **Tc3** was combined with anti-PD-1 antibody. Notably, **Tc3** activated the tumor immune microenvironment (TIME) and enhanced CD8^+^ T cell infiltration in hepatic carcinoma.

**Conclusions:** Collectively, we identified **Tc3** as a promising and effective compound for treating hepatic carcinoma and established its synergistic therapeutic strategy as a pyroptosis inducer.

## Introduction

Hepatocellular carcinoma and intrahepatic ductal carcinoma account for over 90% of hepatic carcinoma cases, making it one of the most aggressive and malignant primary tumors globally 1, 2. Conventional therapeutic strategies, including chemotherapy, surgery, and radiotherapy, have shown limited success in treating hepatic carcinoma. Currently, systemic therapies with molecular targeted drugs, such as sorafenib or lenvatinib, are the preferred treatment options, and immune checkpoint blockers have revolutionized the treatment of solid tumors by relieving the restricted T cells 3-6. However, frequent side effects and resistance to monotherapy lead to poor clinical prognosis and patient survival in hepatic carcinoma 7, 8. Therefore, it is imperative to seek innovative and effective combinatorial treatment strategies to supplement monotherapy, such as pyroptosis inducers.

Nitrogen-containing heterocyclic moieties, such as benzimidazoles, benzothiazoles, indoles, acridines, oxadiazoles, imidazoles, and thiazoles, are common in natural products and pharmaceuticals 9. Among them, thiazole and Indole nuclei are considered fundamental scaffolds for anti-tumor drug research, as illustrated by clinically approved indole-based anticancer agents (e.g., panobinostat, alectinib, sunitinib, and nintedanib), and thiazole-containing drugs (e.g., tiazofurin, dasatinib, dabrafenib, patellamide A, and epothilone) 10, 11. In recent years, the hybridization of pharmacophoric subunits has been investigated as a new method to find potential anti-tumor agents. Therefore, we screened a library of approximately 1000 compounds featuring a thiazole-substituted indole scaffold and identified a small molecule (**T1**) with anti-tumor activity against HepG2 cells.

Pyroptosis is a caspase-dependent programmed cell death activated by stimuli, such as viruses, bacteria, toxins, and chemotherapy 12-15. Cleavage of gasdermin (GSDM) prompts the oligomerization of its N-terminal and the formation of pores in the cell membrane, which leads to excessive cell swelling and bubble-like protrusions on the cell surface, ultimately causing cell death 16-18. Executors of pyroptosis, such as caspase-1/11, can process and release interleukin-1β (IL-1β) through the pores formed by gasderminD (GSDMD), initiating an immune response and providing resistance to infections or toxins 16, 19. Previous studies have shown that certain chemotherapy drugs, like 5-FU, induce pyroptosis mediated by cleaved gasderminE (GSDME, also called deafness autosomal dominant 5, DFNA5), and the mechanism of cell death can shift from apoptosis to pyroptosis depending on the GSDME level 20-22. As a result of promoter methylation of the DFNA5 gene, the expression of GSDME in most mouse tumor cells is far lower than normal cells. Therefore, a combination therapy is realized using decitabine (DNA methyltransferase inhibitor) in conjunction with chemotherapeutics. It was reported that the co-delivery of metal thiosemicarbazone complexes and decitabin with the assistance of a nanodrug carrier liposome can achieve accurate chemotherapeutic pyroptosis of tumor cells 23, 24. Moreover, the construction of nanoparticles containing indocyanine green (a photosensitizer for photothermal therapy) and a metal thiosemicarbazone compound was developed to reverse tumor cisplatin resistance through GSDME-mediated pyroptosis 25. Resistance to immunotherapy can be attributed to heterogeneity in the tumor immune microenvironment (TIME). Hence, beyond the canonical and non-canonical pathways, the activation of GSDME is considered a promising approach to remodel the TIME and trigger durable anti-tumor immunity for cancer therapy 26. However, research on potent GSDME agonists and pyroptosis inducers for hepatic carcinoma is limited.

Herein, we designed, synthesized, and evaluated a series of indole analogues optimized from **T1**, identifying **Tc3** as the hit compound. **Tc3** directly induced GSDME-mediated pyroptosis and inhibited the growth of hepatic carcinoma *in vitro* and *in vivo*. Based on these findings, we sought to explore the therapeutic potential and combinatorial strategy of **Tc3**, evaluating its efficacy and safety in cancer therapy.

## Materials and methods

### Chemistry synthesis of compounds

The concrete chemistry synthesis procedures of all compounds are listed in the chemical supporting information.

### Reagents and biological resources

The reagents and biological resources are listed in the key resources table of supporting information.

### Cell culture

Hepatic carcinoma cell lines (HepG2, SK-Hep1 and PLC-PRF5) were gifts from Dr. Tao Sun, Nankai university college of pharmacy. HEK-293T cell line was purchased from Pricella cell company (Wuhan, China), and used for lenti-virus package. Alpha mouse liver 12 cells (AML-12 cells) were gifts from Dr. Junfang Qin, Nankai university college of medical. Cell lines (HepG2, SK-Hep1, PLC-PRF5 and HEK-293T) were cultured in Dulbecco's modified Eagle's medium (DMEM, Gibco) supplemented with 10% FBS (ExCell Bio), and maintained according to ATCC. AML-12 cells were cultured in special medium for AML-12 cells (Hycyte), and maintained according to ATCC.

### Cell proliferation assays

For cell proliferation curve analysis, the hepatic carcinoma cells (HepG2, SK-Hep1 and PLC-PRF5) were seeded in 24-well plates respectively, then treated with **Tc3** at concentration gradient and counted for four days. For colony formation, hepatic carcinoma cells were seeded in 6-well plates and treated with **Tc3** for almost 10 days, and the cell colonies were photographed for statistical analysis.

### LDH release assay

The hepatic carcinoma cells were seeded in 96-well plates and treated with indole analogues for three days. Of note, the compounds were added into DMEM medium supplemented with 1% FBS. The cellular supernatant was collected for LDH release detection by using LDH cytotoxicity assay kit (Beyotime, C0016). Statistical analysis was combined with the cell viability data tested by WST-1 cell proliferation and cytotoxicity assay kit (Beyotime, C0035).

### TUNEL Cy3 apoptosis detection

Briefly, the hepatic carcinoma cells were incubated in 12-well plate, adhered to a round glass slide and then treated with **Tc3**. The fluorescence staining was conducted by using one-step TUNEL Cy3 apoptosis detection kit (APExBIO, K1134) according to the manufacturer's instructions. The cells images were captured by a confocal microscope (Leica, TCS SP8).

### Wheat germ Agglutinin (WGA) staining

To examine the membrane integrity, the hepatic carcinoma cells were seeded in 12-well plate, adhered to a round glass slide and then treated with indole analogues reagents and the control medium. The cells were fixed by paraformaldehyde, stained by iFluor^TM^ 488-Wheat Germ Agglutinin (WGA) Conjugate (AAT Bioquest, 25530, 1:200) and captured by a confocal microscope. The cells of PC (positive control) group were permeabilized by 0.3% TritonX-100 before the 488-WGA staining.

### RNA sequencing analysis

HepG2 cells were treated with dimethyl sulfoxide (DMSO) or** Tc3** (4 μM) for 24h. And the total RNA was isolated using TRIzol (Solarbio, R1100). Next, the samples were processed and the RNA libraries were constructed by APTBIO company (Shanghai, China). Paired-end reads were aligned to a reference genome index which created using HISAT2, with read assignments to each gene quantified with feature counts. Gene expression was quantified using Transcripts Per Million (TPM). We identified Differentially Expressed Genes (DEGs) using the DESeq2 package in R, defining DEGs as those with an absolute log2 fold change greater than 1 and an adjusted p-value less than 0.05. We performed Gene Ontology (GO) and KEGG pathway enrichment analyses using the "cluster Profiler" package in R. GO terms and KEGG pathways with an adjusted P-value less than 0.05 were statistically significant. Our results were visualized using the ggplot2 and fact extra packages in R.

### Pharmacokinetic study of vein administration of Tc3

Chromatographic conditions employed an ultimate AQ-C18 column (2.1 mm × 50 mm, 5.0 μm) and its temperature was set to 40℃. The mobile phase consisted of methanol and 0.1% formic acid, with a gradient elution program: 0-0.01 min, methanol 15%, 0.01-2 min, methanol 95%, 2-3 min, methanol 15%. The flow rate was set to 0.6 mL/min, the elution time was 3 min and ESI-MS in negative mode MRM was used for quantitative analysis. Masslynx 4.1 software was used for data acquisition and instrument control.

Stock solutions of **Tc3** (1.0 mg/mL) and JBHDN (0.1 mg/mL) were prepared in methanol. A series of working solutions (5, 20, 100, 200, 800, 1000 and 2000 ng/mL) were added into blank mouse blood to prepare the standard curves. The JBHDN stock solution was diluted with 5% trichloroacetic acid-methanol to 100 ng/mL. In the same manner as the standard curve, quality control (QC) samples at 10 ng/mL, 450 ng/mL, and 1500 ng/mL were prepared. Next, Balb/c mice were administered intravenously (10 mg/kg). After 5 min, 15 min, 30 min, 1 h, 2 h, 4 h, 8 h, and 24 h, blood samples (20 μL) were collected from the tail vein of mice and mixed with internal standard JBHDN (100 ng/mL). The mixtures were then vortexed for 10 min at 4℃ and centrifuged at 5500 g for 10 min. The supernatant was mixed with 200 μL ultrapure water, and then injected into liquid chromatography-tandem mass spectrometry (LC-MS/MS) system for analysis.

### Liver microsome metabolic stability assay

The intrinsic clearance of **Tc3** in liver microsomes was determined at 1 μM along with positive control (12 μM of midazolam). The reaction media contained liver microsomes (0.5 mg/mL), 1.0 mM β-nicotinamide adenine dinucleotide phosphate (β-NADPH), 3 mM MgCl_2_ phosphate buffer. Microsomal suspensions were pre-incubated with **Tc3** for 3 min at 37℃. The reaction was initiated with the addition of β-NADPH and samples were collected at 0, 5, 15, 30 and 60 min. The negative control was excluded of β-NADPH. Midazolam was incubated similarly as a positive control substrate. At each time point, an aliquot was mixed acetonitrile. Samples were centrifuged and then supernatant was mixed with ultrapure water for LC-MS/MS analysis.

### Animals and tumor models

Balb/c nude mice (female, 6-week-old) were subcutaneously injected with 2 × 10^6^ HepG2 cells on the left and right flanks to construct cell-derived xenografts (CDXs). The patient-derived xenografts (PDXs) samples were gifts from Dr Changliang Shan of Nankai University. C57 BL/6N mice (female, 5-week-old) were subcutaneously injected with 2 × 10^6^ Hepa1-6 cells mixed with matrigel (ABW, 0827265) on the left and right flanks. The mice were inoculated with hepatic carcinoma CDXs or PDXs for at least four days, and treated (i.p.) by vehicle and **Tc3**. The body weight and tumor growth were recorded before every injection. The tumors were harvested and weighed after mice sacrificed. Tumor volume = 4π/3 × (Length/2) × (Width/2)^2^. All animal experiments were carried out in accordance with the recommendations of Requirements of the Ethical Review System of Biomedical Research Involving Human by National Health and Family Planning Commission of China and Nankai University Ethics Committee with written informed consent complying with the Declaration of Helsinki (IAEC ID: 2023-STDWLL-000249).

### Drugs synergistic effect *in vitro* and *in vivo*

In vitro assay, the IC_50_ values of **Tc3** and clinical drugs (cisplatin, oxaliplatin, gemcitabine, sorafenib, regorafenib, axitinib, lenvatinib and cabozantinib) were obtained. Then, the hepatic carcinoma cells were treated with **Tc3** and the drugs mentioned above for synergistic effect test. In vivo assay, Balb/c nude mice inoculated with hepatic carcinoma CDXs were divided into four groups randomly (n = 5) and treated with vehicle, **Tc3** (20 mg/kg), cisplatin (2 mg/kg), sorafenib (20 mg/kg) and combination of the two for 14 days. The body weight and tumor growth were recorded before every injection. The tumors were harvested and weighed after mice sacrificed. CDI = AB/(A×B). Where AB is the ratio of the two-drugs combination group to the control group and A or B is the ratio of the single group to the control group. CDI < 0.7 indicates a significantly synergistic effect, CDI < 1 indicates synergism, CDI = 1 indicates additivity, CDI > 1 indicates antagonism.

### Anti-PD-1 antibody synergistic effect *in vivo*

C57 BL/6N mice inoculated with Hepa1-6 CDXs were divided into four groups randomly (n = 6) and treated with vehicle (DMSO and IgG antibody), **Tc3** (10 mg/kg), anti-PD-1 antibody (10 mg/kg) and combination of the two for ten days. The body weight and tumor growth were recorded every two days and the tumors were harvested and weighed after mice sacrificed. The CDI value of tumor weight was calculated using formula mentioned before.

### Flow cytometry analysis

The lymphocytes were isolated from the tumors and blocked by purified CD16/32 antibody. Next, the cells were stained with fluorescent-conjugated antibodies (FITC-CD11b, APC-F4/80, PE-CD86, Alexa Flour® 700-CD206, APC-Ly6G, FITC-CD45, APC-CD8a, Pacific Blue^TM^-IFN-γ). The fluorescence intensity was measured by a FACS Calibur, and data were analyzed with FlowJo software.

### ELISA detection

Briefly, the Hepa1-6 CDXs from C57 BL/6N mice were homogenized and centrifuged via RIPA buffer (Solarbio, R0010). The supernatant was collected and diluted to a suitable concentration for cytokines detection using specific ELISA kits (Proteintech, Wuhan) according to the manufacturer's instructions.

### Statistical analysis

All experiments were repeated three times. Data are presented as means ± SD and were analyzed using appropriate statistical tests as indicated in the figure legends with Graphpad Prsim 9 software. Differences were considered statistically significant at p < 0.05.

## Results

### Synthesis and discovery of indole analogue Tc3

We found a series of indole analogues with promising anti-hepatoma activity. Initially, compound **T1** was identified for its anti-tumor activity against HepG2 cells (IC_50_ = 15.94 μM) through screening of in-house library of approximately 1000 compounds. To optimize the structure of **T1**, a series of derivatives with different substitutions at the R_1_, R_2_ and R_3_ sites were rationally designed and evaluated for their anti-hepatoma activities.

**Figure 1:**
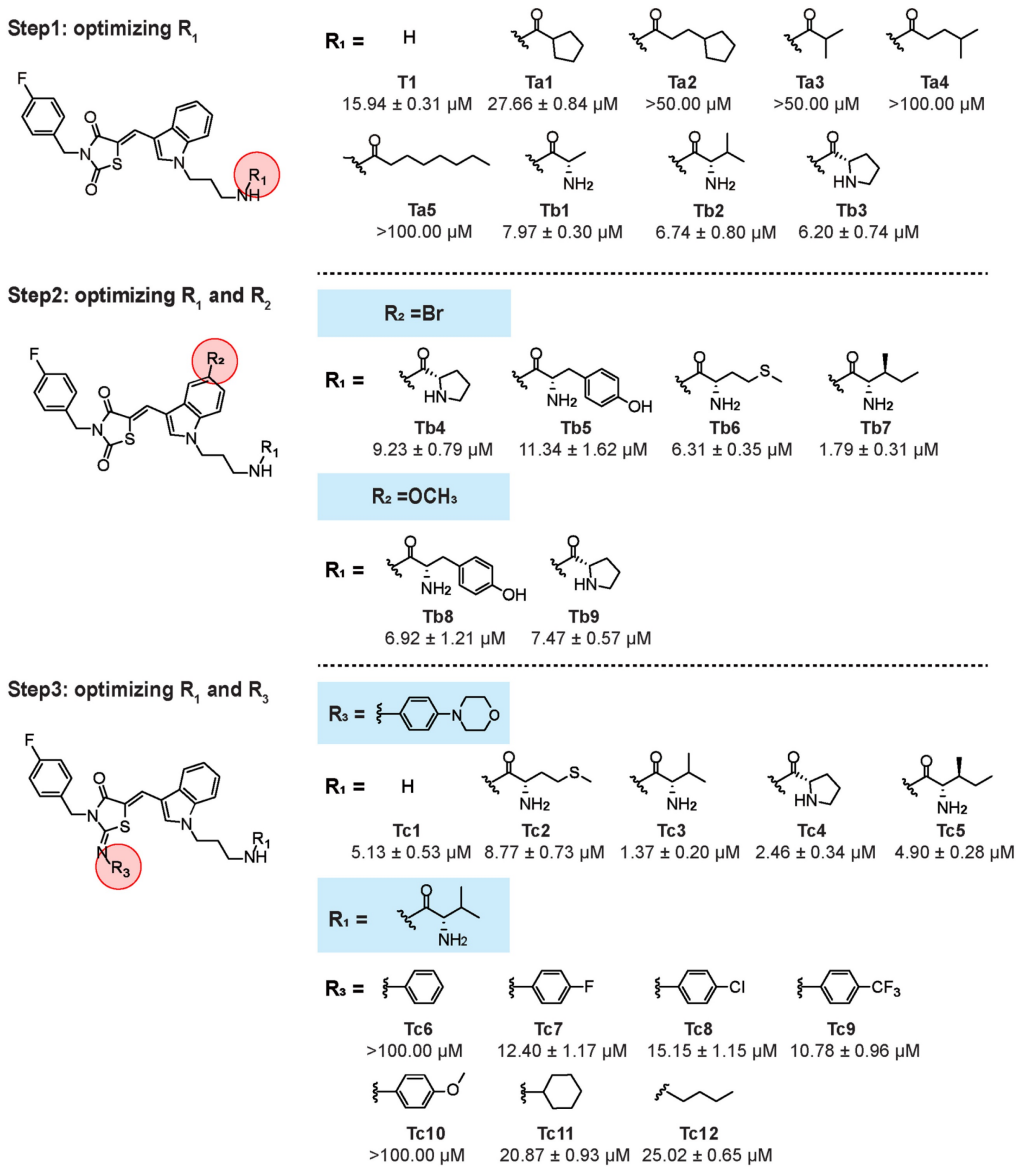
** Structure optimization process toward T1.** Step1: we introduced different carboxylic acid residues at R_1_. Step 2: we fixed R_2_ with bromine or methoxy and explored suitable amino acid moieties at R_1_. Step 3: we introduced 4-morpholinoaniline at R_3_, explored suitable amino moieties at R_1_, then fixed R_1_ and optimized R_3_.

The results were as follows: (1) A hydrophilic group at R_1_ is essential for cellular inhibitory potency. Compounds **Ta1**-**Ta5**, with fatty acid groups, showed IC_50_ values greater than 50 μM (except **Ta1** with 27.66 μM). In contrast, compounds **Tb1**-**Tb9**, with amino acid groups, had IC_50_ values below 10 μM (except **Tb5** with 11.34 μM), with **Tb7** being particularly potent (IC_50_ = 1.79 μM). (2) For R_2_, the substitution H with Br or OCH_3_ at the 5-position of the indole ring did not significantly influence anti-tumor activity, as shown by the similar IC_50_ values of **Tb3**, **Tb4** and **Tb9** (6.20, 9.23 and 7.47 μM, respectively). (3) For R_3_, introducing a 4-morpholinoaniline group led to **Tc1**, exhibiting a ∼3-fold increase in potency (IC_50_ = 5.13 μM). Further modifications on the amino group of **Tc1** resulted in **Tc2**, **Tc3**, **Tc4** and **Tc5**, showing∼2-fold, ∼12-fold,∼6.5-fold and ~3-fold increase in cellular potency, respectively (IC_50_ = 8.77, 1.37, 2.46 and 4.90 μM, respectively). Compounds **Tc6**-**Tc10**, which contain other substituted phenyl groups instead of the 4-morpholinoaniline, showed similar or worse activities, with IC_50_ values greater than 100 μM for **Tc6** and **Tc10**, and 12.40, 15.15, and 10.78 μM for **Tc7**,** Tc8**, and **Tc9** respectively. Introducing aliphatic groups at R_3_ (**Tc11** and **Tc12)** resulted in decreased potency, with IC_50_ values of 20.87 and 25.02 μM, respectively (Figure 1).

**Figure 2:**
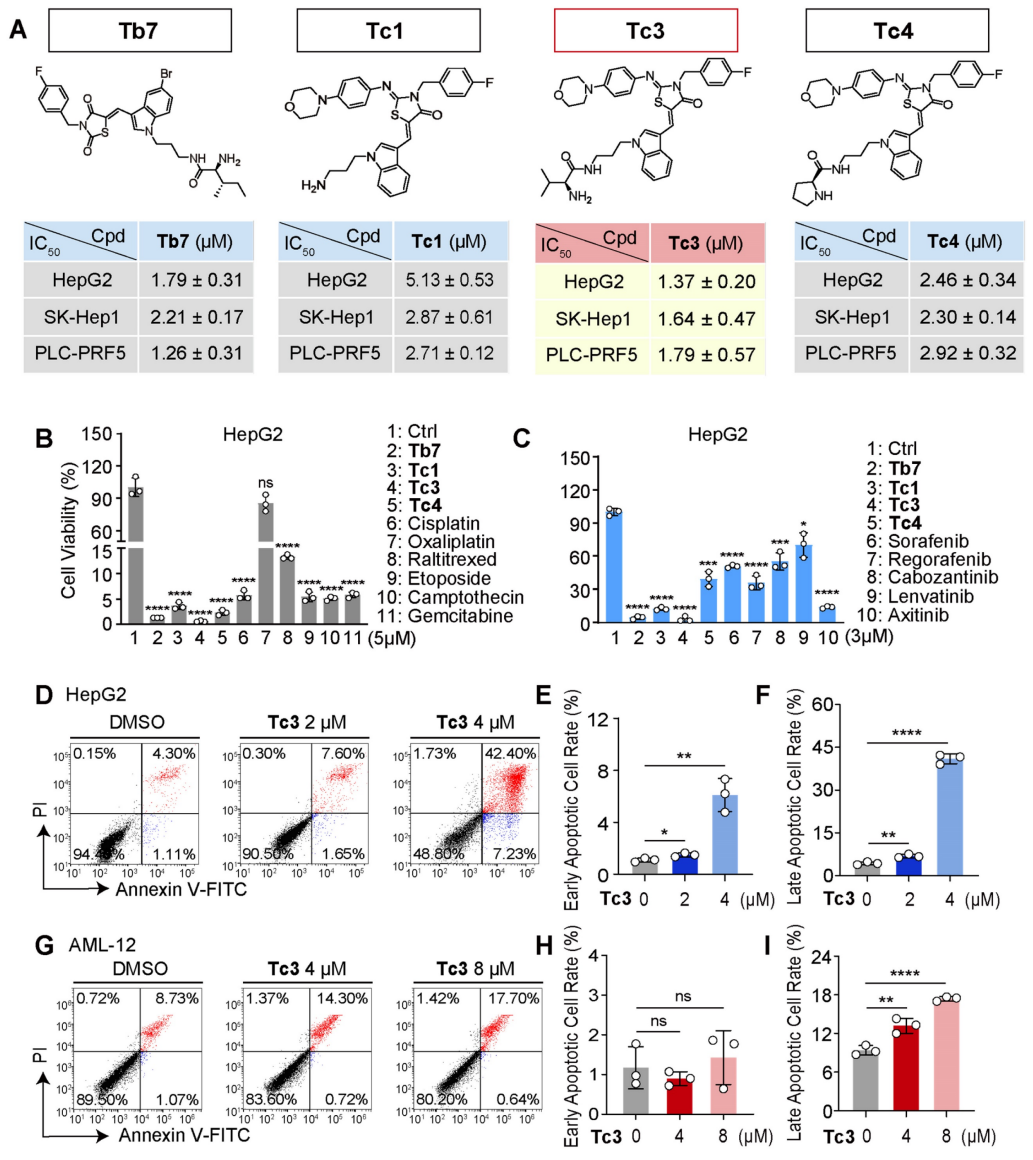
**Cytotoxic effect of Tc3 on human hepatic carcinoma cells *in vitro*.** (A) Chemical structures of **Tb7**, **Tc1**,** Tc3**,** Tc4** and the IC_50_ values after treatment of HepG2, SK-Hep1 and PLC-PRF5 cells at indicated concentrations for 72 h. (B-C) Cell viability rates of HepG2 (B) and SK-Hep1 (C) cells treated with **Tb7**, **Tc1**, **Tc3**, **Tc4** and other clinical chemotherapy drugs. (D-F) Flow cytometry of propidium iodide and annexin V-fluorescein isothiocyanate (FITC)-stained HepG2 cells (D). Statistical analysis of the early apoptotic cells (E) and late apoptotic cells (F). (G-I) Flow cytometry of propidium iodide and annexin V-fluorescein isothiocyanate (FITC)-stained AML-12 cells (G). Statistical analysis of the early apoptotic cells (H) and late apoptotic cells (I). Data are presented as mean ± SD of three independent biological experiments. *p < 0.05, **p < 0.01, ***p < 0.001 and ****p < 0.0001, ns, no significant.

We further evaluated the anti-tumor activity of **Tb7**, **Tc1**, **Tc3**, and **Tc4** against SK-Hep1 and PLC-PRF5 cells, confirming that **Tc3** had the highest potency (IC_50_ = 1.64 and 1.79 μM, respectively) for hepatic carcinoma (Figure 2A). **Tc3** also showed superior effects against hepatic carcinoma cells compared to other clinical chemotherapy drugs, including cisplatin, oxaliplatin, raltitrexed, etoposide, camptothecin, and gemcitabine at 5 μM (Figure 2B and S1A-B). Meanwhile, **Tc3** outperformed clinical targeted drugs such as sorafenib, regorafenib, cabozantinib, lenvatinib, and axitinib at 3 μM (Figure 2C and S1C-D). We also confirmed the anti-tumor activity of **Tc3** against mouse liver parenchymal cells (alpha mouse liver 12 cell, AML-12), as shown by the IC_50_ value at 6.14 μM (Figure S1E). Flow-cytometry analyses of annexin V and propidium iodide staining found that **Tc3** could induce severe cell death of HepG2 cells at 4 μM (Figure 2D-F), which was not observed in AML-12 cells (Figure 2G-I). It indicated that compound **Tc3** had selective toxicity in hepatic carcinoma cells and liver parenchymal cells. In addition, **Tc3** were found to be remarkably resistant and stable to hydrolysis since it showed no changes in a phosphate buffer of PH 7.4 over 150 minutes (Figure S1F). Our findings revealed a moderate extent of **Tc3** in mouse vitro metabolism with t_1/2_ of 116.0 min and a high extent of in human vitro metabolism of **Tc3** with t_1/2_ of 49.5 min (2.72 min and 2.90 min for midazolam, respectively). CLint was calculated to be 0.0120 and 0.0280 mL/min/mg in mouse and human respectively (1.27 and 1.20 for midazolam). A middle level of pre-systemic metabolism in liver microsomes was observed (Figure S1G-H). Therefore, we explored the mechanism of **Tc3**'s anti-tumor effect in greater depth, to highlight its potential as a useful agent for *in vivo* studies.

### Tc3 induces pyroptosis in hepatic carcinoma cells

We further verified the anti-tumor effects of **Tc3** in hepatic carcinoma cells. **Tc3** significantly inhibited tumor proliferation, as demonstrated by cell growth curve analysis (Figure S2A-C) and colony formation assays (Figure S2D-E) in HepG2, SK-Hep1, and PLC-PRF5 cells. Additionally, **Tc3** treatment reduced cell migration rates, as evidenced by wound scratch (Figure S2F-I) and transwell assays (Figure S2J-K). These results indicated that **Tc3** effectively inhibits the growth and migration of hepatic carcinoma cells, making it a promising therapeutic agent.

**Figure 3:**
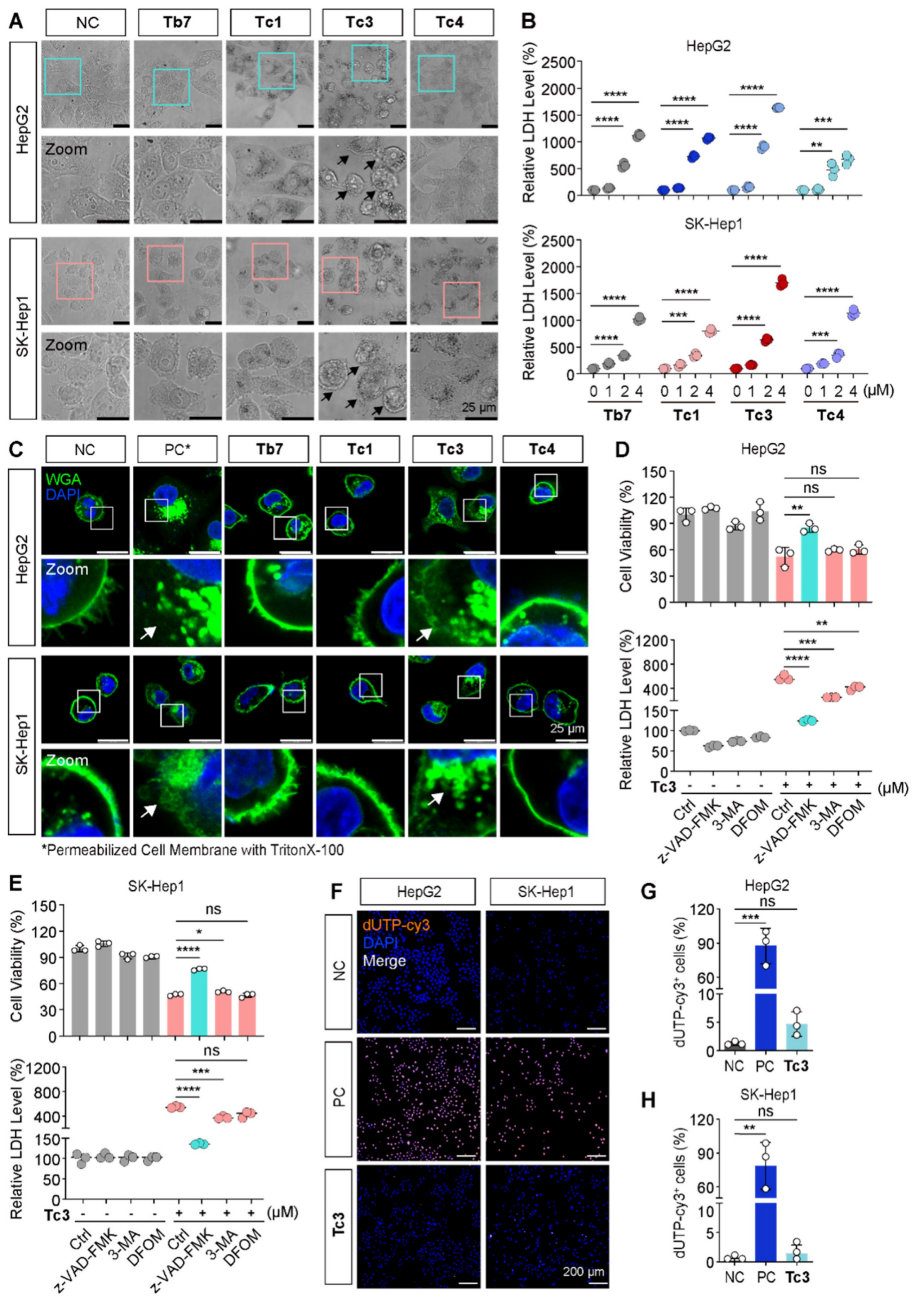
** Tc3 induces pyroptosis and impairs cell membrane *in vitro*.** (A) Representative microscopy images of HepG2 and SK-Hep1 cells after treatment with **Tb7**,** Tc1**, **Tc3**,** Tc4** and control medium. Scale bars: 25 μm. (B) LDH release assay of HepG2 and SK-Hep1 cells treated with **Tb7, Tc1**, **Tc3** and **Tc4** at a concentration gradient (0-4 μM) (C) Representative fluorescence staining images of HepG2 and SK-Hep1 cells treated with **Tb7**,** Tc1**, **Tc3**,** Tc4** and control medium. Cell membranes were labeled by 488-WGA and cell nuclei were labeled with DAPI. Scale bars: 25 μm. (D-E) Cell viability test and LDH release assay of HepG2 (D) and SK-Hep1 (E) cells treated with **Tc3** (2 μM) alone or in combination with z-VAD-FMK (3 μM), 3-MA (1 mM) and DFOM (200 nM). (F-H) Representative fluorescence staining images of HepG2 and SK-Hep1 cells treated with **Tc3**. Apoptotic cells were labeled with dUTP-cy3, and cell nuclei were labeled with DAPI (F). Statistical analysis of dUTP-cy3 positive cells in HepG2 (G) and SK-Hep1 (H) cells. Scale bars: 200 μm. NC, negative control; PC, positive control. Data are presented as mean ± SD of three independent biological experiments. *p < 0.05, **p < 0.01, ***p < 0.001 and ****p < 0.0001, ns, no significant.

To investigate the mode of cell death induced by **Tc3**, we first observed the morphology of hepatic carcinoma cells treated with various indole analogues. As shown in Figure 3A, treatment with **Tb7**,** Tc1**, **Tc3**, and **Tc4** induced the appearance of cytoplasmic vesicles to varying degrees. Consistent with the IC_50_ data, **Tc3** caused pronounced membrane blebbing and rupture, leading to the damage of the integrity of the cell membrane (Figure 3C). Moreover, the LDH release levels were found all increased in the culture medium of cells treated with **Tb7**,** Tc1**, **Tc3** and **Tc4**, indicating cell membrane disintegration and cell death. **Tc3** treatment resulted in the highest LDH release, suggesting pore formation in the cell membrane characteristic of pyroptosis (Figure 3B). When cells were finally disintegrated via various forms of programmed cell death such as apoptosis and necrosis, the LDH release could also occurs. These observations led us to hypothesize that **Tc3** might induce pyroptosis in hepatic carcinoma cells.

To further confirm the type of cell death induced by **Tc3**, we pre-treated hepatic carcinoma cells with z-VAD-FMK (a pan-caspase inhibitor), 3-Methyladenine (3-MA, an autophagy inhibitor), and Deferoxamine mesylate (DFOM, a ferroptosis inhibitor) before **Tc3** treatment. Only z-VAD-FMK was able to rescue the cells from **Tc3**-induced death and reduce LDH release (Figure 3D-E). However, the RIPK1 inhibitor necrostatin-1 could also slightly rescue the cells death and the LDH release induced from **Tc3** (Figure S2L-M). As inflammatory related cell death, pyroptosis and necroptosis both caused cell membrane rupture. In view of the activation of caspase8, it might prevent the initiate of necroptosis. TUNEL-Cy3 staining further confirmed that **Tc3** induced caspase-mediated cell death, but not apoptosis (Figure 3F-H). As shown in Figure 2D, the most hepatic carcinoma cells treated with **Tc3** were directly into late apoptotic stage. These data collectively demonstrate that **Tc3** induces pyroptosis in hepatic carcinoma cells.

### Tc3 activates GSDME-mediated pyroptosis of hepatic carcinoma cells

**Figure 4:**
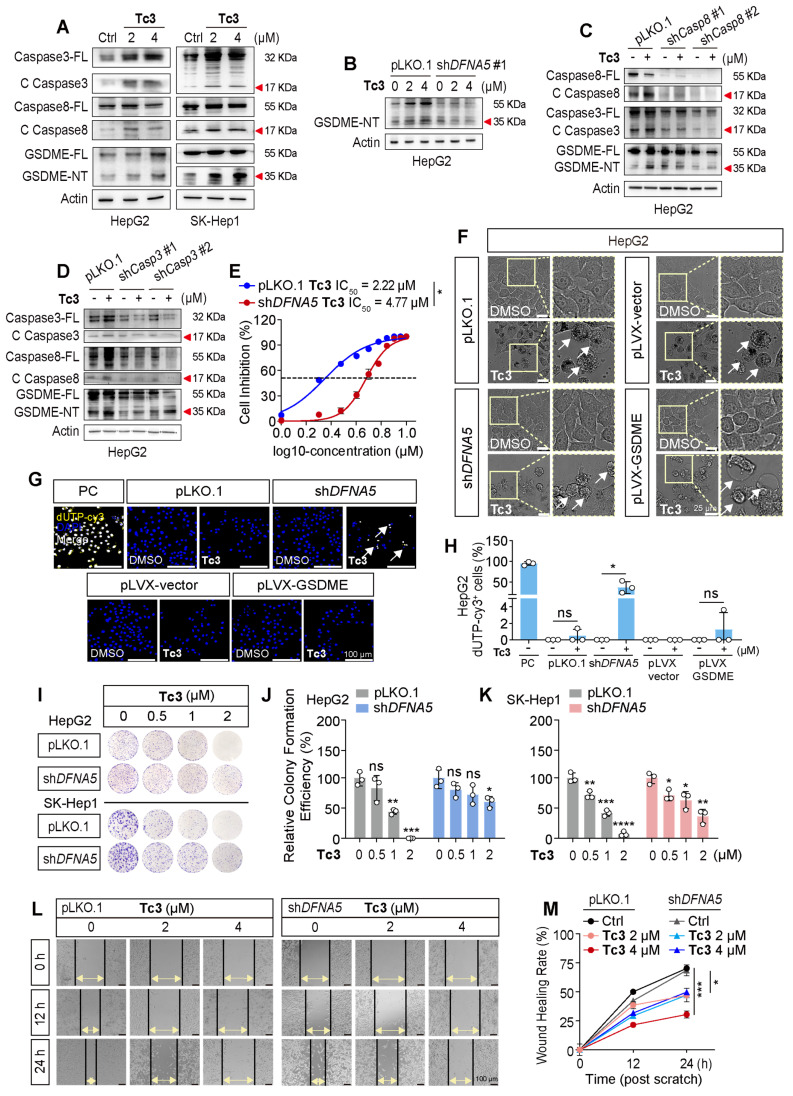
** Tc3 activates the caspase8-caspase3-GSDME axis in hepatic carcinoma cells.** (A) Immunoblot analysis showing activation of caspase3, caspase8 and GSDME in HepG2 and SK-Hep1 cells treated with **Tc3**. (B-D) Levels of GSDME, caspase8 and caspase3 in pLKO.1, sh*DFNA5* (B)*,* sh*Caspase8* (C) and sh*Caspase3* (D) HepG2 cells treated with **Tc3**. (E) Dose-response curves for **Tc3** inhibiting the growth of pLKO.1 and sh*DFNA5* HepG2 cells. (F) Representative microscopy images of pLKO.1, sh*DFNA5*, pLVX-vector and pLVX-GSDME HepG2 cells treated with **Tc3** and control medium. Scale bars: 25 μm. (G-H) Representative fluorescence staining images of pLKO.1, sh*DFNA5*, pLVX-vector and pLVX-GSDME HepG2 cells after treatment with **Tc3** (G). Apoptotic cells were labeled with dUTP-cy3, and cell nuclei were labeled with DAPI. Scale bars: 100 μm. Statistical analysis of dUTP-cy3 positive HepG2 cells (H). PC, positive control. (I-K) Colony formation assay of pLKO.1 and sh*DFNA5* of HepG2 and SK-Hep1 cells treated with **Tc3** at various concentrations (I). Statistical analysis of colony counts for HepG2 (J) and SK-Hep1 (K) cells. (L-M) Wound scratch assay of pLKO.1 and sh*DFNA5* SK-Hep1 cells treated with **Tc3** at various concentrations (L). Scale bars: 100 μm. Statistical analysis of the wound healing rate (M). Data are presented as mean ± SD of three independent biological experiments. *p < 0.05, **p < 0.01, ***p < 0.001 and ****p < 0.0001, ns, no significant.

Members of the caspase and gasdermin families are crucial initiators and executors of pyroptosis. Currently, caspase1, caspase4/5, caspase3, and caspase8 have been identified as being related to pyroptosis, which can be activated by stimulants such as infections or toxins in humans 19. We knocked down these pyroptosis-related caspases and gasdermins, and treated the stable knockdown cell lines with **Tc3** (Figure S3). Our results demonstrated that the reduction of caspase3 and caspase8 partially rescued the hepatic carcinoma cell death rate and LDH release induced by **Tc3**. Correspondingly, the knockdown of GSDME (*DFNA5*) reduced the anti-tumor activity of **Tc3** (Figure S4A-D and S5A-B). Western blotting results confirmed that the indole analogue **Tc3** activated caspase3/8 and promoted the cleavage of GSDME, but not GSDMB or GSDMD. As a primary responder of **Tc3**, GSDME was relatively highly expressed in hepatic carcinoma cells, and the full-length GSDME could be up-regulated by **Tc3**, ensuring a better effect of **Tc3** on hepatocarcinoma (Figure 4A and S4E). Other analogues, such as** Tb7**,** Tc1**, and **Tc4**, did not activate GSDME as expected (Figure S4F).

Of note, gasdermins were highest expressed in SK-Hep1 cells (liver sinusoidal endothelial immortal cells, LESCs). Therefore, SK-Hep1 cells were chosen as a representative GSDME over-expressed cell line for further study (Figure S5C). Additionally, GSDMA, GSDMC and PJVK were lowly expressed in hepatic carcinoma cells, especially GSDMA. Furthermore, the knockdown of caspase8 also restrained the activation of caspase3 and GSDME induced by **Tc3**, similarly to the reduction of caspase3 or GSDME, which further disturbed pyroptosis of cancer cells and LDH release (Figure 4B-D). As shown in Figure 4E, the IC_50_ value of **Tc3** was increased from 2.22 μM to 4.77 μM after reducing the level of GSDME. We summarized that **Tc3** activated the caspase8-caspase3-GSDME pathway and induced pyroptosis in cancer cells.

Previous studies have found that GSDME can convert caspase3-mediated apoptosis induced by TNF or chemotherapy drugs into pyroptosis, which is determined by the level of cellular GSDME. Here, we further knocked down or over-expressed GSDME in hepatic carcinoma cells to verify whether **Tc3** could induce a change in the cell death mechanism (Figure S5D). Intriguingly, after reducing GSDME, the morphology of cancer cells treated with** Tc3** showed shrinkage and became budding on cell membrane like apoptotic bodies.

Conversely, control or GSDME over-expressing cells exhibited swelling with bubble-like protrusions, and the inner cytoplasm became increased and gradually transparent when treated with **Tc3** (Figure 4F and S5E). TUNEL-Cy3 staining also showed that **Tc3** could promote apoptosis in a proportion of cancer cells when GSDME was knocked down (Figure 4G-H and S5F-G). The cell colony formation assay and cell scratch closure test demonstrated that **Tc3** inhibited the proliferation (Figure 4I-K) and migration of hepatic carcinoma cells via activating GSDME (Figure 4L-M). The up-regulation of GSDME induced by **Tc3** explains why the anti-tumor activity of **Tc3** was rescued after the knockdown of GSDME, even though the cell death mechanism shifted from pyroptosis to apoptosis. These results indicate that the treatment of tumor types with high GSDME such as hepatic carcinoma was suitable for using **Tc3**, thereby inducing potent GSDME-mediated pyroptosis.

### Tc3 triggers ER stress via PERK-EIF2α pathway for the induction of pyroptosis

**Figure 5:**
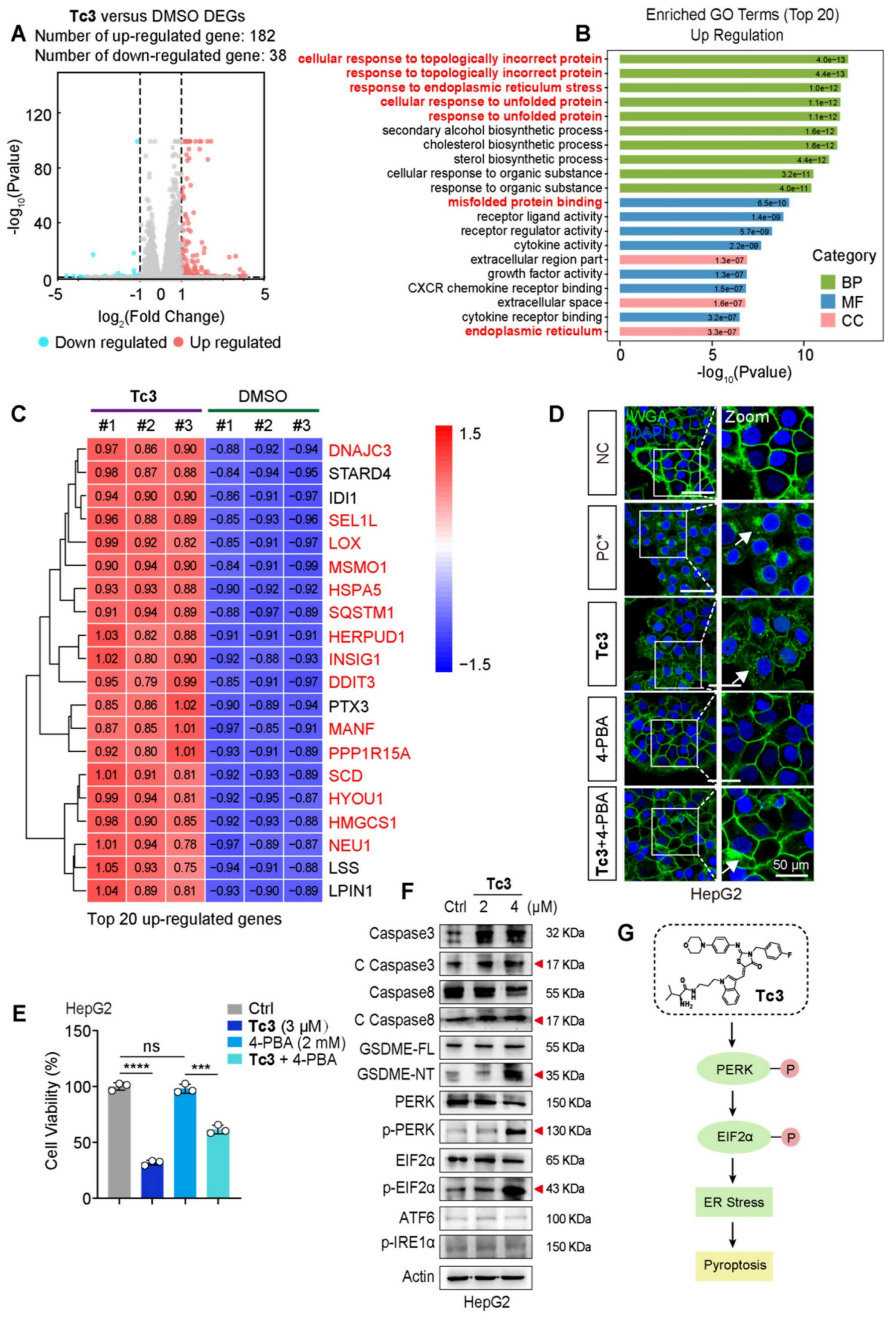
** Tc3 induces ER stress via the PERK-EIF2α pathway in hepatic carcinoma cells.** (A) HepG2 cells were treated with **Tc3** for RNA sequencing and a volcano plot of DEGs were generated (up-regulated genes shown in red; down-regulated genes shown in blue; non-regulated genes shown in gray) with|log2FC| ≥ 1 and q-value ≤ 0.05. (B) GO enrichment analysis identified “up-regulation of cellular response to topologically incorrect protein and regulation of response to endoplasmic reticulum stress” as a strongly associated biological processes in the** Tc3** group. (C) Heatmap depicting the top 20 most significantly over-expressed genes in the **Tc3** group compared to the control group. The color key corresponds to the row Z score (DMSO group shown in blue; **Tc3**-treated group shown in red). (D) Representative fluorescence staining images of HepG2 cells treated with **Tc3** (4 μM) and 4-PBA (2 mM). Cell membranes were labeled with 488-WGA and cell nuclei were labeled with DAPI. PC, positive control, and cell membranes were permeabilized with 0.1% TritonX-100 in PC group. Scale bars: 50 μm. (E) Cell viability rate of HepG2 cells after treatment of **Tc3** combined with 4-PBA. (F) Immunoblot analysis of the expression of caspase3, caspase8, GSDME, PERK, p-PERK, EIF2α, p-EIF2α, ATF6 and p-IRE1α in HepG2 cells treated with **Tc3**. (G) Schematic representation of the PERK-EIF2α pathway induced by **Tc3**. Data are presented as mean ± SD of three independent biological experiments. ***p < 0.001 and ****p < 0.0001, ns, no significant.

To elaborate on the mechanism of pyroptosis induced by** Tc3**, we performed RNA sequencing analysis using total RNA samples from HepG2 cells. The sequence data identified 220 DEGs, with 182 genes up-regulated, and 38 genes down-regulated (Figure 5A). We primarily focused on analyzing the highly up-regulated genes in subsequent steps. Gene ontology (GO) analysis revealed “cellular response to topologically incorrect protein”, “response to endoplasmic reticulum stress” and “response to unfolded protein” as the top up-regulated biological processes upon **Tc3** treatment. This indicated endoplasmic reticulum dysfunction and activation of the unfolded protein response (UPR) (Figure 5B). Moreover, Kyoto Encyclopedia of Genes and Genomes (KEGG) enrichment analysis also demonstrated that the up-regulation of protein processing in the endoplasmic reticulum (Figure S6A). Consistently, hierarchical clustering of the top 20 most highly up-regulated genes following **Tc3** treatment revealed that the prominence of endoplasmic reticulum stress (ER stress) related genes, such as *HSPA5*, *DNAJC3*, *LOX*, *SQSTM1*, *HERPUD1*, *INSIG1*, *SCD*,* HYOU1,* and *HMGCS1* (Figure 5C). We next detected the mRNA levels of these representative genes in hepatoma cells treated with **Tc3** via qRT-PCR, and confirmed the up-regulation of these ER stress related genes induced by **Tc3** (Figure S6B).

Previous studies have shown that excessive ER stress initiated by UPR can trigger cell death and inflammatory pathways 27. Based on our RNA sequencing analysis, we hypothesized that **Tc3**-indcued GSDME-mediated pyroptosis might be modulated by the ER stress pathway. To verify this, hepatoma cells were treated with 4-PBA (an ER stress inhibitor) followed by **Tc3** at an applicable concentration. The inhibitor 4-PBA could rescue cell viability and alleviate cell membrane rupture caused by **Tc3** in hepatic carcinoma cells (Figure 5D-E and S6C). The responders of UPR and ER stress, including IRE1α, PERK, and ATF6, are typically activated to initiate downstream signaling pathways to reduce ER stress 28, 29. Western blotting demonstrated that **Tc3** activated protein kinase R-like endoplasmic reticulum kinase (PERK) but not other ER stress responders, and it also facilitated the phosphorylation of EIF2α (Figure 5F and S6D). These results indicate that **Tc3** activates PERK-mediated ER stress, which subsequently leads to the activation of caspase3 and GSDME-mediated pyroptosis in hepatic carcinoma cells (Figure 5G).

Previous research also found that excessive accumulation of reactive oxygen species (ROS) could lead to oxidative stress inside cells, which further caused the organelle damage such as mitochondria or endoplasmic reticulum 28, 30. The DCFH-DA probes of ROS indicated that the inside ROS were observably up-regulated by **Tc3** (Figure S7A). It is notably that the accumulation of ROS is likely due to the suppression with its clearing system. Ruled out the ROS clearance system associated with ferroptosis like GPX4, we attempted to focus on an important member of the peroxidase reductase family, PRDX1, which had peroxidase activity and could remove peroxides such as hydrogen peroxide, organic hydrogen and peroxynitrite in cells 31, 32. The expression of PRDX1 was obviously reduced in hepatic carcinoma cells treated with** Tc3** (Figure S7B). We also proved that 4-PBA could disturb the activation of PERK-EIF2α-ATF4 pathway and the ER stress induced by **Tc3**, but not reverse the inhibition of **Tc3** to PRDX1 (Figure S7C). The fluorescence staining was conducted to demonstrate **Tc3** activated PERK by weakening PRDX1 function, resulting in impaired endoplasmic reticulum (Figure S7D-F). In addition, the knockdown of PRDX1 partially rescued the hepatic carcinoma cell death rate and LDH release induced by **Tc3** (Figure S7G). As shown in Figure S7H, the reduction of PRDX1 directly restrained the activation of caspase3, PERK and EIF2α which induced by **Tc3**. Notably, the PRDX1 knockdown cells also impeded the up-regulation of ROS induced by **Tc3** (Figure S7I-J). Collectively, the indole derivative **Tc3** activated PERK-mediated ER stress via inhibiting the function of PRDX1 and up-regulating the inside ROS.

### Tc3 inhibits the growth of hepatic carcinoma *in vivo*

We next aimed to characterize the pharmacological properties of **Tc3** *in vivo*. Pharmacokinetic profiling of **Tc3** indicated effective venous absorption, characterized by a relatively long half-life elimination (t_1/2_ = 8.08 h). The plasma drug concentration of **Tc3** ranged from 12.4-1417.0 ng/mL, and values of AUC_0-t_ was 1524 h*ng/mL (Figure S8A). To carefully evaluate the safety of **Tc3**, we conducted a subchronic toxicity test in Balb/c mice (Figure S8B). Mice were treated with **Tc3** at a concentration gradient (20, 40 and 60 mg/kg) by intraperitoneal injection (i.p.) every two days and were sacrificed on day 14. The nude mice all survived during the subchronic toxicity test. However, mice treated with the high dose (60 mg/kg) of **Tc3** exhibited lower weight and worse health state (Figure S8C-D). As shown in Figure S8E, we investigated the toxicity of **Tc3** via analyzing organ sections stained with hematoxylin-eosin (HE). **Tc3** showed no significant toxicity to the heart, liver, spleen and stomach. However, **Tc3** delivery in mice caused alveolar wall thickening and increased numbers of lymphocytes. In addition, the glomerular structure of the kidney was slightly damaged in mice treated with 60 mg/kg of **Tc3**. It is noteworthy that the immune cell infiltration of the jejunum increased and the crypts and villi were severely disrupted in mice injected with 60 mg/kg of **Tc3**. Thus, it is crucial to treat hepatocarcinoma xenografts with an appropriate dose of **Tc3** to avoid bodily damage to the mice.

**Figure 6:**
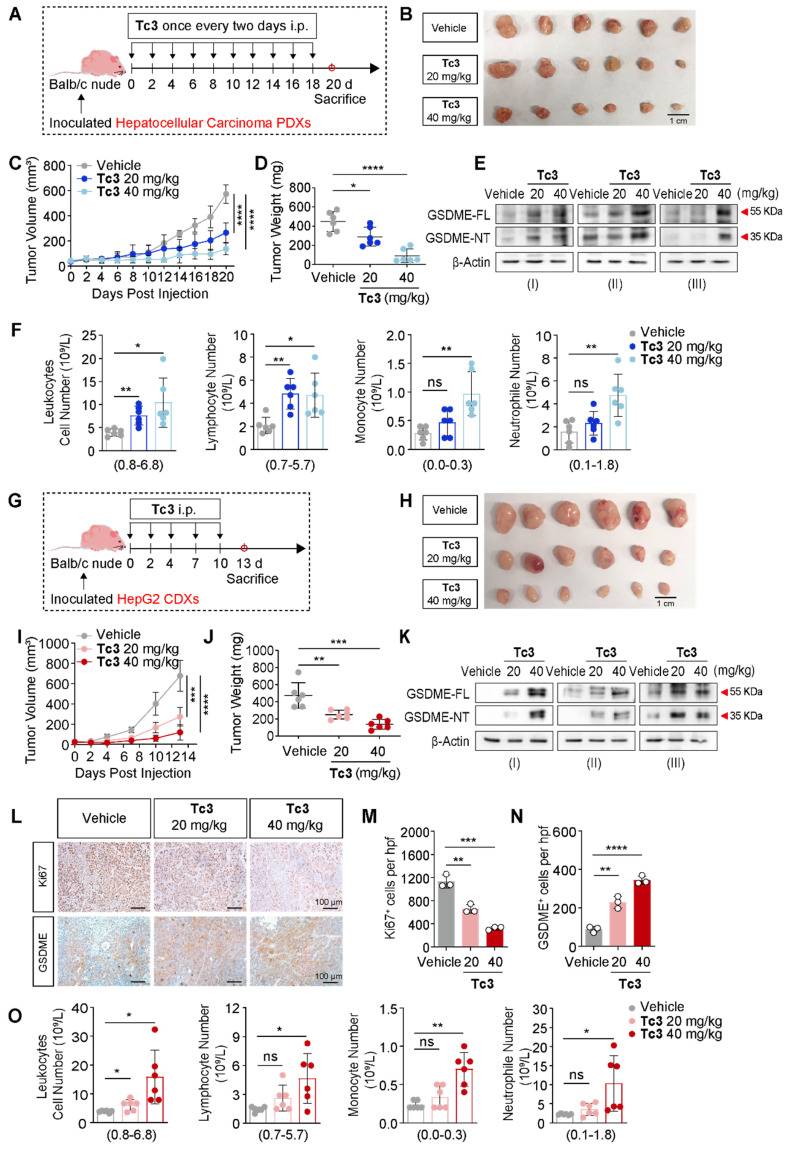
** Tc3 inhibits the growth of hepatic carcinoma PDXs and CDXs* in vivo*.** (A) Schematic depicting the process of **Tc3** treatment on tumors derived from hepatocarcinoma patients in Balb/c nude mice (n = 6). (B-D) Images of PDXs (B), tumor volumes (C) and tumor weight (D) from nude mice treated with **Tc3** (20 mg/kg and 40 mg/kg) and control medium (n = 6). (E) Immunoblot analysis of GSDME activation in PDXs from three nude mice treated with **Tc3** and control medium. (F) Blood routine examination of nude mice bearing hepatocarcinoma PDXs after **Tc3** treatment, including statistical analysis of leukocytes, lymphocytes, monocytes and neutrophils. (G) Schematic depicting the process of **Tc3** treatment on tumors derived from HepG2 cells in Balb/c nude mice (n = 6). (H-J) Images of HepG2 CDXs (H), tumor volumes (I) and tumor weight (J) from nude mice treated with **Tc3** (20 mg/kg and 40 mg/kg) and control medium (n = 6). (K) Immunoblot analysis of GSDME activation in HepG2 CDXs from three nude mice treated with **Tc3** and control medium. (L-N) Representative immunohistochemical (IHC) images of Ki67 and GSDME in HepG2 CDXs from nude mice treated with **Tc3** (L). Scale bars: 100 μm. Statistical analysis of Ki67 (M) and GSDME (N) levels detected by IHC. (O) Blood routine examination of nude mice bearing HepG2 CDXs after **Tc3** treatment, including statistical analysis of leukocytes, lymphocytes, monocytes and neutrophils. Data are presented as mean ± SD. *p < 0.05, **p < 0.01, ***p < 0.001 and ****p < 0.0001, ns, no significant.

Further, we performed hepatocarcinoma xenograft treatment assay in Balb/c nude mice. In the hepatocarcinoma PDXs mouse model, **Tc3** (20 mg/kg and 40 mg/kg) was injected (i.p.) ten times and the mice were sacrificed on day 20 (Figure 6A). Remarkably, **Tc3** treatment significantly reduced the tumor size and weight (Figure 6B-D). And GSDME was up-regulated and activated by** Tc3** in tumors, causing pyroptosis of cancer cells (Figure 6E), consistent with the results of blood routine examinations of the mice (Figure 6F). In the HepG2 cell line-derived xenografts (CDXs) mouse model, **Tc3** (20 mg/kg and 40 mg/kg) was dosed (i.p.) five times and the mice were sacrificed (Figure 6G). The growth rate and weight of tumors treated with **Tc3** were significantly smaller compared to the vehicle group (Figure 6H-J). Correspondingly, the levels of GSDME in tumors increased, and the caspase3-GSDME pathway was activated in the **Tc3** treatment group compared to the vehicle group as shown by western blotting and immunohistochemical staining (IHC) assays (Figure 6K-N). Meanwhile, the levels of Ki67 decreased, which indicated the reduced growth rate of tumors (Figure 6L-M). Blood routine examinations of the mice showed that the immune system was activated and the inflammatory response was enhanced with increasing concentrations of **Tc3**, even in Blab/c nude mice with severe immune deficiency (Figure 6O). In conclusion, **Tc3** exhibited significant anti-tumor activity via inducing pyroptosis and affecting the immune system of mice at a dose of 20 mg/kg.

### Injection of Tc3 enhances the efficacy of cisplatin against hepatic carcinoma* in vivo*

**Figure 7:**
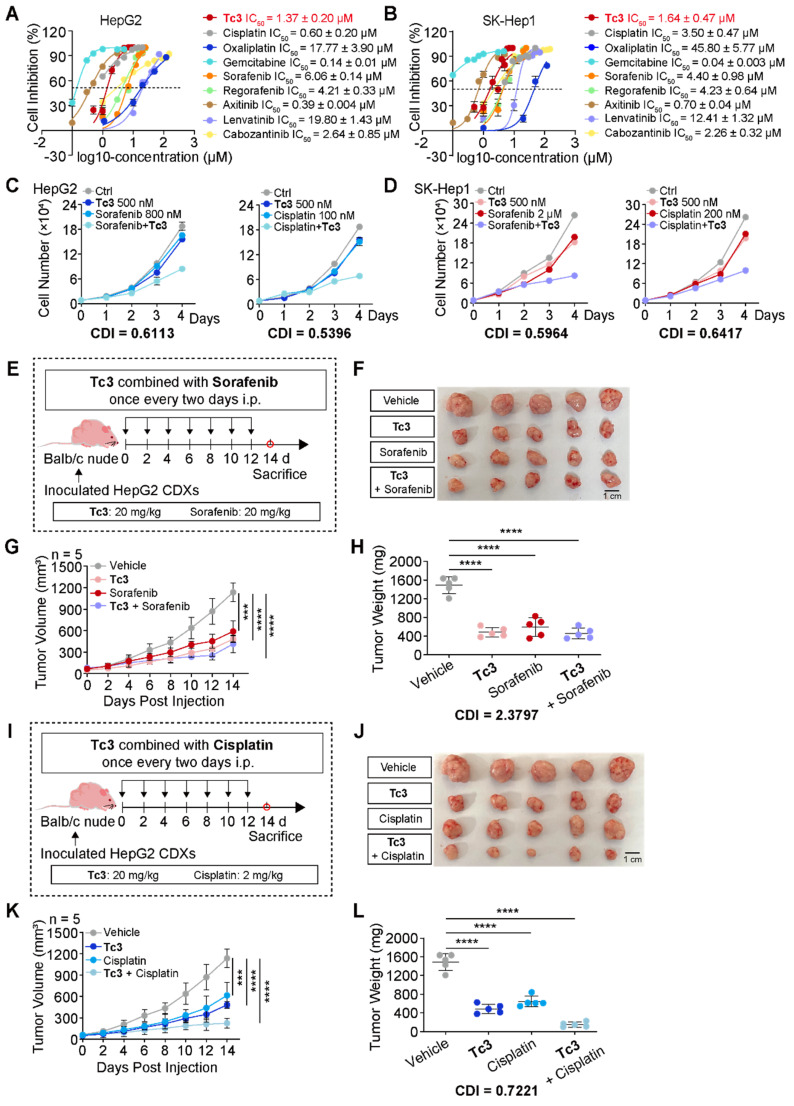
** Tc3 synergizes the anti-tumor effect of cisplatin *in vivo*.** (A-B) Dose-response curves for **Tc3** and other clinical drugs inhibiting the growth of HepG2 (A) and SK-Hep1 (B) cells. (C-D) Combination effects of **Tc3** with cisplatin and sorafenib in HepG2 (C) and SK-Hep1 (D) cells. (E) Schematic depicting the process of **Tc3** combined with sorafenib for treating HepG2 CDXs in Balb/c nude mice (n = 5). (F-H) Images of HepG2 CDXs (F), tumor volumes (G) and tumor weight (H) from nude mice treated with **Tc3** (20 mg/kg) combined with sorafenib (20 mg/kg) (n = 5). (I) Schematic depicting the process of **Tc3** combined with cisplatin for treating HepG2 CDXs in Balb/c nude mice (n = 5). (J-L) Images of HepG2 CDXs (J), tumor volumes (K) and tumor weight (L) from nude mice treated with **Tc3** (20 mg/kg) combined with cisplatin (2 mg/kg) (n = 5). Data are presented as mean ± SD. ***p < 0.001 and ****p < 0.0001.

To comprehensively assess the anti-tumor potency of **Tc3**, we compared it with several clinical chemotherapy drugs such as cisplatin, oxaliplatin, gemcitabine, and some first-line targeted anticancer drugs such as sorafenib, regorafenib, axitinib, lenvatinib, and cabozantinib, which were used for hepatocarcinoma treatment. The IC_50_ values of these drugs were determined to select appropriate concentrations. Noteworthy, **Tc3** showed great anti-cancer potency among these clinical drugs *in vitro* (Figure 7A-B). Then, we attempted to evaluate their synergistic potential with **Tc3** for hepatocarcinoma treatment to develop novel combination therapy.

We next verified the combination therapy of **Tc3** and some clinical drugs in hepatocarcinoma cells. Interestingly, only cisplatin and sorafenib exhibited a synergistically anti-tumor effects with **Tc3** in HepG2 and SK-Hep1 cells (Figure 7C-D and S9A-L). Additionally, we detected the IC_50_ values of cisplatin and sorafenib in AML-12 cells, and verified the combination effect of cisplatin and sorafenib with **Tc3**, respectively (Figure S9M). As shown in Figure S9N-O, there exhibited no synergistic effects of cisplatin and sorafenib with **Tc3** in AML-12 cells. To further confirm these findings *in vivo*, we employed the HepG2 CDXs mouse model. Contrary to the *in vitro* results, sorafenib did not show a synergistic anti-tumor effect with **Tc3** *in vivo* (CDI=2.3797) (Figure 7E-H). This discrepancy may be due to the immune deficiency of Balb/c nude mice, which might impede **Tc3**'s ability to activate the immune system. Intriguingly, cisplatin demonstrated a significant synergistic anti-tumor effect with **Tc3** *in vivo* (CDI=0.7221) (Figure 7I-L). These results suggested that **Tc3** is a highly promising candidate for enhancing the efficacy of clinical drugs, such as cisplatin, or as a standalone treatment for hepatocarcinoma.

### Synergistic effect of Tc3 and anti-PD-1 on hepatic carcinoma via activating TIME

In addition to induce GSDME-mediated pyroptosis in tumor cells, **Tc3** also has the capability to modulate and activate the immune response against hepatic carcinoma. We hypothesized that **Tc3** inhibited tumor growth by activating anti-tumor immunity and it might have synergistic effect with anti-PD-1 antibody on hepatic carcinoma.

**Figure 8:**
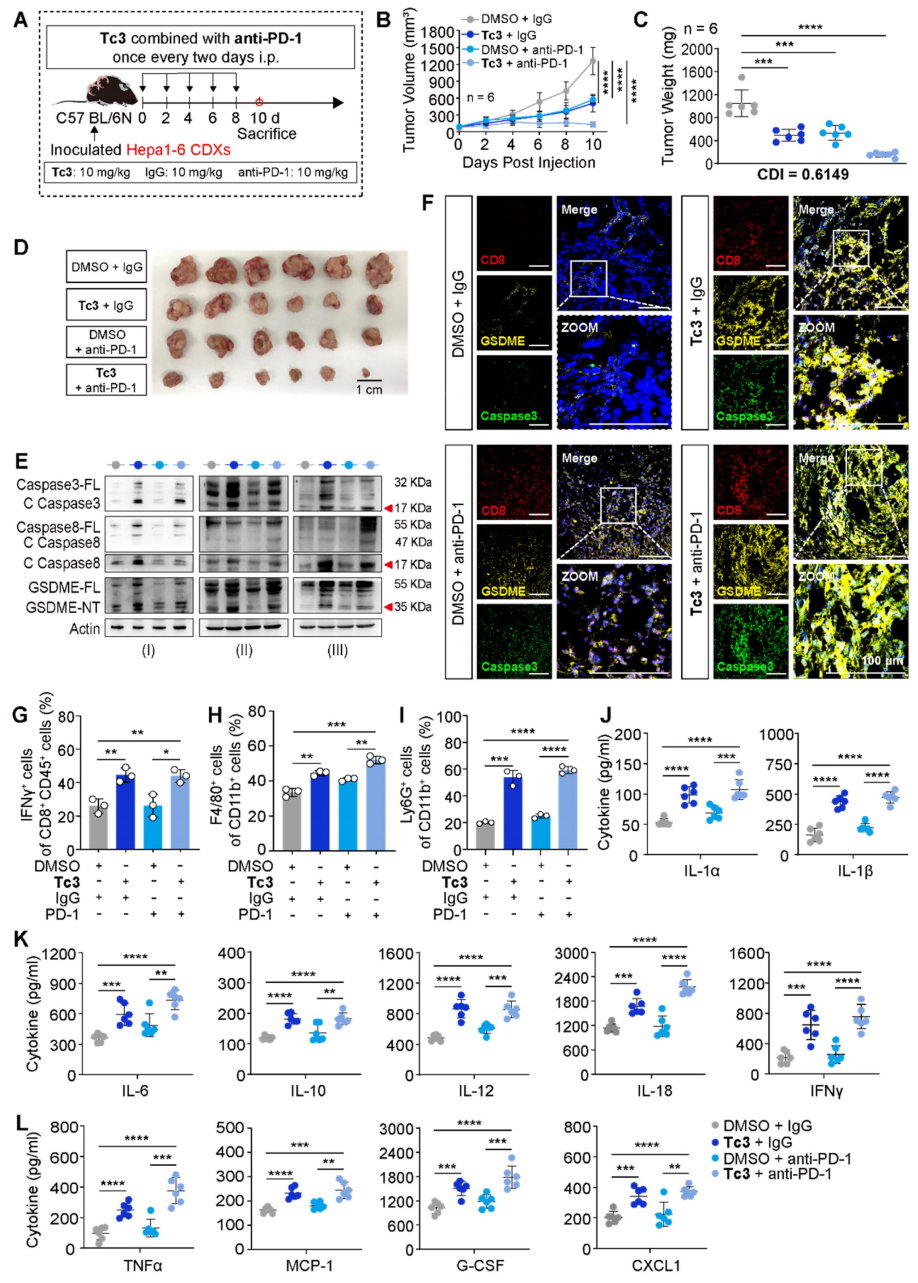
** Tc3 synergizes anti-PD-1 therapy to inhibit hepatoma* in vivo*.** (A) Schematic depicting the process of **Tc3** combined with anti-PD-1 antibody on tumors derived from Hepa1-6 cells in C57 BL/6N mice (n = 6). (B-D), Tumor volumes (B), tumor weight (C) and images of Hepa1-6 CDXs (D) from C57 mice treated with **Tc3** (10 mg/kg) combined with anti-PD-1 antibody (10 mg/kg). (E) Immunoblot analysis of the activation of caspase3, caspase8 and GSDME in Hepa1-6 CDXs from C57 mice treated by **Tc3** combined with anti-PD-1 antibody. (F) Representative fluorescence staining images showing the level and location of CD8, GSDME and caspase3 in tumor tissues. Scale bars: 100 μm. (G-I) Statistical analysis of flow cytometry data showing infiltration of IFNγ^+^CD8^+^ T cells (G), macrophages (H) and neutrophils (I) in tumor tissues derived from Hepa1-6 CDXs in C57 BL/6N mice (n=3). (J-L) Levels of cytokines IL-1α, IL-1β (J), IL-6, IL-10, IL-12, IL-18, IFNγ (K), TNFα, MCP-1, G-CSF and CXCL1 (L) in tumor tissues detected by ELISA (n=3). Data are presented as mean ± SD. *p < 0.05, **p < 0.01, ***p < 0.001 and ****p < 0.0001.

To better demonstrate the anti-tumor efficacy of **Tc3**, we employed a mouse model with Hepa1-6 xenografts. And mice were treated with** Tc3** and anti-PD-1 antibody. Hepa1-6 cells mixed with extracellular matrix gel were injected into the subcutaneous tissues of C57 BL/6N mice, leading to the formation of suitably sized hepatic carcinoma CDXs within five days. Groups of C57 mice bearing tumors received injections of **Tc3** (10 mg/kg), anti-PD-1 antibody (10 mg/kg) or the combination of two for five times, while control groups were dosed with DMSO or IgG antibodies (Figure 8A). A lower dosage of **Tc3** at 10 mg/kg was used to prevent severe inflammatory effects resulting from GSDME-mediated pyroptosis. Notably, intraperitoneal injections of **Tc3** alone or combined with anti-PD-1 antibodies significantly inhibited tumor growth compared to control groups. The combination of **Tc3** and anti-PD-1 antibody exhibited a significant synergistic effect on the treatment of Hepa1-6 CDXs (CDI=0.6149) (Figure 8B-D). Pathological studies showed that **Tc3** did not cause significant damage to the organs of the mice, including the liver, spleen, kidney and small intestine (Figure S10A).

**Figure 9:**
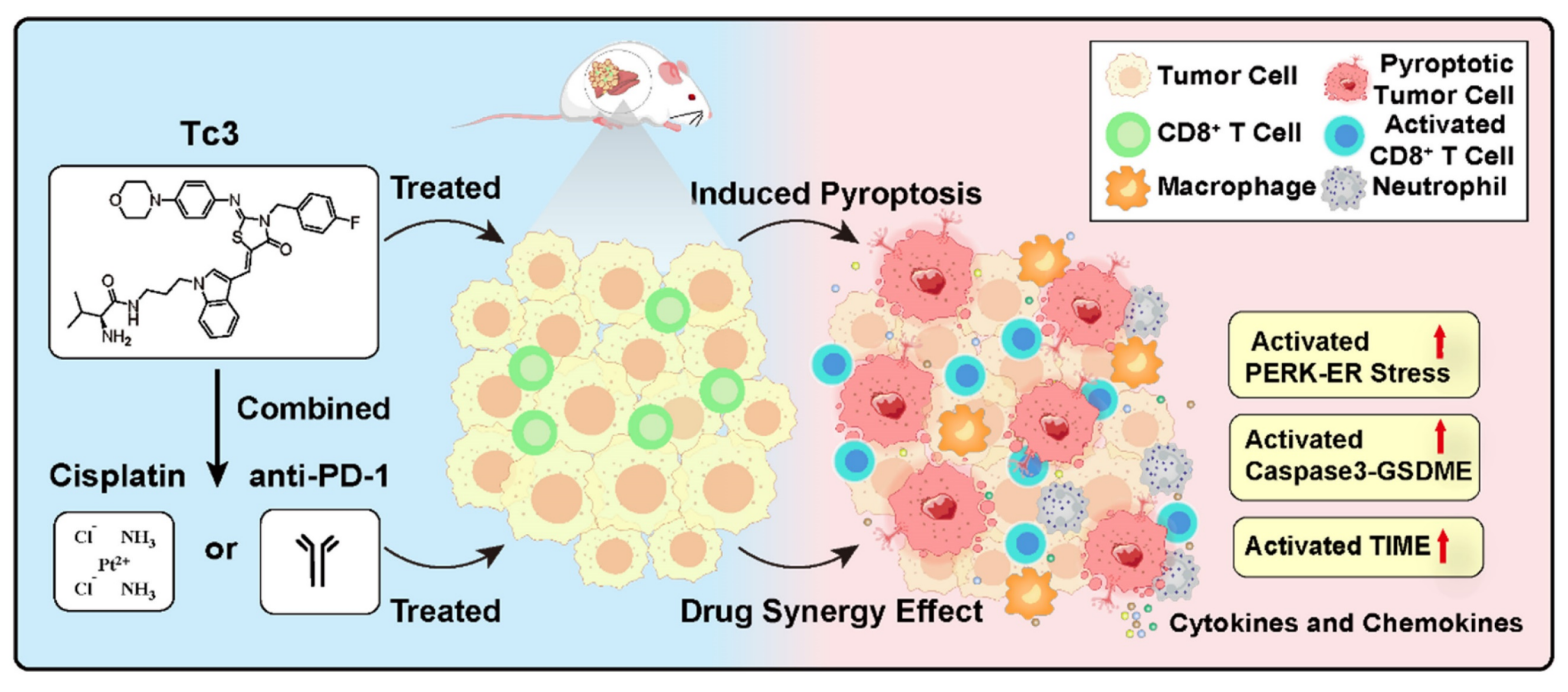
**Graphical abstract of Tc3 in hepatic carcinoma cells. Tc3** significantly triggers endoplasmic reticulum stress, leading to GSDME-mediated pyroptosis. Additionally, **Tc3** synergizes with cisplatin or anti-PD-1 antibody to inhibit hepatic carcinoma *in vivo*.

The caspase8-caspase3-GSDME pathway was activated by **Tc3** in the tumors of C57 mice (Figure 8E). Given the synergistic effect of **Tc3** and anti-PD-1 antibody, likely resulting from the activation of GSDME-mediated pyroptosis, we supposed that **Tc3** stimulated the TIME of hepatocarcinoma, thereby enhancing the efficacy of anti-PD-1 antibody. Triple-labelled fluorescence staining and flow cytometry analysis were conducted to determine the cell types induced by **Tc3**. Injection of **Tc3** significantly promoted the infiltration of activated CD8^+^ T cells (Figure 8F-G and S11A-B) and elevated the levels of cytokines (IL-1α, IL-1β, IL-6, IL-10, IL-12, IL-18, IFNγ, TNFα, MCP-1, G-CSF and CXCL1) (Figure 8J-L). Previous studies have reported that the inflammatory cytokines undergo secretion during pyroptosis of cells and activate the antitumor immunity in tumor microenvironment 33, 34. These results indicated that **Tc3** inhibits tumor growth primarily via activating CD8^+^ T cells and enhancing the activation of cytotoxic T lymphocytes induced by the anti-PD-1 antibody. Furthermore, the numbers of macrophages in tumors treated with **Tc3** or **Tc3** combined with anti-PD-1 antibodies were significantly higher than the control groups (Figure 8H and S10B). Compared to the M2 macrophages, the numbers of M1 macrophages were obviously up-regulated by **Tc3**, which could contribute to anti-tumor immunity (Figure S11C-D). Besides, the numbers of neutrophils were also higher in **Tc3** treated groups than the control group (Figure 8I, S10C and S11E). Of note, the level of GSDME was markedly activated and up-regulated by **Tc3** in tumors, co-localizing with activated caspase3 and infiltrative lymphocytes. Collectively, our findings suggest that **Tc3** inhibits tumor growth by effectively enhancing anti-tumor immunity (Figure 9).

## Discussion

In this study, we used **T1** as the lead compound and introduced different functional groups at the amino side chain, confirming that amino acid residues can improve the anti-tumor activity. While substitution of H with Br or OCH_3_ at the 5-position of indole demonstrated comparable anti-tumor potency, **Tb7**, with a Br at the 5-position of indole and L-isoleucine at the amino side chain, exhibited a nearly 9-fold increase in potency compared to **T1**. Furthermore, the addition of a 4-morpholinoaniline group to **T1** led to a significant enhancement in anti-tumor potency, with a nearly 3-fold improvement. Based on these observations, we synthesized four additional **Tc1** derivatives (**Tc2**, **Tc3**, **Tc4** and** Tc5**) with an amino acid group at the amino terminus. Compound **Tc3** emerged as the most potent agent, with an IC_50_ of 1.37 μM (HepG2). Further modifications at the 2-position of 2,4-thiazolidinedione were planned to obtain more potent agents. However, none of these compounds showed any significant improvement in anti-tumor activity. While **Tc3** showed slightly better potency than sorafenib, with IC_50_ values of 1.37 and 1.64 μM for HepG2 and SK-Hep1 cells respectively, compared to 6.06 and 4.40 μM for sorafenib (the first-line therapy for hepatocellular carcinoma), the rationale for these SAR trends is not yet clear. Further investigation on targets of **Tc3** is essential to explore the chemical diversity in this series. We demonstrate here for the first time that the design of thiazole-substituted indole compound **Tc3** as potent pyroptosis inducer, which has promising characteristics that could advance the treatment of hepatic carcinoma.

Pyroptosis as a novel inflammatory programmed cell death mediated by the gasdermins may act as a double-edged sword for tumors. The activation of pyroptosis leads to the release of inflammatory cytokines such as IL-1α and IL-1β, which induce chronic inflammation in tumor foci and are related to tumorigenesis and drug resistance 35, 36. Some clinical chemotherapy drugs like cisplatin and 5-FU can activate pyroptosis and cause chemotherapy-induced tissue damage 21, 22. On the contrary, inflammasomes induced by pyroptosis can facilitate the development of adaptive immune responses in the TIME, initiating immune cytotoxicity and inhibiting tumor cell proliferation, invasion and metastasis 37. This indicates the potential of pyroptosis as a tumor treatment strategy, although the mechanism between pyroptosis and cancer is not clearly understood at present.

As a significant member of the gasdermin family, GSDME is conserved in even earlier metazoa which probably exerts a highly specific function for cell survival and propagation 21, 38. In tumor cells, GSDME can inhibit cell proliferation and activate anti-tumor immunity, indicating that GSDME may function as a tumor suppressor 39-41. However, GSDME assists tumor escape via activating GSDME-YBX1-mucin axis in pancreatic ductal adenocarcinoma, suggesting that the function of GSDME may regulate gene expression and serve as a tumor promoter independent of pyroptosis 21, 42. Hence, the relationship between pyroptosis and cancer is complicated, and it is necessary to study the biochemical network of that to effectively exert the anti-tumor activity of pyroptosis. In this work, we attempt to activate GSDME and induce pyroptosis to inhibit hepatic carcinoma growth by **Tc3**. Additionally, it is also important to enliven the anti-tumor lymphocytes and cytokines in TIME via activated pyroptosis. Of note. **Tc3** combined with anti-PD-1 antibody amplified the anti-tumor effect of tumor immunotherapy, attempting to balance the relationship of pyroptosis and tumor-therapy (Figure 8D-F). Following cleavage by caspase3, GSDME can induce tumor cell pyroptosis and enhance the anti-tumor immunity of T cells. It is reported that the level of GSDME is a key factor determining whether caspase3-activated cells undergo apoptosis or pyroptosis 43. Although GSDME is often low-expressed in various tumor cells, the mRNA and protein level of GSDME remain normal in hepatic carcinoma cells according to our work, which is crucial for the anti-tumor effect of **Tc3**. GSDME was up-regulated in hepatic carcinoma cells by **Tc3**, indicating that **Tc3** may overcome the restrictions of GSDME expression at the protein level.

Previous studies regarded that GSDME is silenced in many tumor cells, which can be achieved through epigenetic suppression or loss-of-function mutations in *DFNA5* 39, 44. It indicates that the combination of **Tc3** with DNA-demethylating drugs such as distamine might potentially enhance the anti-tumor activity of **Tc3** in other tumors. GSDME plays an important part in increasing the anti-tumor functions of tumor-infiltrating NK and CD8^+^ T cells, offering new inspiration for pyroptosis-mediated cancer treatments. Similarly, our data demonstrated that **Tc3** could activate the TIME in mice and increase tumor-infiltrating lymphocytes, which is worthy of consideration as a potent pyroptosis inducer and GSDME activator for inhibiting hepatic carcinoma. The cellular morphology of hepatic carcinoma cells treated by **Tc3** showed cytoplasmic vacuoles and larger endoplasmic reticulum, in addition to swelling cell membranes and bubble-like protrusions, indicating ER stress. RNA sequencing data also showed ER stress and UPR effects were emerged in hepatic carcinoma induced by **Tc3**. It is hypothesized that the reactive oxygen species (ROS) may be induced by **Tc3**, further stimulating the ER and triggering GSDME-mediated pyroptosis in hepatic carcinoma cells. However, the molecular mechanism and binding target of **Tc3** remain unclear. Further investigation of **Tc3** and its analogues is significant.

Taken together, we designed and developed a series of indole analogues based on compound **T1** and evaluated the anticancer activity of 26 novel small molecules. The data in this study showed that **Tc3** emerged as a promising lead compound, exhibiting potent induction of GSDME-mediated pyroptosis both *in vitro* and *in vivo* in hepatic carcinoma via activated PERK and the ER stress. Additionally, **Tc3** significantly improved the anti-tumor potency of cisplatin* in vivo*. Moreover, **Tc3** also had syngeneic effects with immune checkpoint inhibitor anti-PD-1 antibodies, by virtue of its induction of pyroptosis in hepatic carcinoma. Notably, **Tc3** exhibited dual functions in cancer therapy. One is to motivate the pore-forming activity of GSDME to trigger tumor cell pyroptosis; another is to activate anti-tumor immune response. Specifically, this work first put forward thiazole-substituted indole **Tc3** as pyroptosis inducer and identified its novel combination therapy for hepatic carcinoma. We provide a rationale to develop new therapeutic options to evoke the response of immunotherapy in hepatic carcinoma. Work along these lines is currently ongoing in our group.

## Supplementary Material

Supplementary methods, figures and tables.

## Abbreviations

CDXs: cell-derived xenografts
DEGs: differentially expressed genes
ER stress: endoplasmic reticulum stress
GSD: Gasdermin
HE: hematoxylin-eosin
LDH: lactate dehydrogenase
PDXs: patient-derived xenografts
PERK: protein kinase R-like ER kinase
TIME: tumor immune microenvironment
UPR: unfolded protein response
