# Supplementary material for: Discovery of indole analogue Tc3 as a potent pyroptosis inducer and identification of its combination strategy against hepatic carcinoma
Source: Theranostics. 2025 Jan 1;15(4):1285–303. doi: 10.7150/thno.102228 (PMC11729550; doi:10.7150/thno.102228)
Supplement: Supplementary file 1 — Supplementary methods, figures and tables. [file thnov15p1285s1.zip › chemical supporting information.docx]

# Part A. Supporting Tables

Table s1. The anti-tumor activity of **Tb7**, **Tc1**, **Tc3** and **Tc4** against hepatic carcinoma cells. *^a^*

| Compd | R_1_ | X | R_2_ | R_3_ | IC_50_ (μM) *^a^* | | |
| --- | --- | --- | --- | --- | --- | --- | --- |
|  |  |  |  |  | HepG2 | SK-Hep1 | PLC-PRF5 |
| **Tb7** | / | O | Br |  | 1.79±0.31 | 2.21±0.17 | 1.26±0.31 |
| **Tc1** |  | N | H | H | 5.13±0.53 | 2.87±0.61 | 2.71±0.12 |
| **Tc3** |  | N | H |  | 1.37±0.20 | 1.64±0.47 | 1.79±0.57 |
| **Tc4** |  | N | H |  | 2.46±0.34 | 2.30±0.14 | 2.92±0.32 |

^a^ Each value represents the average result of three independent experiments.

# Part B. Synthesis and NMR Data of of Ta1-Ta5, Tb1-Tb9 and Tc1-Tc12

**General Methods for Chemistry.** Commercially available reagents and solvents were used without further purification. Thin layer chromatography (TLC) was performed on precoated silica gel 60 F_254_ plates, and spots were visualized with UV light (254 and 354 nm). Flash column chromatography was performed on silica gel (200-300 mesh). NMR spectra were recorded on Bruker spectrometers (^1^H at 400 MHz and ^13^C at 101 MHz) in CD_3_SOCD_3_ solutions, and chemical shifts (δ) were reported as parts per million (ppm) with reference to solvent signals. Proton spectra were reported as chemical shift, multiplicity (s = singlet, d = doublet, t = triplet, q = quartet, m = multiplet, and br = broad), coupling constant (*J*) in Hz, and number of protons. Carbon spectra were reported as chemical shift alone. High-resolution mass measurements (HRMS) for compound characterization were carried out using Thermo Scientific Orbitrap Fusion Lumos Tribrid. High Performance Liquid Chromatography (HPLC) was performed using the general method: equipment = Agilent 1260 HPLC; column = Phenomenex Luna C18 5micron column (250 mm × 4.60 mm, 5 μm); tested temporary = 35 °C; solvent = MeOH/0.1% TFA dissolved in H_2_O; Gradient = 10-90% MeOH in 0.1% TFA solution at 1 mL/min flow rate; detector = The UV detection at 254 nm and 270 nm. All reactions requiring heating were heated in an oil bath. All reactions sensitive to air or moisture were conducted under argon atmosphere in dry and freshly distilled solvents, unless otherwise noted.

Scheme s1. Synthetic route of indole analogues **Ta1**-**Ta5**, **Tb1**-**Tb9** and **Tc1**-**Tc12**. *^a^*

*^a^* Reagents and conditions: (a) 1,1'-Thiocarbonyldiimidazole (TCDI), DCM and THF , r.t., 2 h, 91%; (b) (4-Fluorophenyl)methanamine, EA, r.t., 17 h, 70%; (c) Chloroacetic acid, sodium acetate, EtOH, 80 ^o^C, reflux, 12 h, 53%; (d) K_2_CO_3_, N_2_, 4-fluorobenzyl bromide, DMF, 80 ^o^C, 3 h, 50%; (e) piperidine, Indole-3-carboxaldehyde, EtOH, 80 ^o^C, 12 h, 60%; (f) NaH, tert-butyl (3-bromopropyl)carbamate, DMF, 80 ^o^C, 4 h, 86%; (g) TFA, DCM, r.t., 2 h, 70%; (h) R_3_, n-(3-dimethylaminopropyl)-n'-ethylcarbodiimide hydrochloride (EDCI), 1-Hydroxybenzotriazole (HOBT), TEA, DCM, r.t., 12 h, 50%; (i) TFA, DCM, r.t., 2 h, 85%.

General Procedure for the Synthesis of **2**. **1** (1.00 equiv) in DCM was added to the solution of 1,1'-Thiocarbonyldiimidazole (1.01 equiv) in THF. The mixture was stirred at room temperature for 2 h. After that, the reaction was poured into water and extracted with DCM. The combined organic phase was washed with brine, dried over Na_2_SO_4_, filtered, and concentrated under reduced pressure to afford a crude, which was purified by column chromatography (PE/EtOAc = 3:1 to 1:1 v/v) to give the compound **2** in about 91 % yield.

General Procedure for the Synthesis of **3**. (4-Fluorophenyl)methanamine (1.10 equiv) was added to a solution of **2** (1.00 equiv) in EA. The reaction was stirred at room temperature for 17 h. After that, the mixture was filtered, the filter cake was washed with EA and dried to get white solid powder **3** in about 70 % yield.

General Procedure for the Synthesis of **4a**. Chloroacetic acid (1.25 equiv) and sodium acetate (2.00 equiv) were added to the solution of **3** (1.00 equiv) in EtOH, and the reaction was stirred at 80 ^o^C for 12 h. After cooling to room temperature, the mixture was poured into water and extracted with EtOAc. The combined organic phases were washed with brine, dried over Na_2_SO_4_, filtered, and concentrated under reduced pressure to afford a crude, which was purified by column chromatography (DCM/MeOH = 50:1 v/v) to give a yellow solid **4a** in about 53 % yield.

General Procedure for the Synthesis of **4b**. The solution of 2,4-Thiazolidinedione (1.00 equiv), K_2_CO_3_ (1.00 equiv) in DMF was purged with N_2_. After that, 4-fluorobenzyl bromide (1.20 equiv) was added dropwise, and the reaction was stirred at 80 ^o^C for 3 h. After cooling to room temperature, the mixture was poured into water and extracted with EtOAc. The combined organic phases were washed with brine, dried over Na_2_SO_4_, filtered, and concentrated under reduced pressure to afford a crude, which was purified by column chromatography (PE/EtOAc = 5:1 to 3:1 v/v) to give a yellow solid **4b** in about 50 % yield.

General Procedure for the Synthesis of **5a/5b**. Intermediate **4a**/**4b** (1.00 equiv) was added to a solution of 5-substituted indole-3-carboxaldehyde (1.00 equiv), a catalytic amount of piperidine (0.10 equiv) in EtOH. The reaction was heated to 80℃ and refluxed overnight. The mixture was filtered, the filter cake was washed with EtOH and dried to get yellow solid powder **5a**/**5b**, yield of about 60 %.

General Procedure for the Synthesis of **6a/6b**. Tert-butyl (3-bromopropyl)carbamate (1.21 equiv) was added to a solution of **5a**/**5b** (1.00 equiv) and NaH (1.34 equiv) in ultra-dry DMF at room temperature. After addition, the reaction was carried out at 80 °C for 4 h. After cooling to room temperature, the mixture was poured into water and extracted with EtOAc. The combined organic phase was washed with brine, dried over Na_2_SO_4_, filtered, and concentrated under reduced pressure to afford the intermediates. Immediately after this, the yellow solid intermediates were dissolved in ultra-dry DCM, followed by the addition of TFA (15.00 equiv). The solution was stirred at room temperature for 2 h. After the reaction was completed, NaHCO_3_ was used to adjust pH to 7, and the mixture was extracted with DCM. The combined organic phase was washed with brine, dried (Na_2_SO_4_), filtered and concentrated. The crude product obtained was purified by column chromatography (DCM/MeOH =100:1 v/v) to obtain yellow solid **6a**/**6b** in about 70 % yield.

General Procedure for the Synthesis of final compounds. TEA (1.25 equiv) was added to a solution of N-Boc-L-amino acid (1.25 equiv), EDCI (1.25 equiv), HOBT (1.25 equiv) in ultra-dry DCM . The mixture was stirred for 30 min at room temperature. Intermediate **6a**/**6b** (1.00 equiv) dissolving in ultra-dry DCM was then added dropwise and the reaction was stirred overnight at room temperature. After that, the mixture was poured into water and extracted with DCM. The combined organic phases were washed with brine, dried over Na_2_SO_4_, filtered, and concentrated under reduced pressure to afford a residue. Which was purified by column chromatography (PE/EA = 2:1 to 1:1 v/v) to give a yellow solid intermediate. The intermediate (1.00 equiv) was dissolved in ultra-dry DCM, followed by the addition of TFA (15.00 equiv). The mixture was stirred at room temperature for 2 h. After the reaction was completed, NaHCO_3_ was used to adjust pH to 7, and the mixture was extracted with DCM. The combined organic phase was washed with brine, dried (Na_2_SO_4_), filtered and concentrated. The crude product obtained was purified by column chromatography (DCM/MeOH =50:1 to 25:1 v/v) to give the title compounds in a yield of about 85 %.

*(Z)-N-(3-(3-((3-(4-fluorobenzyl)-2,4-dioxothiazolidin-5-ylidene)methyl)-1H-indol-1-yl)P-ropyl)cyclo-pent-anecarboxamide* ***(Ta1)****.* Yellow solid. Yields: 74% (200 mg, 0.40 mmol). ^1^H NMR (400 MHz, DMSO-*d6*) δ 8.19 (s, 1H), 7.97 – 7.91 (m, 2H), 7.86 (t, *J* = 4.6 Hz, 1H), 7.61 (d, *J* = 8.1 Hz, 1H), 7.41 – 7.36 (m, 2H), 7.32 (t, *J* = 7.5 Hz, 1H), 7.25 (t, *J* = 7.4 Hz, 1H), 7.19 (t, *J* = 8.8 Hz, 2H), 4.83 (s, 2H), 4.34 (t, *J* = 6.5 Hz, 2H), 3.05 (q, *J* = 6.0 Hz, 2H), 1.96 – 1.88 (m, 2H), 1.77 – 1.70 (m, 2H), 1.61 (s, 5H), 1.49 (s, 2H). ^13^C NMR (101 MHz, DMSO-*d6*) δ 175.83, 167.63, 165.86, 160.92, 136.43, 132.58, 132.47, 130.44, 130.35, 127.91, 126.13, 123.78, 122.02, 119.28, 116.05, 115.84, 114.00, 111.39, 110.15, 44.84, 44.63, 44.24, 36.43, 30.47, 30.10, 26.06. C_28_H_28_N_3_O_3_FS, HRMS calculated for m/z [M+Na]^+^: 528.1733(calculated), 528.1730(found).

*(Z)-3-cyclopentyl-N-(3-(3-((3-(4-fluorobenzyl)-2,4-dioxothiazolidin-5-ylidene)methyl)-1H-indol-1-yl)prop-yl)propenamide* ***(Ta2)****.* Yellow solid. Yields: 78% (176 mg, 0.33 mmol). ^1^H NMR (400 MHz, DMSO-*d6*) δ 8.19 (s, 1H), 7.96 – 7.91 (m, 2H), 7.88 (t, *J* = 5.0 Hz, 1H), 7.61 (d, *J* = 8.1 Hz, 1H), 7.41 – 7.35 (m, 2H), 7.32 (t, *J* = 7.5 Hz, 1H), 7.25 (t, *J* = 7.4 Hz, 1H), 7.19 (t, *J* = 8.8 Hz, 2H), 4.82 (s, 2H), 4.34 (t, *J* = 6.5 Hz, 2H), 3.05 (q, *J* = 6.0 Hz, 2H), 2.08 (t, *J* = 7.6 Hz, 2H), 1.95 – 1.88 (m, 2H), 1.74 – 1.65 (m, 3H), 1.50 (dq, *J* = 21.3, 14.2, 10.7 Hz, 6H), 1.04 (s, 2H). ^13^C NMR (101 MHz, DMSO-*d6*) δ 172.79, 167.61, 165.85, 160.91, 136.43, 132.59, 132.43, 130.45, 130.36, 127.92, 126.10, 123.75, 122.01, 119.26, 116.04, 115.83, 113.99, 111.38, 110.14, 44.68, 44.23, 36.34, 35.29, 32.49, 32.08, 30.06, 25.13. C_30_H_32_N_3_O_3_FS, HRMS calculated for m/z [M+Na]^+^: 556.2046(calculated), 556.2050(found).

*(Z)-N-(3-(3-((3-(4-fluorobenzyl)-2,4-dioxothiazolidin-5-ylidene)methyl)-1H-indol-1-yl)propyl)isobutyram-ide* ***(Ta3)****.* Yellow solid. Yields: 71% (185 mg, 0.39 mmol). ^1^H NMR (400 MHz, DMSO-*d6*) δ 8.19 (s, 1H), 7.96 – 7.90 (m, 2H), 7.83 (t, *J* = 5.0 Hz, 1H), 7.61 (d, *J* = 8.2 Hz, 1H), 7.38 (dd, *J* = 8.3, 5.6 Hz, 2H), 7.32 (t, *J* = 7.5 Hz, 1H), 7.25 (t, *J* = 7.5 Hz, 1H), 7.19 (t, *J* = 8.8 Hz, 2H), 4.83 (s, 2H), 4.34 (t, *J* = 6.7 Hz, 2H), 3.05 (q, *J* = 6.1 Hz, 2H), 2.35 (dt, *J* = 13.6, 6.8 Hz, 1H), 1.92 (p, *J* = 6.5 Hz, 2H), 1.01 (d, *J* = 6.8 Hz, 6H). ^13^C NMR (101 MHz, DMSO-*d6*) δ 176.68, 167.64, 165.85, 163.30, 136.43, 132.49, 130.45, 130.40, 130.32, 127.90, 126.11, 123.77, 122.00, 119.23, 116.02, 115.83, 115.79, 113.99, 113.95, 111.35, 110.13, 44.62, 44.22, 36.30, 34.56, 34.51, 30.08, 20.06, 20.01. C_26_H_26_N_3_O_3_FS, HRMS calculated for m/z [M+Na]^+^: 502.1577(calculated), 502.1579(found).

*(Z)-N-(3-(3-((3-(4-fluorobenzyl)-2,4-dioxothiazolidin-5-ylidene)methyl)-1H-indol-1-yl)propyl)-4-methylp-entanamide* ***(Ta4)****.* Yellow solid. Yields: 75% (165 mg, 0.33 mmol). ^1^H NMR (400 MHz, DMSO-*d6*) δ 8.18 (s, 1H), 7.94 – 7.85 (m, 3H), 7.60 (d, *J* = 8.1 Hz, 1H), 7.38 (dd, *J* = 8.1, 5.7 Hz, 2H), 7.31 (t, *J* = 7.5 Hz, 1H), 7.24 (t, *J* = 7.4 Hz, 1H), 7.18 (t, *J* = 8.8 Hz, 2H), 4.81 (s, 2H), 4.33 (t, *J* = 6.6 Hz, 2H), 3.07 (q, *J* = 6.1 Hz, 2H), 2.09 (t, *J* = 7.7 Hz, 2H), 1.99 – 1.87 (m, 2H), 1.50 (dt, *J* = 13.1, 6.5 Hz, 1H), 1.40 (q, *J* = 7.1 Hz, 2H), 0.85 (d, *J* = 6.5 Hz, 6H). ^13^C NMR (101 MHz, DMSO-*d6*) δ 172.92, 167.58, 165.82, 163.36, 160.94, 136.45, 132.56, 132.53, 132.38, 130.47, 130.38, 127.92, 126.07, 123.73, 121.98, 119.20, 116.01, 115.80, 113.99, 111.34, 110.18, 44.68, 44.23, 36.38, 34.79, 34.01, 30.08, 27.72, 22.71. C_28_H_30_N_3_O_3_FS, HRMS calculated for m/z [M+Na]^+^: 530.1890(calculated), 530.1891(found).

*(Z)-N-(3-(3-((3-(4-fluorobenzyl)-2,4-dioxothiazolidin-5-ylidene)methyl)-1H-indol-1-yl)propyl)octanamie* ***(Ta5)****.* Yellow solid. Yields: 74% (173 mg, 0.32 mmol). ^1^H NMR (400 MHz, DMSO-*d6*) δ 8.19 (s, 1H), 7.94 (d, *J* = 10.7 Hz, 2H), 7.89 (t, *J* = 5.3 Hz, 1H), 7.61 (d, *J* = 8.1 Hz, 1H), 7.39 – 7.36 (m, 2H), 7.32 (s, 1H), 7.26 (d, *J* = 7.6 Hz, 1H), 7.18 (d, *J* = 8.8 Hz, 2H), 4.82 (s, 2H), 4.34 (t, *J* = 6.7 Hz, 2H), 3.05 (d, *J* = 5.9 Hz, 2H), 2.07 (t, *J* = 7.4 Hz, 2H), 1.93 – 1.89 (m, 2H), 1.51 – 1.47 (m, 2H), 1.23 (s, 8H), 0.83 (s, 3H). ^13^C NMR (101 MHz, DMSO-*d6*) δ 172.75, 167.61, 165.85, 163.33, 160.91, 136.42, 132.58, 132.43, 130.45, 130.36, 127.92, 126.10, 123.75, 122.01, 119.25, 116.04, 115.82, 113.98, 111.38, 110.14, 44.66, 44.23, 36.31, 35.93, 31.65, 30.12, 29.12, 28.93, 25.76, 22.53, 14.39. C_30_H_34_N_3_O_3_FS, HRMS calculated for m/z [M+Na]^+^: 558.2203(calculated), 558.2203(found).

*(S,Z)-2-amino-N-(3-(3-((3-(4-fluorobenzyl)-2,4-dioxothiazolidin-5-ylidene)methyl)-1H-indol-1-yl)pr-opyl)propenamide* ***(Tb1).*** Yellow solid. Yields: 81% (176 mg, 0.37 mmol). ^1^H NMR (400 MHz, DMSO-*d6*) δ 8.64 (t, *J* = 5.5 Hz, 1H), 8.22 (s, 2H), 8.18 (s, 1H), 7.93 (d, *J* = 5.7 Hz, 2H), 7.63 (d, *J* = 8.2 Hz, 1H), 7.39 – 7.35 (m, 2H), 7.31 (ddd, *J* = 8.3, 7.0, 1.2 Hz, 1H), 7.26 – 7.22 (m, 1H), 7.20 – 7.15 (m, 2H), 4.81 (s, 2H), 4.37 (t, *J* = 6.9 Hz, 2H), 3.85 (s, 1H), 3.15 – 3.10 (m, 2H), 1.96 (p, *J* = 7.0 Hz, 2H), 1.35 (d, *J* = 7.0 Hz, 3H). ^13^C NMR (101 MHz, DMSO-*d6*) δ 170.02, 167.60, 165.84, 163.33, 160.91, 136.44, 132.56, 132.53, 132.34, 130.44, 130.36, 127.89, 126.09, 123.79, 122.02, 119.23, 116.02, 115.80, 114.09, 111.42, 110.20, 49.03, 48.74, 44.47, 44.24, 36.61, 29.87, 17.60. C_25_H_25_N_4_O_3_FS, HRMS calculated for m/z [M+H]^+^: 481.1735 (calculated), 481.1730 (found).

*(S,Z)-2-amino-N-(3-(3-((3-(4-fluorobenzyl)-2,4-dioxothiazolidin-5-ylidene)methyl)-1H-indol-1-yl)propyl)-3-methylbutanamide* ***(Tb2)****.* Yellow solid. Yields: 75% (165 mg, 0.32 mmol). ^1^H NMR (400 MHz, DMSO-*d6*) δ 8.64 – 8.56 (m, 1H), 8.19 (d, *J* = 1.8 Hz, 1H), 7.94 (d, *J* = 7.4 Hz, 2H), 7.64 (d, *J* = 8.2 Hz, 1H), 7.40 – 7.35 (m, 2H), 7.32 (t, *J* = 7.7 Hz, 1H), 7.25 (t, *J* = 7.5 Hz, 1H), 7.18 (t, *J* = 8.7 Hz, 2H), 4.82 (s, 2H), 4.39 (t, *J* = 6.9 Hz, 2H), 3.52 (d, *J* = 6.0 Hz, 1H), 3.16 (p, *J* = 7.1 Hz, 2H), 2.05 (q, *J* = 6.8 Hz, 1H), 1.97 (q, *J* = 6.8 Hz, 2H), 0.93 (dd, *J* = 6.9, 1.8 Hz, 6H). ^13^C NMR (101 MHz, DMSO-*d6*) δ 168.38, 167.64, 165.83, 160.89, 136.43, 132.41, 130.43, 130.34, 127.88, 126.07, 123.75, 121.98, 120.73, 119.15, 117.83, 115.99, 115.77, 114.92, 114.03, 112.02, 111.47, 110.14, 58.07, 44.58, 44.20, 36.48, 30.10, 29.89, 18.77, 18.46. C_27_H_29_N_4_O_3_FS, HRMS calculated for m/z [M+H]^+^: 509.2023 (calculated), 509.2025 (found).

*(S,Z)-N-(3-(3-((3-(4-fluorobenzyl)-2,4-dioxothiazolidin-5-ylidene)methyl)-1H-indol-1-yl)propy)pyrrolidin-e-2-carboxamide* ***(Tb3)****.* Yellow solid. Yields: 78% (140 mg, 0.28 mmol). ^1^H NMR (400 MHz, DMSO-*d6*) δ 8.70 (d, *J* = 5.5 Hz, 1H), 8.59 – 8.49 (m, 1H), 8.18 (s, 1H), 7.91 (d, *J* = 4.6 Hz, 1H), 7.63 (d, *J* = 8.2 Hz, 1H), 7.34 (dt, *J* = 23.0, 7.5 Hz, 3H), 7.20 (dt, *J* = 17.4, 7.9 Hz, 3H), 4.81 (s, 2H), 4.37 (t, *J* = 7.0 Hz, 2H), 4.01 (q, *J* = 7.2 Hz, 1H), 3.26 – 3.19 (m, 2H), 3.16 (d, *J* = 8.6 Hz, 2H), 2.28 (dt, *J* = 12.4, 7.0 Hz, 1H), 1.99 (d, *J* = 8.2 Hz, 3H), 1.87 (q, *J* = 14.6, 10.7 Hz, 3H). ^13^C NMR (101 MHz, DMSO-*d6*) δ 168.63, 167.62, 165.84, 160.90, 136.44, 132.52, 132.38, 130.42, 130.34, 127.86, 126.09, 123.77, 121.99, 121.04, 119.15, 118.12, 115.99, 115.77, 115.20, 114.03, 112.28, 111.43, 110.16, 59.40, 48.96, 45.89, 44.49, 36.80, 30.05, 29.71, 24.00. C_27_H_27_N_4_O_3_FS, HRMS calculated for m/z [M+H]^+^: 507.1866 (calculated), 507.1867 (found).

*(S,Z)-N-(3-(5-bromo-3-((3-(4-fluorobenzyl)-2,4-dioxothiazolidin-5-ylidene)methyl)-1H-indol-1-yl)propyl)-pyrrolidine-2-carboxamide* ***(Tb4).*** Yellow solid. Yields: 84% (135 mg, 0.23 mmol). ^1^H NMR (400 MHz, DMSO-*d6*) δ 8.19 (s, 1H), 7.87 (d, *J* = 7.9 Hz, 2H), 7.59 (s, 1H), 7.27 (d, *J* = 8.7 Hz, 1H), 7.09 (d, *J* = 8.8 Hz, 1H), 7.06 – 7.00 (m, 2H), 6.84 (t, *J* = 8.6 Hz, 2H), 4.48 (s, 2H), 4.03 (t, *J* = 6.9 Hz, 2H), 3.82 (t, *J* = 6.9 Hz, 1H), 2.88 (t, *J* = 6.9 Hz, 2H), 2.80 (q, *J* = 6.9 Hz, 2H), 2.17 (s, 2H), 1.93 (dt, *J* = 12.9, 7.0 Hz, 1H), 1.62 (t, *J* = 6.9 Hz, 2H), 1.57 – 1.50 (m, 2H). ^13^C NMR (101 MHz, DMSO-*d6*) δ 168.60, 167.52, 165.73, 160.93, 135.24, 133.16, 132.52, 130.47, 130.39, 129.63, 126.33, 125.88, 122.07, 116.04, 115.83, 115.02, 114.86, 113.45, 109.95, 59.53, 46.04, 44.67, 44.27, 36.84, 29.94, 29.76, 24.01. C_27_H_26_N_4_O_3_BrFS, HRMS calculated for m/z [M+H]^+^: 585.0971 (calculated), 585.0971 (found).

*(S,Z)-2-amino-N-(3-(5-bromo-3-((3-(4-fluorobenzyl)-2,4-dioxothiazolidin-5-ylidene)methyl)-1H-indol-1-y-l)propyl)-3-(4-hydroxyphenyl)propenamide* ***(Tb5)****.* Yellow solid. Yields: 79% (153 mg, 0.24 mmol). ^1^H NMR (400 MHz, DMSO-*d6*) δ 8.77 (t, *J* = 5.5 Hz, 1H), 8.33 (d, *J* = 5.2 Hz, 2H), 8.19 (d, *J* = 3.1 Hz, 2H), 7.96 (s, 1H), 7.68 (td, *J* = 8.8, 5.0 Hz, 1H), 7.60 (d, *J* = 8.6 Hz, 1H), 7.43 – 7.34 (m, 3H), 7.17 (t, *J* = 8.6 Hz, 2H), 7.04 (d, *J* = 8.0 Hz, 2H), 6.69 (d, *J* = 8.1 Hz, 2H), 4.81 (s, 2H), 4.25 (t, *J* = 6.8 Hz, 2H), 4.20 (t, *J* = 6.6 Hz, 1H), 3.04 (p, *J* = 6.5 Hz, 2H), 2.93 (d, *J* = 7.2 Hz, 2H), 1.85 (dp, *J* = 14.5, 7.0 Hz, 2H). **^13^**C NMR (101 MHz, DMSO-*d6*) δ 168.54, 167.60, 165.76, 163.33, 160.91, 157.02, 135.18, 133.27, 132.53, 130.88, 130.44, 130.35, 129.62, 129.13, 126.30, 125.90, 125.42, 121.97, 116.04, 115.82, 115.75, 114.98, 114.84, 113.59, 109.81, 54.37, 49.03, 44.59, 36.64, 36.35, 29.62. C_31_H_28_N_4_O_4_BrFS, HRMS calculated for m/z [M+Na]^+^: 673.0896 (calculated), 673.0900 (found).

*(S,Z)-2-amino-N-(3-(5-bromo-3-((3-(4-fluorobenzyl)-2,4-dioxothiazolidin-5-ylidene)methyl)-1H-indol-1-yl)propyl)-4-(methylthio)butanamide* ***(Tb6)****.* Yellow solid. Yields: 82% (127 mg, 0.21 mmol). ^1^H NMR (400 MHz, DMSO-*d6*) δ 8.13 (q, *J* = 4.5, 4.0 Hz, 3H), 7.97 – 7.90 (m, 1H), 7.56 (dd, *J* = 8.9, 3.0 Hz, 1H), 7.38 – 7.30 (m, 3H), 7.13 (dd, *J* = 10.7, 7.8 Hz, 2H), 4.77 (d, *J* = 8.1 Hz, 2H), 4.31 (d, *J* = 7.8 Hz, 2H), 3.25 – 3.16 (m, 3H), 3.04 (d, *J* = 7.4 Hz, 2H), 2.49 – 2.44 (m, 2H), 2.00 – 1.96 (m, 2H), 1.92 – 1.77 (m, 3H). **^13^**C NMR (101 MHz, DMSO-*d6*) δ 175.40, 167.57, 165.74, 160.92, 135.22, 133.35, 132.51, 130.46, 130.37, 129.64, 126.25, 125.85, 121.94, 116.03, 115.82, 114.84, 113.50, 109.79, 54.47, 44.85, 44.24, 36.25, 35.00, 30.37, 30.04, 15.08. C_27_H_28_N_4_O_3_BrFS_2_, HRMS calculated for m/z [M+Na]^+^: 641.0668 (calculated), 641.0672 (found).

*(2S,3S)-2-amino-N-(3-(5-bromo-3-((Z)-(3-(4-fluorobenzyl)-2,4-dioxothiazolidin-5-ylide-ne)methyl)-1H-in-dol-1-yl)propyl)-3-methylpentanamide* ***(Tb7)***. Yellow solid. Yields: 84% (142 mg, 0.24 mmol). ^1^H NMR (400 MHz, DMSO-*d6*) δ 8.63 (t, *J* = 5.6 Hz, 1H), 8.25 (d, *J* = 1.9 Hz, 1H), 8.23 (s, 1H), 8.19 (s, 2H), 7.96 (s, 1H), 7.64 (d, *J* = 8.8 Hz, 1H), 7.44 (dd, *J* = 8.7, 1.9 Hz, 1H), 7.41 – 7.36 (m, 2H), 7.23 – 7.17 (m, 2H), 4.83 (s, 2H), 4.39 (t, *J* = 6.9 Hz, 2H), 3.63 – 3.56 (m, 1H), 3.15 (dt, *J* = 13.2, 6.6 Hz, 2H), 1.96 (h, *J* = 6.9 Hz, 2H), 1.82 (dtd, *J* = 10.0, 6.6, 3.7 Hz, 1H), 1.50 (dtt, *J* = 14.7, 7.3, 3.7 Hz, 1H), 1.12 (ddd, *J* = 13.8, 9.3, 7.1 Hz, 1H), 0.92 – 0.85 (m, 6H). **^13^**C NMR (100 MHz, DMSO-*d6*) δ 168.32, 167.51, 165.73, 160.92, 135.24, 133.15, 132.55, 132.51, 130.47, 130.39, 129.64, 126.33, 125.89, 122.12, 116.05, 115.83, 115.06, 114.87, 113.46, 109.97, 57.15, 44.74, 44.27, 36.56, 36.44, 29.94, 24.65, 15.03, 11.55. C_28_H_30_N_4_O_3_BrFS, HRMS calculated for m/z [M+Na]^+^: 623.1104 (calculated), 623.1108 (found).

*(S,Z)-2-amino-N-(3-(3-((3-(4-fluorobenzyl)-2,4-dioxothiazolidin-5-ylidene)methyl)-5-methoxy-1H-indol-1-yl)propyl)-3-(4-hydroxyphenyl)propenamide* ***(Tb8).*** Yellow solid. Yields: 71% (176 mg, 0.29 mmol). ^1^H NMR (400 MHz, DMSO-*d6*) δ 9.27 (s, 1H), 8.24 (s, 1H), 8.21 – 8.16 (m, 1H), 7.87 (s, 1H), 7.52 – 7.48 (m, 2H), 7.41 – 7.35 (m, 2H), 7.22 – 7.16 (m, 2H), 7.04 – 7.00 (m, 2H), 6.93 (dd, *J* = 8.8, 2.4 Hz, 1H), 6.70 – 6.66 (m, 2H), 4.83 (s, 2H), 4.23 (t, *J* = 6.8 Hz, 2H), 3.84 (s, 3H), 3.49 (t, *J* = 6.7 Hz, 1H), 3.09 – 3.02 (m, 2H), 2.85 (dd, *J* = 13.5, 5.9 Hz, 1H), 2.63 (dd, *J* = 13.5, 7.6 Hz, 1H), 1.88 (p, *J* = 7.4 Hz, 2H). **^13^**C NMR (101 MHz, DMSO-*d6*) δ 173.28, 167.71, 165.85, 160.91, 156.43, 155.85, 132.63, 132.54, 131.35, 130.68, 130.42, 130.34, 128.87, 128.10, 126.76, 116.04, 115.83, 115.51, 113.90, 113.06, 112.37, 110.02, 101.10, 56.34, 56.03, 44.69, 44.18, 36.26, 29.97. C_32_H_31_N_4_O_5_FS, HRMS calculated for m/z [M+Na]^+^: 625.1897 (calculated), 625.1900 (found).

*(S,Z)-N-(3-(3-((3-(4-fluorobenzyl)-2,4-dioxothiazolidin-5-ylidene)methyl)-5-methoxy-1H-indol-1-yl)propy-l)pyrrolidine-2-carboxamide1* ***(Tb9).*** Yellow solid. Yields: 76% (154 mg, 0.29 mmol). ^1^H NMR (400 MHz, DMSO-*d6*) δ 8.60 – 8.52 (m, 1H), 8.21 (s, 1H), 7.87 (s, 1H), 7.53 (d, *J* = 8.9 Hz, 1H), 7.46 (d, *J* = 2.4 Hz, 1H), 7.39 – 7.32 (m, 2H), 7.17 (t, *J* = 8.8 Hz, 2H), 6.90 (dd, *J* = 8.9, 2.3 Hz, 1H), 4.80 (s, 2H), 4.35 (t, *J* = 7.0 Hz, 2H), 4.21 (d, *J* = 7.0 Hz, 1H), 3.82 (s, 3H), 3.25 – 3.19 (m, 2H), 3.12 (d, *J* = 6.6 Hz, 2H), 2.36 – 2.27 (m, 1H), 1.96 (q, *J* = 6.9 Hz, 2H), 1.89 – 1.84 (m, 4H). ^13^C NMR (101 MHz, DMSO-*d6*) δ 168.62, 167.67, 165.84, 160.90, 155.83, 132.59, 132.45, 131.36, 130.42, 130.33, 128.82, 126.68, 118.57, 116.00, 115.79, 115.63, 113.87, 113.07, 112.34, 110.07, 101.02, 59.37, 55.95, 48.98, 45.87, 44.64, 44.14, 36.77, 30.09, 29.76, 24.02. C_28_H_29_N_4_O_4_FS, HRMS calculated for m/z [M+H]^+^: 537.1972 (calculated), 537.1974 (found).

*(2E,5Z)-5-((1-(3-aminopropyl)-1H-indol-3-yl)methylene)-3-(4-fluorobenzyl)-2-((4-morpholino-phenyl)im-ino)thiazolidin-4-one* ***(Tc1)****.* Yellow solid. Yields: 81% (128 mg, 0.22 mmol). ^1^H NMR (400 MHz, DMSO-*d6*) δ 7.95 (s, 1H), 7.82 (d, *J* = 7.9 Hz, 1H), 7.76 (s, 2H), 7.66 (s, 1H), 7.57 (d, *J* = 8.2 Hz, 1H), 7.43 – 7.38 (m, 2H), 7.27 – 7.21 (m, 1H), 7.19 – 7.10 (m, 3H), 6.89 (q, *J* = 9.0 Hz, 4H), 4.99 (s, 2H), 4.32 (t, *J* = 7.1 Hz, 2H), 3.68 (dd, *J* = 6.1, 3.5 Hz, 4H), 3.04 (t, *J* = 4.8 Hz, 4H), 2.68 (t, *J* = 7.6 Hz, 2H), 1.95 (p, *J* = 7.4 Hz, 2H). ^13^C NMR (100 MHz, DMSO-*d6*) δ 165.90, 162.81, 160.38, 148.29, 148.22, 139.49, 135.89, 132.77, 130.63, 130.13, 130.05, 127.44, 123.24, 122.20, 121.86, 121.37, 118.72, 115.93, 115.45, 115.24, 114.33, 110.86, 109.99, 66.19, 48.60, 45.03, 43.38, 36.51, 27.88. C_32_H_32_N_5_O_2_FS, HRMS calculated for m/z [M+H]^+^: 570.2294 (calculated), 570.2326 (found).

*(S)-2-amino-N-(3-(3-((Z)-((E)-3-(4-fluorobenzyl)-2-((4-morpholinophenyl)imino)-4oxothiazoli-din-5-ylide-ne)methyl)-1H-indol-1-yl)propyl)-4-(methylthio)butanamide* ***(Tc2)*.** Yellow solid. Yields: 80% (169 mg, 0.24 mmol). ^1^H NMR (400 MHz, DMSO-*d6*) δ 8.70 (t, *J* = 5.5 Hz, 1H), 8.32 (d, *J* = 5.2 Hz, 2H), 8.01 (s, 1H), 7.86 (d, *J* = 7.9 Hz, 1H), 7.73 (s, 1H), 7.61 (d, *J* = 8.2 Hz, 1H), 7.47 (dd, *J* = 8.6, 5.6 Hz, 2H), 7.29 (t, *J* = 7.6 Hz, 1H), 7.24 – 7.15 (m, 3H), 7.02 – 6.94 (m, 4H), 5.05 (s, 2H), 4.34 (t, *J* = 7.5 Hz, 2H), 3.86 – 3.80 (m, 1H), 3.75 (dd, *J* = 6.1, 3.5 Hz, 4H), 3.15 – 3.09 (m, 6H), 2.48 – 2.43 (m, 2H), 2.01 (s, 3H), 1.99 – 1.88 (m, 4H). **^13^**C NMR (101 MHz, DMSO-*d6*) δ 168.52, 166.31, 163.23, 160.81, 148.63, 148.42, 140.10, 136.33, 133.20, 131.20, 130.56, 130.47, 127.85, 123.57, 122.76, 122.36, 121.69, 119.08, 116.56, 115.85, 115.64, 114.54, 111.29, 110.26, 66.54, 52.14, 49.20, 45.42, 44.51, 36.84, 31.22, 29.95, 28.76, 14.87. C_37_H_41_N_6_O_3_FS_2_, HRMS calculated for m/z [M+H]^+^: 701.2699 (calculated), 701.2745 (found).

*(S)-2-amino-N-(3-(3-((Z)-((E)-3-(4-fluorobenzyl)-2-((4-morpholinophenyl)imino)-4-oxothiazoli-din-5-ylid-ene)methyl)-1H-indol-1-yl)propyl)-3-methylbutanamide* ***(Tc3)****.* Yellow solid. Yields: 74% (202 mg, 0.30 mmol). ^1^H NMR (400 MHz, DMSO-*d6*) δ 8.02 (s, 1H), 7.99 – 7.91 (m, 3H), 7.62 (d, *J* = 8.2 Hz, 1H), 7.32 (td, *J* = 7.0, 5.6, 2.5 Hz, 3H), 7.27 – 7.23 (m, 3H), 7.15 (t, *J* = 8.9 Hz, 2H), 7.02 (d, *J* = 8.7 Hz, 2H), 4.61 (s, 2H), 4.36 (t, *J* = 6.9 Hz, 2H), 3.75 (t, *J* = 4.7 Hz, 4H), 3.16 (q, *J* = 6.9, 5.7 Hz, 6H), 2.94 (d, *J* = 5.3 Hz, 1H), 1.97 (p, *J* = 7.0 Hz, 2H), 1.86 (p, *J* = 6.6 Hz, 1H), 0.87 (d, *J* = 6.8 Hz, 3H), 0.80 (d, *J* = 6.8 Hz, 3H). ^13^C NMR (101 MHz, DMSO-*d6*) δ 168.25, 166.31, 163.24, 160.81, 148.61, 148.41, 140.11, 136.33, 133.20, 131.16, 130.55, 130.47, 127.85, 123.59, 122.75, 122.36, 121.70, 119.08, 116.58, 115.86, 115.64, 114.57, 111.32, 110.28, 66.53, 58.06, 49.23, 45.42, 44.55, 36.68, 30.08, 18.74, 18.40. C_37_H_41_N_6_O_3_FS, HRMS calculated for m/z [M+H]^+^: 669.2978 (calculated), 669.3014 (found).

*(S)-N-(3-(3-((Z)-((E)-3-(4-fluorobenzyl)-2-((4-morpholinophenyl)imino)-4-oxothiazolidin-5-ylidene)methy-l)-1H-indol-1-yl)propyl)pyrrolidine-2-carboxamide* ***(Tc4)****.* Yellow solid. Yields: 78% (185 mg, 0.28 mmol). ^1^H NMR (400 MHz, DMSO-*d6*) δ 8.63 (t, *J* = 5.5 Hz, 1H), 8.02 (s, 1H), 7.88 (d, *J* = 7.9 Hz, 1H), 7.73 (s, 1H), 7.60 (d, *J* = 8.2 Hz, 1H), 7.48 (dd, *J* = 8.4, 5.4 Hz, 2H), 7.30 (t, *J* = 7.6 Hz, 1H), 7.25 – 7.17 (m, 3H), 6.98 (q, *J* = 8.8 Hz, 4H), 5.07 (s, 2H), 4.34 (t, *J* = 7.1 Hz, 2H), 4.13 (t, *J* = 6.9 Hz, 1H), 3.75 (d, *J* = 8.1 Hz, 4H), 3.22 – 3.16 (m, 2H), 3.12 (t, *J* = 4.8 Hz, 6H), 2.25 (tt, *J* = 12.5, 6.1 Hz, 1H), 1.97 – 1.77 (m, 6H). **^13^**C NMR (101 MHz, DMSO-*d6*) δ 168.51, 166.32, 163.23, 160.81, 148.64, 148.61, 139.92, 136.33, 133.21, 131.24, 130.56, 130.48, 127.85, 123.60, 122.76, 122.34, 121.71, 119.10, 116.41, 115.88, 115.66, 114.55, 111.27, 110.25, 66.59, 59.47, 49.08, 45.99, 45.43, 44.40, 36.97, 29.95, 29.89, 23.99. C_37_H_39_N_6_O_3_FS, HRMS calculated for m/z [M+H]^+^: 667.2822 (calculated), 667.2847 (found).

*(2S,3S)-2-amino-N-(3-(3-((Z)-((Z)-3-(4-fluorobenzyl)-2-((4-morpholinophenyl)imino)-4-oxothiazolidin-5-ylidene)methyl)-1H-indol-1-yl)propyl)-3-methylpentanamide* ***(Tc5)****.*Yellow solid. Yields: 78% (154 mg, 0.23 mmol). ^1^H NMR (400 MHz, DMSO-*d*_6_) δ 8.01 (s, 1H), 7.99 – 7.90 (m, 3H), 7.62 (d, *J* = 8.2 Hz, 1H), 7.31 (dd, *J* = 8.6, 5.2 Hz, 3H), 7.27 – 7.21 (m, 3H), 7.18 – 7.11 (m, 2H), 7.02 (d, *J* = 8.5 Hz, 2H), 4.60 (s, 2H), 4.36 (t, *J* = 6.9 Hz, 2H), 3.74 (t, *J* = 4.8 Hz, 4H), 3.14 (dt, *J* = 16.7, 5.8 Hz, 6H), 2.96 (d, *J* = 5.6 Hz, 1H), 1.95 (p, *J* = 7.2 Hz, 2H), 1.59 (dq, *J* = 10.4, 5.2 Hz, 1H), 1.43 (ddd, *J* = 12.2, 7.5, 4.1 Hz, 1H), 1.12 – 1.01 (m, 1H), 0.85 – 0.77 (m, 6H). ^13^C NMR (101 MHz, DMSO-*d*_6_) δ 175.35, 166.44, 160.43, 151.11, 150.80, 136.31, 135.70, 131.48, 129.63, 129.55, 129.42, 127.91, 127.11, 123.55, 121.87, 121.62, 119.20, 115.65, 115.43, 115.26, 114.79, 111.21, 110.46, 66.54, 59.98, 55.38, 48.51, 44.54, 38.86, 36.22, 30.33, 24.33, 16.35, 12.01. C_38_H_43_N_6_O_3_FS, HRMS calculated for m/z [M+H]^+^: 683.3180 (calculated), 683.3182 (found).

*(S)-2-amino-N-(3-(3-((Z)-((E)-3-(4-fluorobenzyl)-4-oxo-2-(phenylimino)thiazolidin-5-ylidene)methyl)-1H--indol-1-yl)propyl)-3-methylbutanamide* ***(Tc6)****.* Yellow solid. Yields: 74% (175 mg, 0.30 mmol). ^1^H NMR (400 MHz, DMSO-*d6*) δ 8.09 (s, 1H), 8.04 (s, 1H), 8.00 (s, 1H), 7.92 (d, *J* = 7.7 Hz, 1H), 7.62 (d, *J* = 8.0 Hz, 1H), 7.50 (d, *J* = 7.0 Hz, 2H), 7.44 (d, *J* = 7.1 Hz, 3H), 7.31 (s, 3H), 7.26 – 7.21 (m, 1H), 7.14 (t, *J* = 8.1 Hz, 2H), 4.60 (s, 2H), 4.37 (s, 2H), 3.16 – 3.13 (m, 2H), 3.02 (d, *J* = 4.6 Hz, 1H), 1.99 – 1.94 (m, 2H), 1.90 (d, *J* = 6.3 Hz, 1H), 0.88 (d, *J* = 6.4 Hz, 3H), 0.82 (d, *J* = 6.4 Hz, 3H). ^13^C NMR (101 MHz, DMSO-*d6*) δ 174.46, 166.24, 162.84, 160.43, 150.56, 143.87, 136.32, 136.14, 135.62, 131.60, 129.57, 129.50, 129.38, 129.07, 128.81, 127.92, 123.57, 122.14, 121.65, 119.20, 115.64, 115.43, 114.68, 111.24, 110.45, 60.39, 55.33, 44.54, 36.28, 31.86, 30.30, 19.92, 17.81. C_33_H_34_N_5_O_2_FS, HRMS calculated for m/z [M+Na]^+^: 606.2315 (calculated), 606.2319 (found).

*(S)-2-amino-N-(3-(3-((Z)-((E)-3-(4-fluorobenzyl)-2-((4-fluorophenyl)imino)-4-oxothiazo-lidin-5-ylidene)m-ethyl)-1H-indol-1-yl)propyl)-3-methylbutanamide* ***(Tc7)****.* Yellow solid. Yields: 78% (154 mg, 0.26 mmol). ^1^H NMR (400 MHz, DMSO-*d6*) δ 8.05 (d, *J* = 12.5 Hz, 2H), 8.00 (s, 1H), 7.94 (d, *J* = 7.9 Hz, 1H), 7.63 (d, *J* = 8.2 Hz, 1H), 7.52 (dd, *J* = 8.5, 5.0 Hz, 2H), 7.38 – 7.29 (m, 6H), 7.24 (t, *J* = 7.5 Hz, 1H), 7.15 (t, *J* = 8.6 Hz, 2H), 4.61 (s, 2H), 4.37 (t, *J* = 6.9 Hz, 2H), 3.14 (q, *J* = 6.7 Hz, 2H), 3.01 (d, *J* = 5.3 Hz, 1H), 1.97 (q, *J* = 6.9 Hz, 2H), 1.93 – 1.85 (m, 1H), 0.87 (d, *J* = 6.9 Hz, 3H), 0.81 (d, *J* = 6.8 Hz, 3H). ^13^C NMR (101 MHz, DMSO-*d6*) δ 174.42, 166.21, 150.60, 136.32, 135.58, 132.30, 131.61, 131.31, 131.22, 129.58, 129.50, 127.89, 123.59, 122.19, 121.68, 119.20, 116.39, 116.17, 115.65, 115.44, 114.62, 111.25, 110.41, 60.36, 55.27, 44.54, 36.27, 31.83, 30.29, 19.90, 17.79. C_33_H_33_N_5_O_2_F_2_S, HRMS calculated for m/z [M+H]^+^: 602.2401 (calculated), 602.2404 (found).

*(S)-2-amino-N-(3-(3-((Z)-((E)-2-((4-chlorophenyl)imino)-3-(4-fluorobenzyl)-4-oxothiazolidin-5-ylidene)m-ethyl)-1H-indol-1-yl)propyl)-3-methylbutanamide* ***(Tc8).*** Yellow solid. Yields: 84% (175 mg, 0.28 mmol). ^1^H NMR (400 MHz, DMSO-*d6*) δ 8.05 (s, 1H), 8.00 (d, *J* = 8.1 Hz, 2H), 7.94 (d, *J* = 8.0 Hz, 1H), 7.61 (dd, *J* = 16.2, 8.2 Hz, 3H), 7.51 (d, *J* = 8.3 Hz, 2H), 7.32 (t, *J* = 7.1 Hz, 3H), 7.24 (t, *J* = 7.5 Hz, 1H), 7.15 (t, *J* = 8.7 Hz, 2H), 4.61 (s, 2H), 4.38 (d, *J* = 6.8 Hz, 2H), 3.14 (q, *J* = 6.7 Hz, 2H), 2.94 (d, *J* = 5.1 Hz, 1H), 1.99 – 1.93 (m, 2H), 1.88 (t, *J* = 6.9 Hz, 1H), 0.87 (d, *J* = 6.8 Hz, 3H), 0.80 (d, *J* = 6.8 Hz, 3H). ^13^C NMR (101 MHz, DMSO-*d6*) δ 175.22, 166.04, 162.85, 160.45, 150.38, 136.35, 135.55, 134.95, 133.35, 131.66, 130.98, 129.59, 129.52, 129.41, 127.91, 123.60, 122.33, 121.69, 119.21, 115.65, 115.44, 114.56, 111.25, 110.42, 60.68, 55.30, 44.55, 36.24, 32.04, 30.33, 20.04, 17.70. C_33_H_33_N_5_O_2_FSCl, HRMS calculated for m/z [M+H]^+^: 618.2106 (calculated), 618.2109 (found).

*(S)-2-amino-N-(3-(3-((Z)-((E)-3-(4-fluorobenzyl)-4-oxo-2-((4-(trifluoromethyl)phenyl)im-ino)t-hia-zolidin-e-5-ylidene)methyl)-1H-indol-1-yl)propyl)-3-methylbutanamide* ***(Tc9)***. Yellow solid. Yields: 71% (136 mg, 0.21 mmol). ^1^H NMR (400 MHz, DMSO-*d6*) δ 8.08 (s, 1H), 7.90 (d, *J* = 8.2 Hz, 2H), 7.81 – 7.76 (m, 3H), 7.59 (d, *J* = 8.2 Hz, 1H), 7.50 (dd, *J* = 8.4, 5.5 Hz, 2H), 7.30 (t, *J* = 7.7 Hz, 1H), 7.22 (q, *J* = 8.5 Hz, 5H), 5.09 (s, 2H), 4.29 (t, *J* = 7.0 Hz, 2H), 3.04 (q, *J* = 6.8 Hz, 2H), 2.87 (d, *J* = 5.3 Hz, 1H), 1.87 (q, *J* = 7.1 Hz, 2H), 1.79 (dt, *J* = 13.1, 6.8 Hz, 1H), 0.83 (d, *J* = 6.7 Hz, 3H), 0.75 (d, *J* = 6.7 Hz, 3H). ^13^C NMR (101 MHz, DMSO-*d6*) δ 175.11, 166.26, 151.74, 151.23, 136.32, 132.92, 131.71, 130.56, 130.47, 127.93, 127.29, 123.89, 123.63, 122.37, 121.80, 119.10, 115.92, 115.71, 113.49, 111.33, 109.97, 60.66, 45.50, 44.57, 36.15, 32.01, 30.31, 19.95, 17.65. C_34_H_33_N_5_O_2_F_4_S, HRMS calculated for m/z [M+H]^+^: 652.2369 (calculated), 652.2373 (found).

*(S)-2-amino-N-(3-(3-((Z)-((E)-3-(4-fluorobenzyl)-2-((4-methoxyphenyl)imino)-4-oxothiazolidn-5-ylidene)methyl)-1H-indol-1-yl)propyl)-3-methylbutanamide* ***(Tc10)****.* Yellow solid. Yields: 73% (129 mg, 0.21 mmol). ^1^H NMR (400 MHz, DMSO-*d6*) δ 8.03 (d, *J* = 4.1 Hz, 2H), 7.99 (s, 1H), 7.93 (d, *J* = 7.9 Hz, 1H), 7.63 (d, *J* = 8.2 Hz, 1H), 7.36 (s, 1H), 7.33 (s, 1H), 7.33 – 7.29 (m, 3H), 7.24 (t, *J* = 7.5 Hz, 1H), 7.15 (t, *J* = 8.8 Hz, 2H), 7.06 (s, 1H), 7.03 (s, 1H), 4.60 (s, 2H), 4.37 (t, *J* = 6.7 Hz, 2H), 3.80 (s, 3H), 3.14 (q, *J* = 6.4 Hz, 2H), 2.98 (d, *J* = 5.3 Hz, 1H), 2.00 – 1.93 (m, 2H), 1.87 (dt, *J* = 13.1, 6.6 Hz, 1H), 0.87 (d, *J* = 6.8 Hz, 3H), 0.80 (d, *J* = 6.8 Hz, 3H). ^13^C NMR (101 MHz, DMSO-*d6*) δ 174.70, 166.39, 162.80, 160.40, 159.38, 150.76, 136.32, 135.67, 131.52, 130.14, 129.60, 129.52, 128.65, 127.91, 123.56, 121.97, 121.63, 119.20, 115.64, 115.43, 114.75, 114.59, 111.23, 110.45, 60.47, 55.80, 55.32, 44.53, 36.26, 31.90, 30.31, 19.95, 17.76. C_34_H_36_N_5_O_3_FS, HRMS calculated for m/z [M+H]^+^: 614.2601 (calculated), 614.2604 (found).

*(S)-2-amino-N-(3-(3-((Z)-((E)-2-(cyclohexylimino)-3-(4-fluorobenzyl)-4-oxothiazolidin-5-ylidene)methyl)-1H-indol-1-yl)propyl)-3-methylbutanamide* ***(Tc11).*** Yellow solid. Yields: 70% (121 mg, 0.21 mmol). ^1^H NMR (400 MHz, DMSO-*d6*) δ 7.95 (s, 2H), 7.86 (d, *J* = 7.8 Hz, 2H), 7.58 (d, *J* = 8.2 Hz, 1H), 7.41 (dd, *J* = 8.5, 5.6 Hz, 2H), 7.28 (t, *J* = 7.6 Hz, 1H), 7.21 (t, *J* = 7.5 Hz, 1H), 7.14 (t, *J* = 8.8 Hz, 2H), 4.90 (s, 2H), 4.33 (t, *J* = 7.0 Hz, 2H), 3.12 (q, *J* = 6.5 Hz, 2H), 2.94 (d, *J* = 5.3 Hz, 1H), 1.96 – 1.91 (m, 2H), 1.91 – 1.86 (m, 1H), 1.71 (d, *J* = 9.5 Hz, 5H), 1.61 – 1.54 (m, 2H), 1.39 (q, *J* = 13.3, 12.2 Hz, 4H), 0.89 (d, *J* = 6.8 Hz, 3H), 0.82 (d, *J* = 6.8 Hz, 3H). ^13^C NMR (101 MHz, DMSO-*d6*) δ 175.25, 166.31, 163.17, 160.75, 145.59, 136.27, 133.44, 131.37, 130.65, 130.57, 127.91, 123.45, 121.58, 121.51, 119.04, 115.66, 115.45, 114.72, 111.15, 110.32, 61.63, 60.72, 45.04, 44.49, 36.18, 33.84, 32.08, 30.37, 25.67, 24.23, 20.07, 17.69. C_33_H_40_N_5_O_2_FS, HRMS calculated for m/z [M+H]^+^: 590.2965 (calculated), 590.2969 (found).

*(S)-2-amino-N-(3-(3-((Z)-((E)-2-(butylimino)-3-(4-fluorobenzyl)-4-oxothiazolidin-5-ylidene)m-ethyl)-1H-i-ndol-1-yl)propyl)-3-methylbutanamide* ***(Tc12)****.* Yellow solid. Yields: 71% (105 mg, 0.19 mmol). ^1^H NMR (400 MHz, DMSO-*d6*) δ 7.96 (d, *J* = 5.7 Hz, 2H), 7.89 (s, 2H), 7.59 (d, *J* = 8.2 Hz, 1H), 7.37 (dd, *J* = 8.3, 5.8 Hz, 2H), 7.29 (t, *J* = 7.6 Hz, 1H), 7.21 (t, *J* = 7.5 Hz, 1H), 7.15 (t, *J* = 8.8 Hz, 2H), 4.91 (s, 2H), 4.33 (t, *J* = 6.7 Hz, 2H), 3.11 (q, *J* = 6.4 Hz, 2H), 2.92 (d, *J* = 5.2 Hz, 1H), 1.96 – 1.91 (m, 2H), 1.85 (dt, *J* = 13.1, 6.7 Hz, 1H), 1.65 (s, 2H), 1.58 (p, *J* = 6.9 Hz, 2H), 1.31 (h, *J* = 7.3 Hz, 2H), 0.90 (s, 1H), 0.88 (s, 3H), 0.86 (s, 2H), 0.80 (d, *J* = 6.8 Hz, 3H). ^13^C NMR (101 MHz, DMSO-*d6*) δ 175.27, 166.35, 163.14, 160.72, 147.56, 136.26, 133.41, 131.42, 130.40, 130.31, 127.87, 123.49, 121.72, 121.56, 119.10, 115.72, 115.51, 114.70, 111.18, 110.33, 60.69, 52.54, 45.02, 44.49, 36.19, 32.93, 32.08, 30.33, 20.34, 20.07, 17.69, 14.21. C_31_H_38_N_5_O_2_FS, HRMS calculated for m/z [M+H]^+^: 564.2808 (calculated), 564.2812 (found).

# Part C. Copies of Spectra

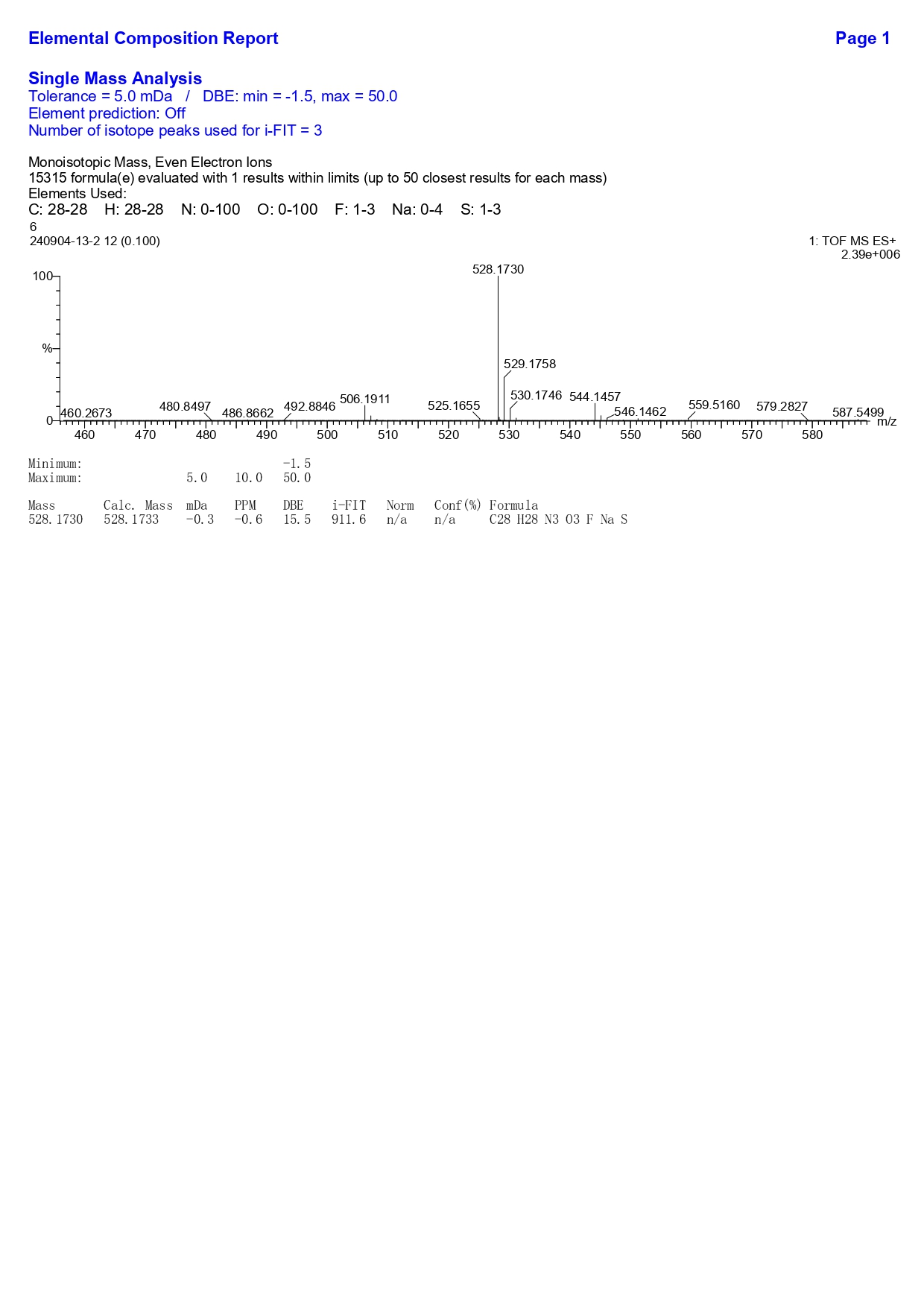

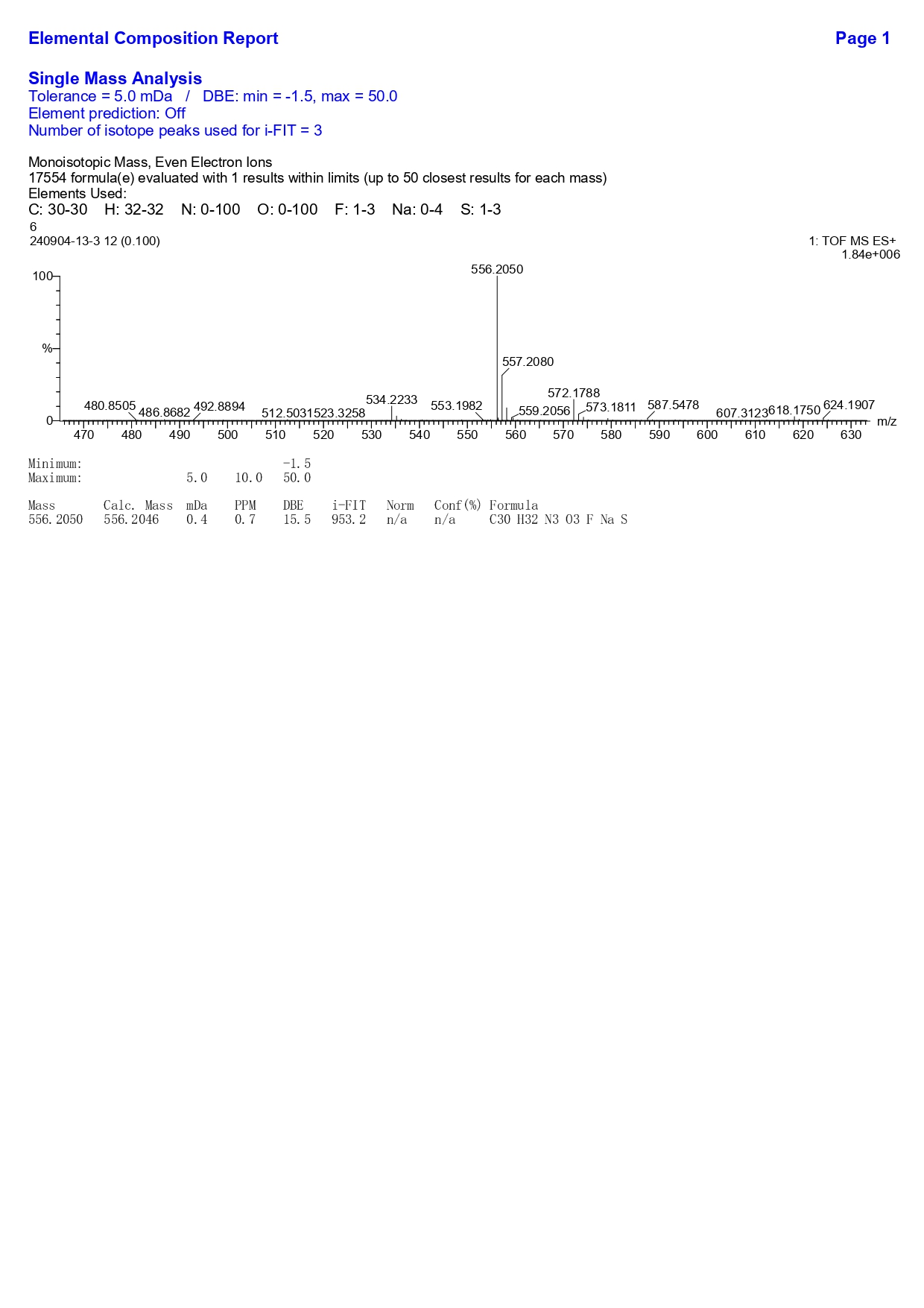

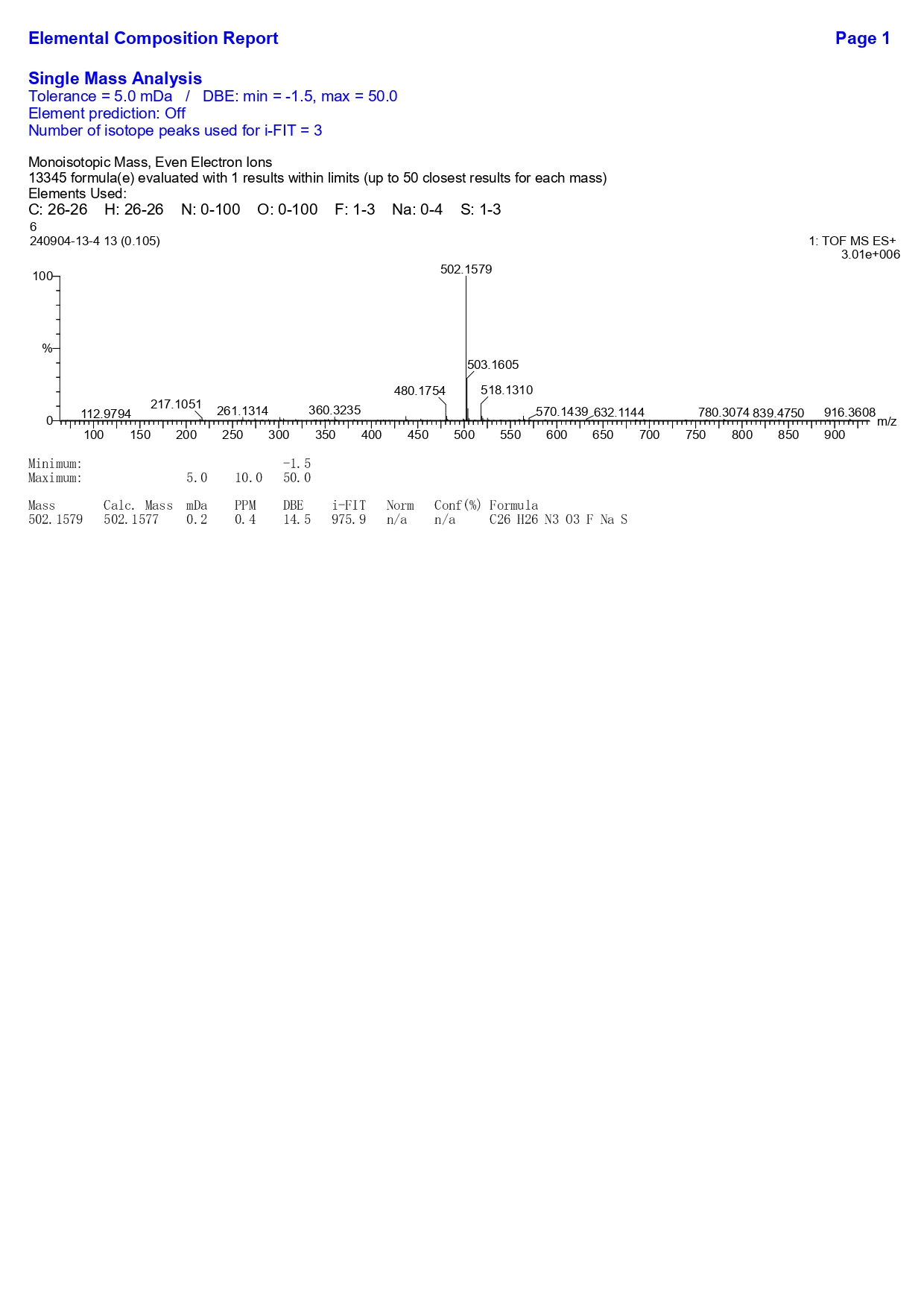

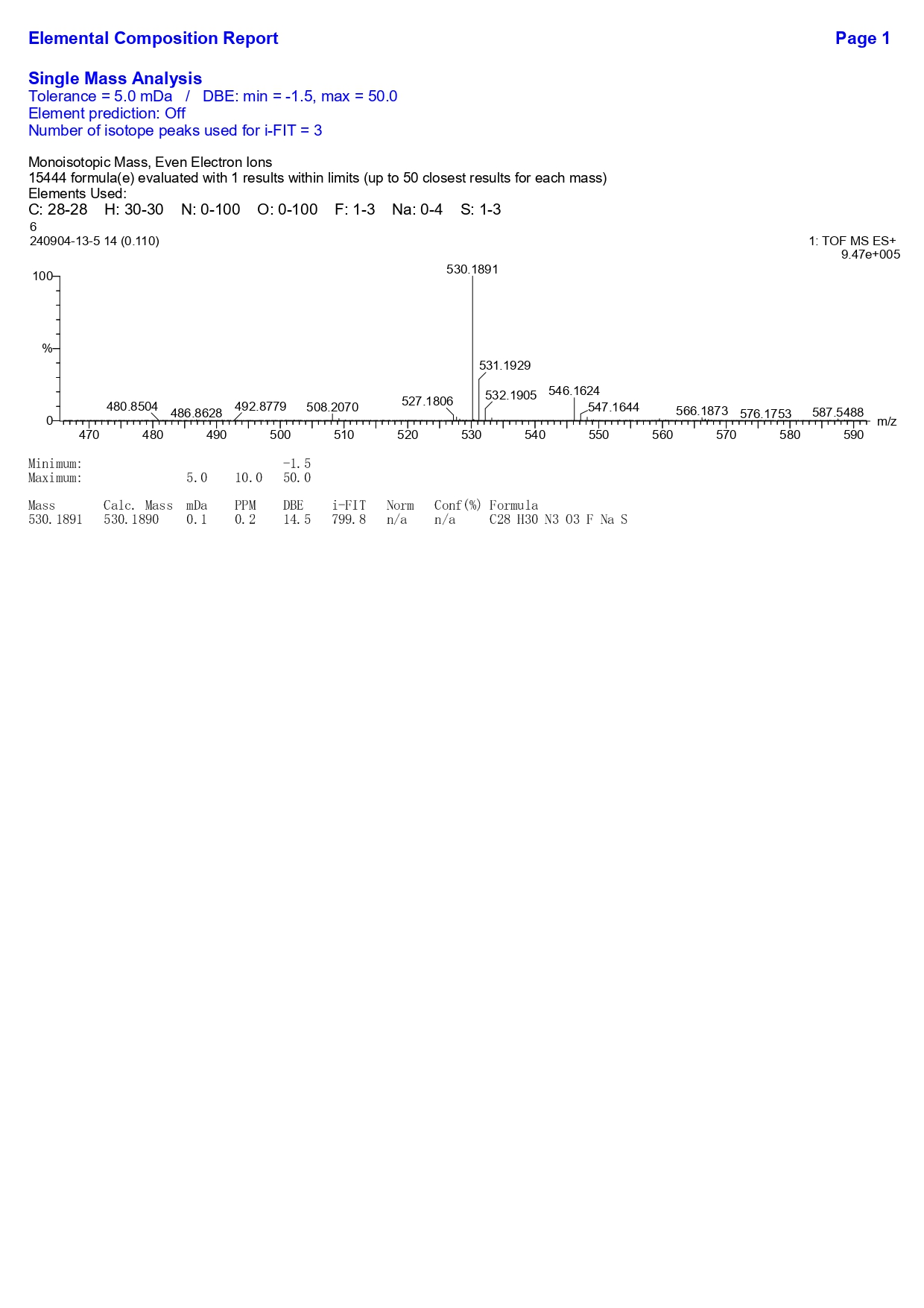

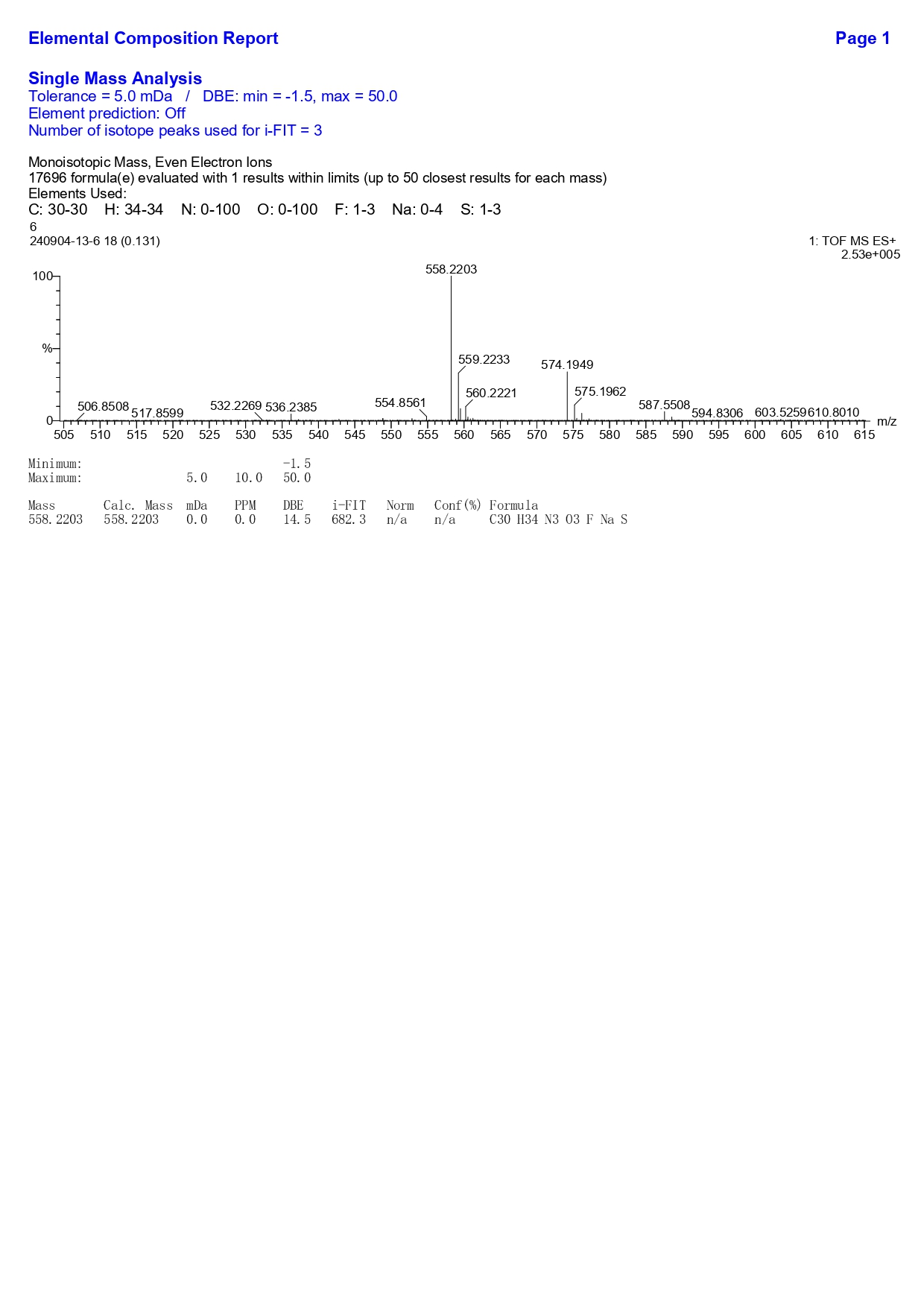

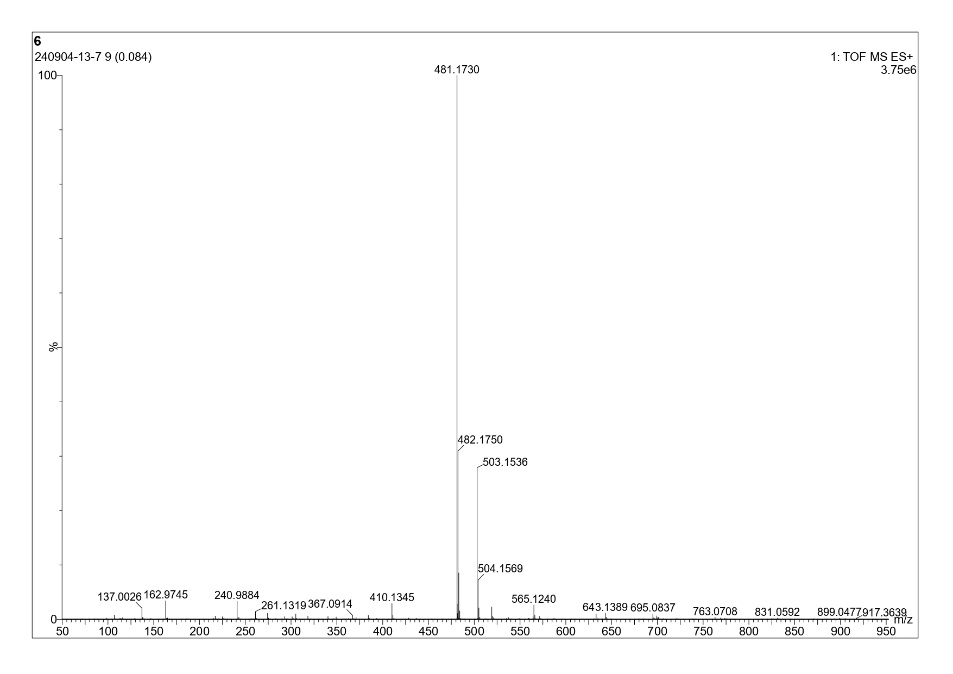

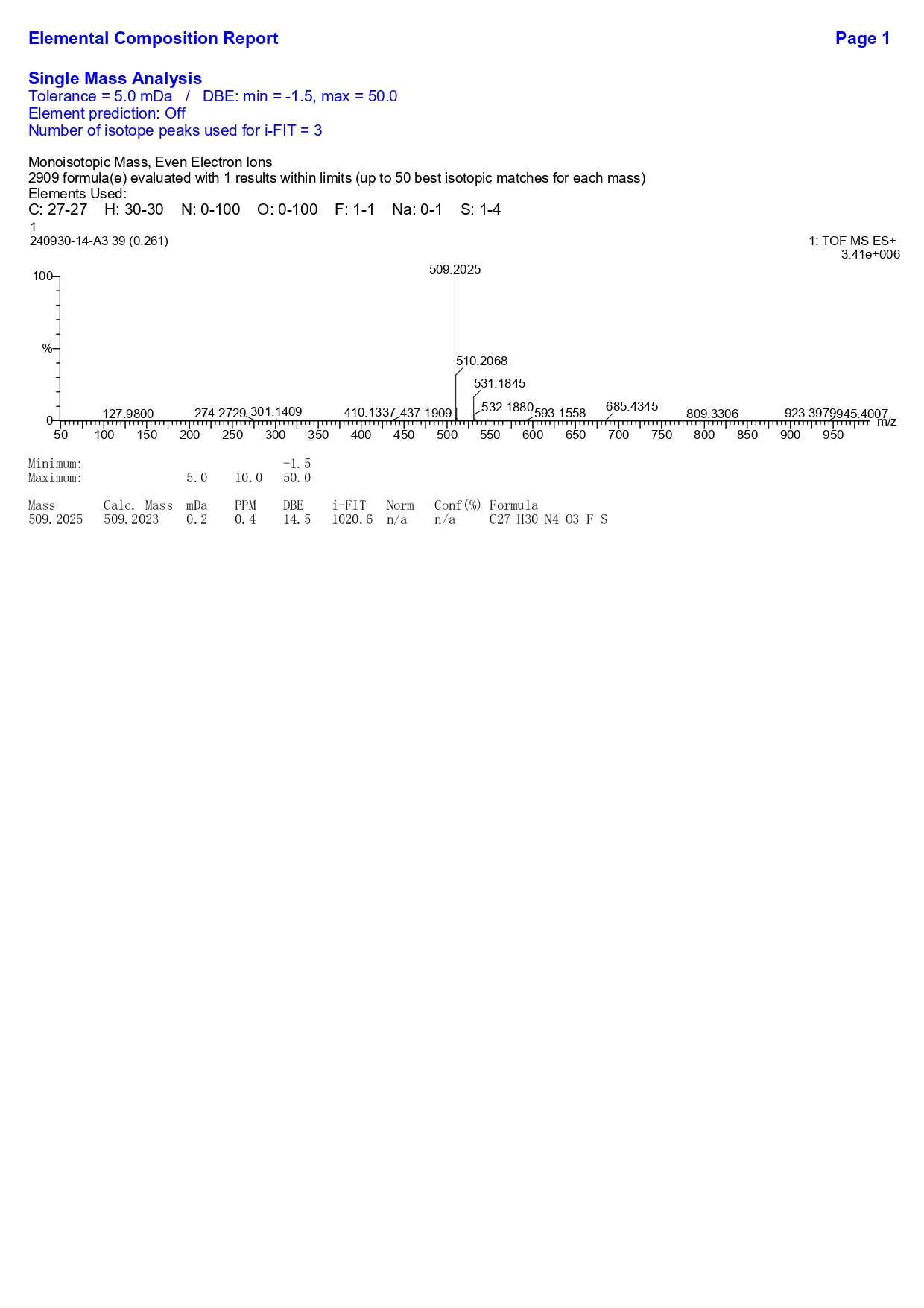

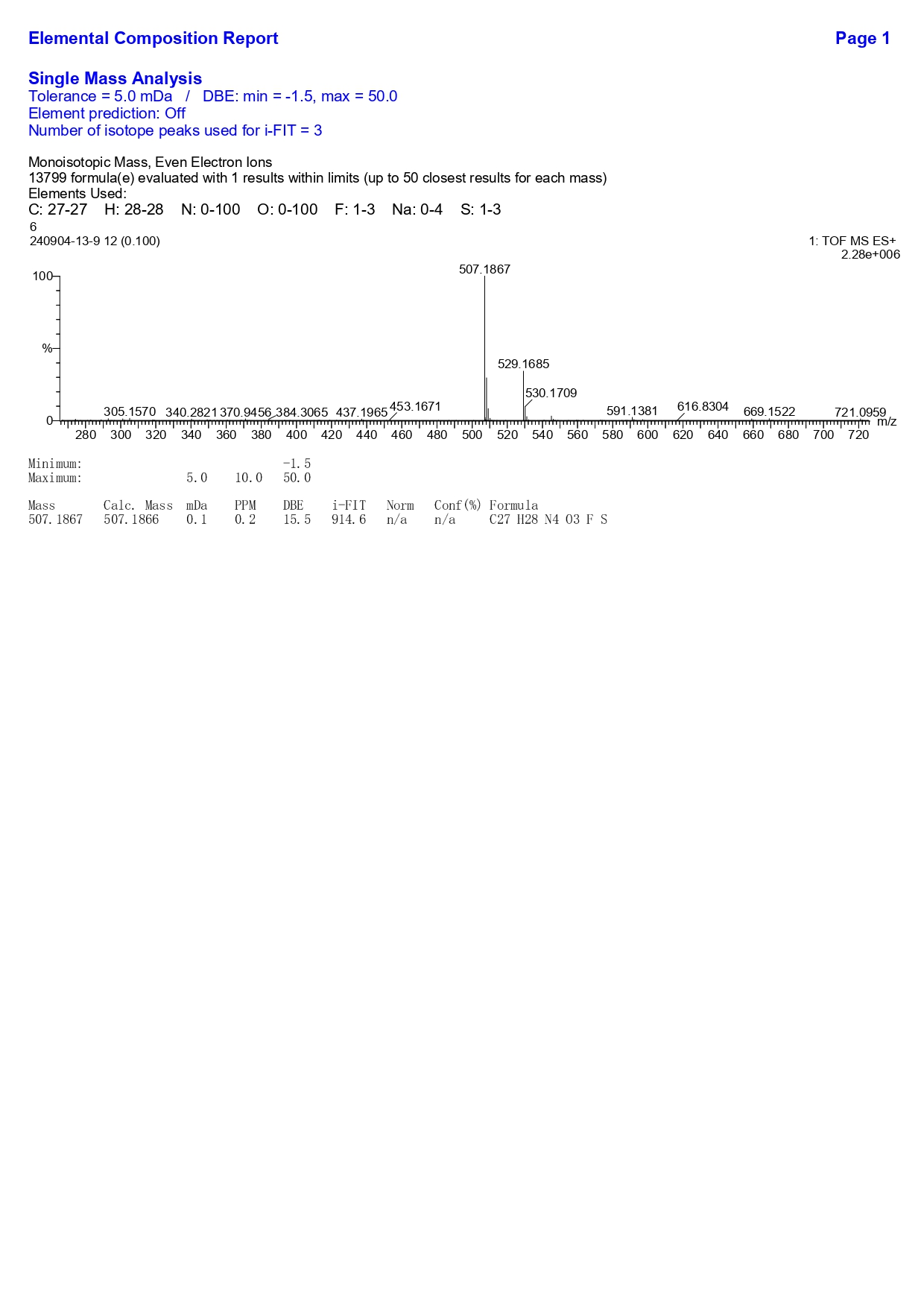

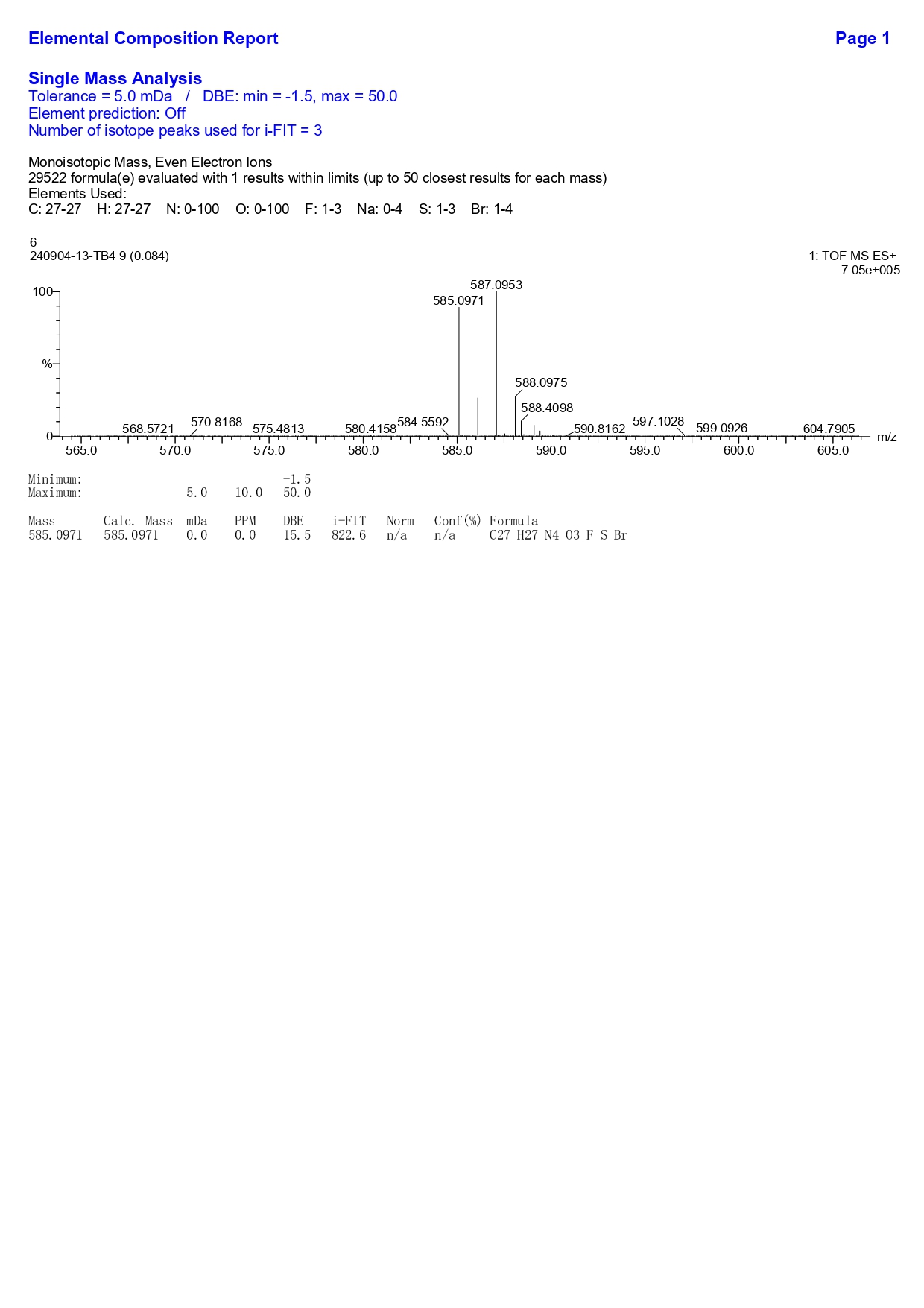

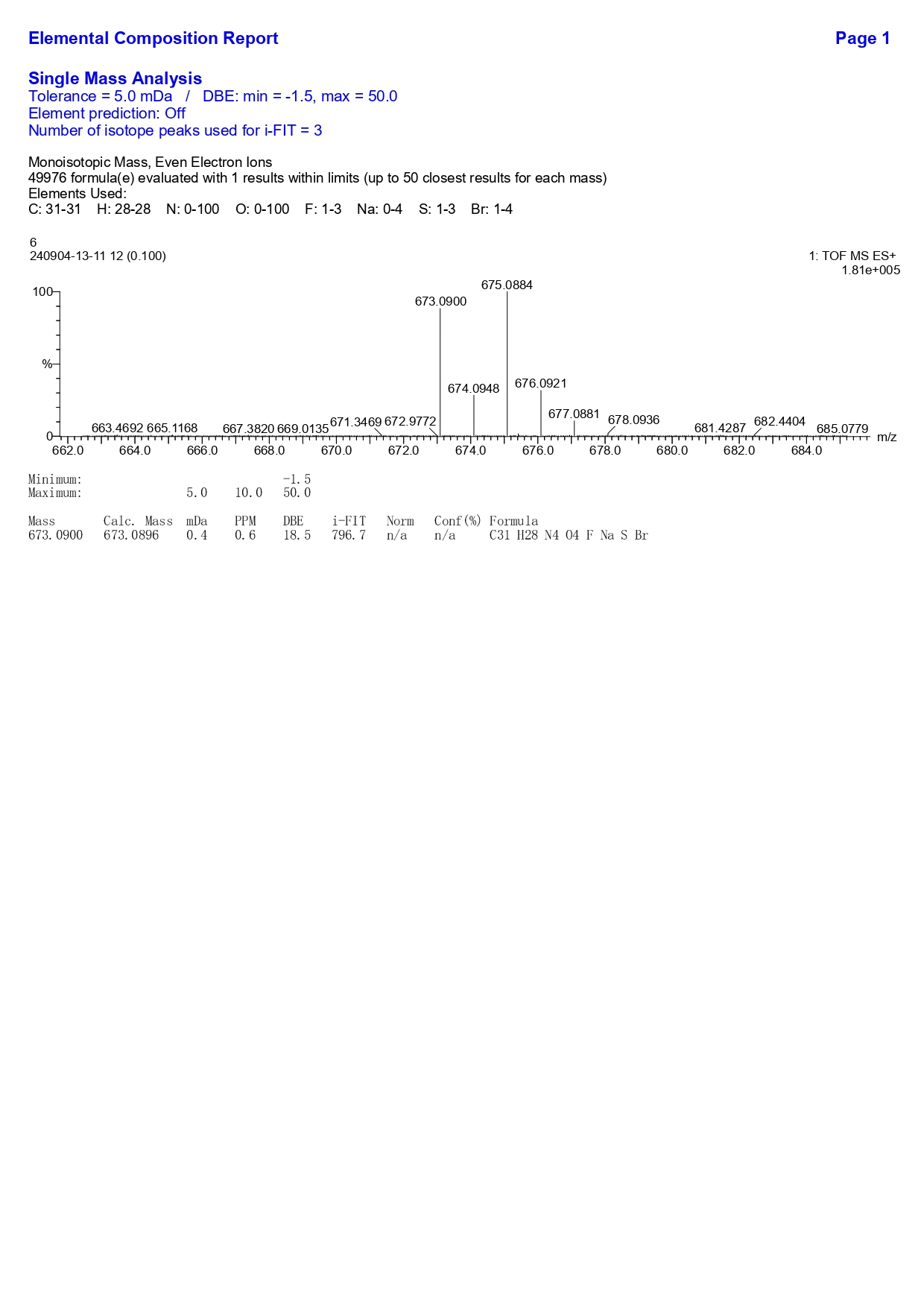

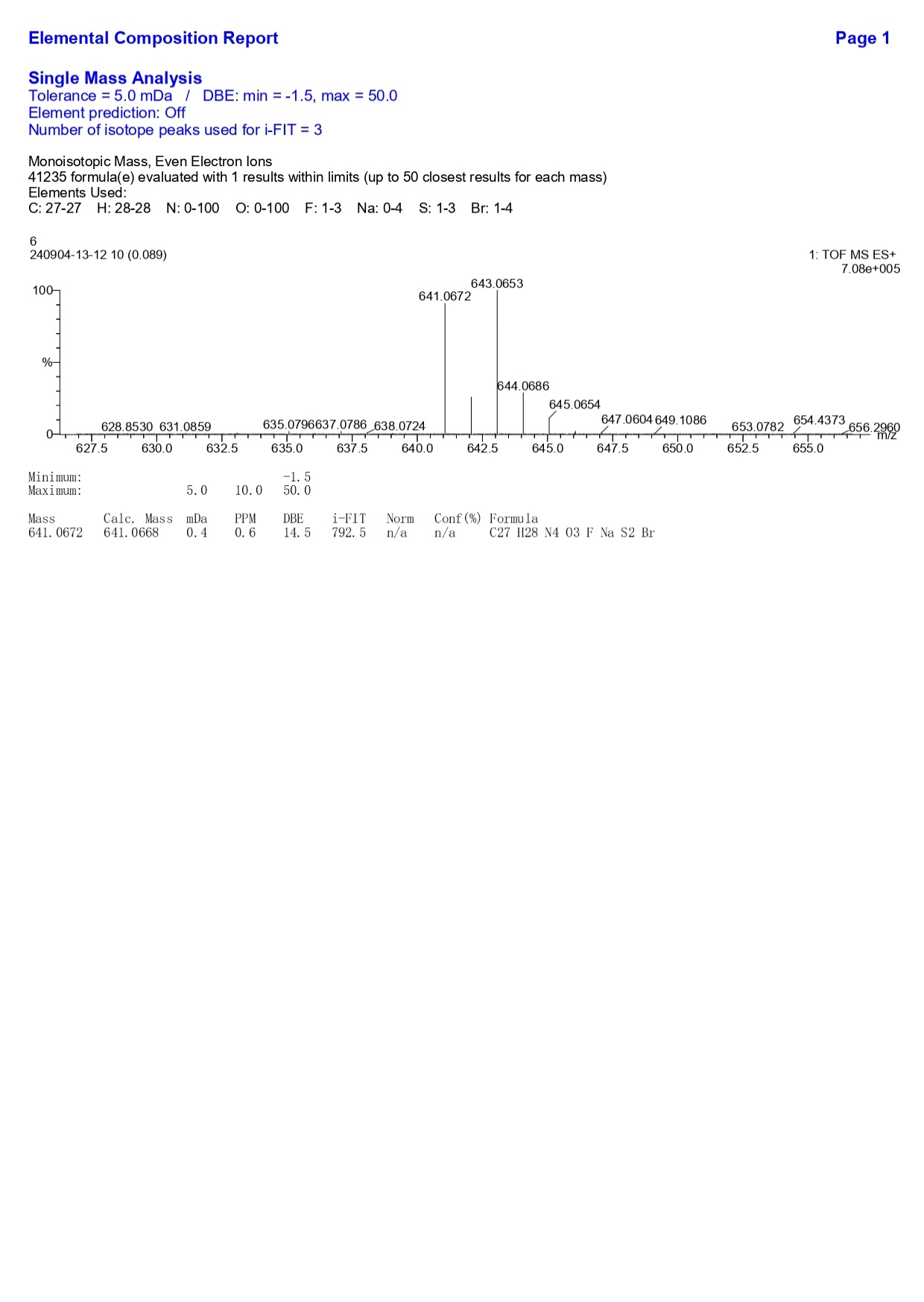

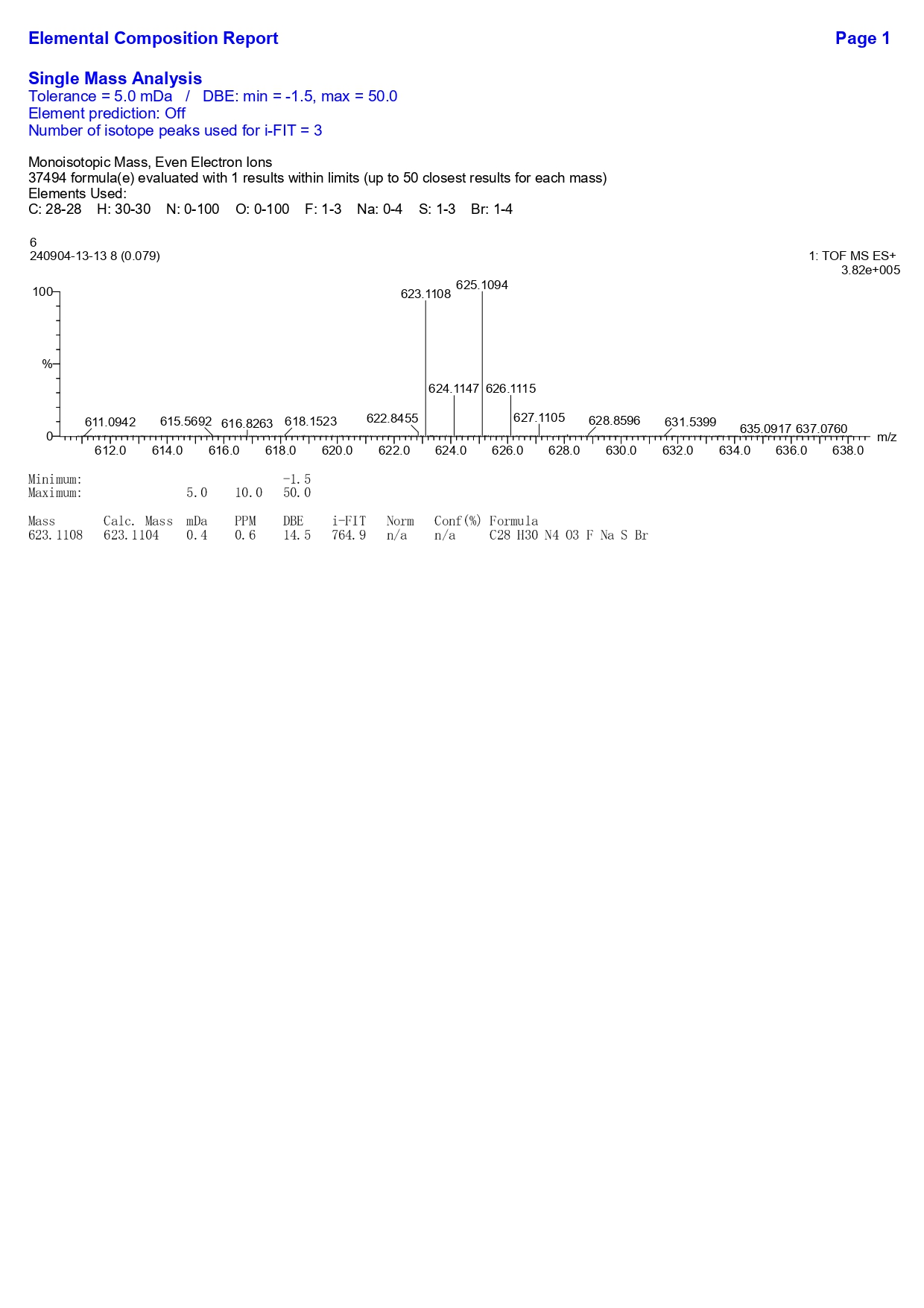

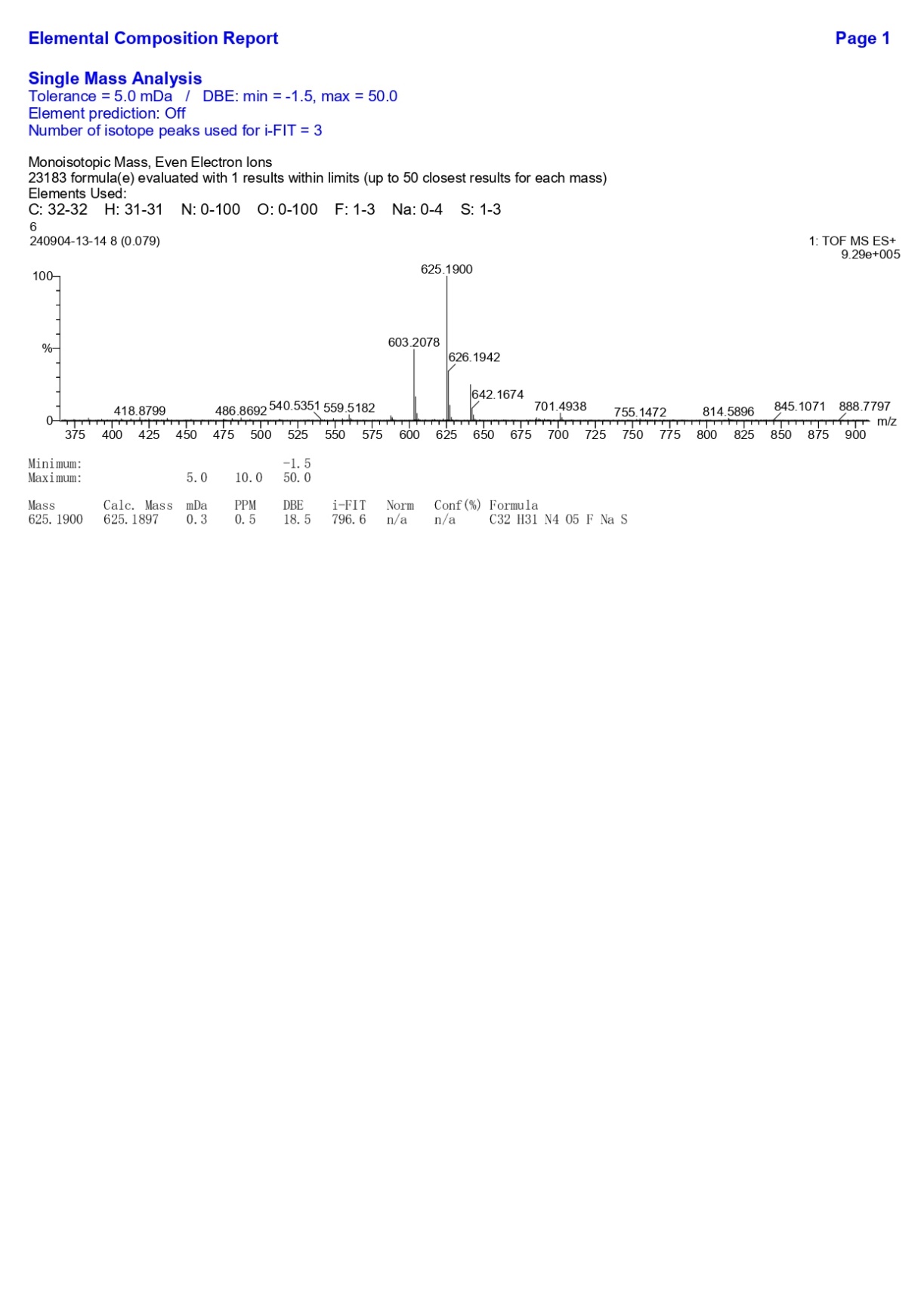

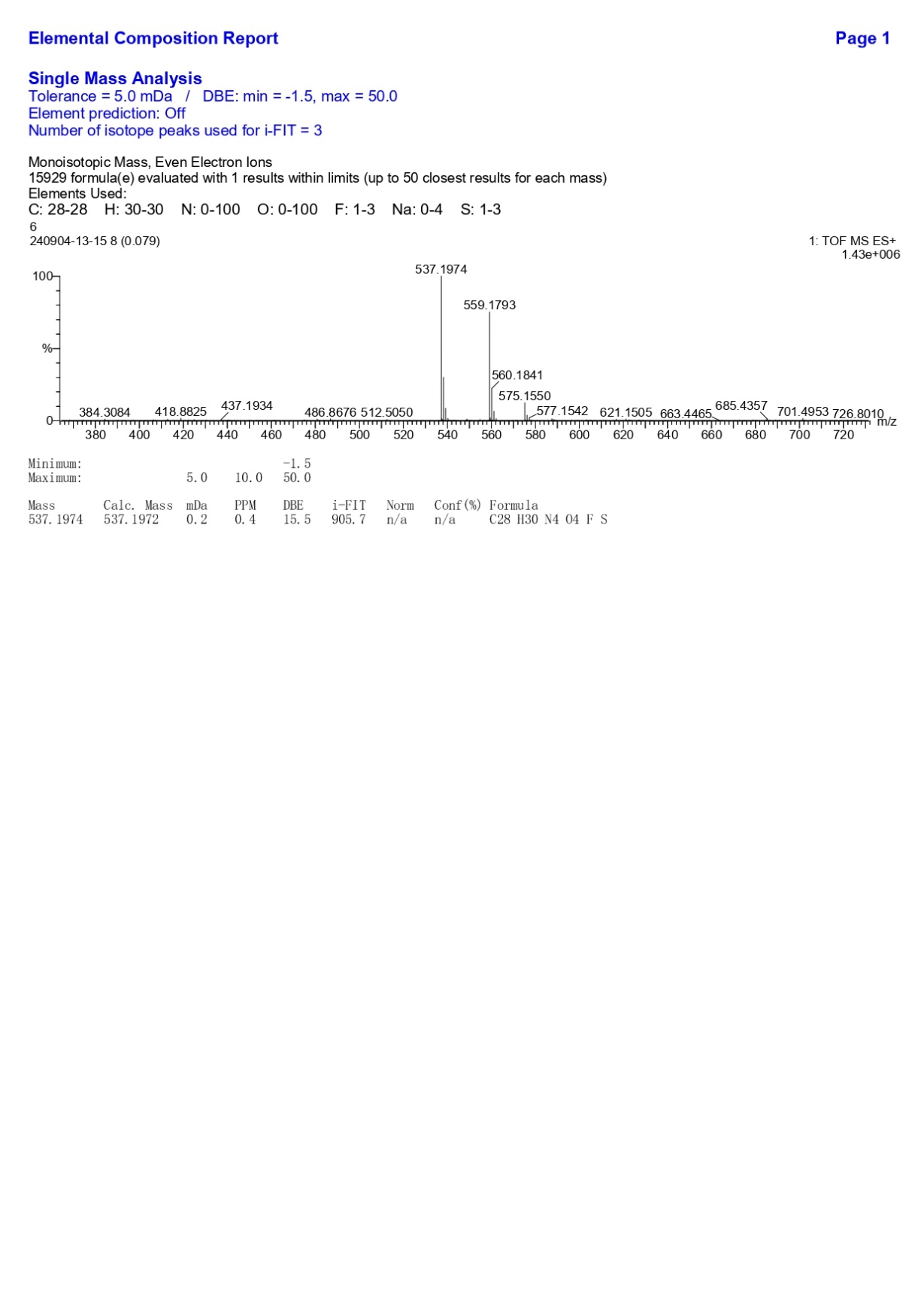

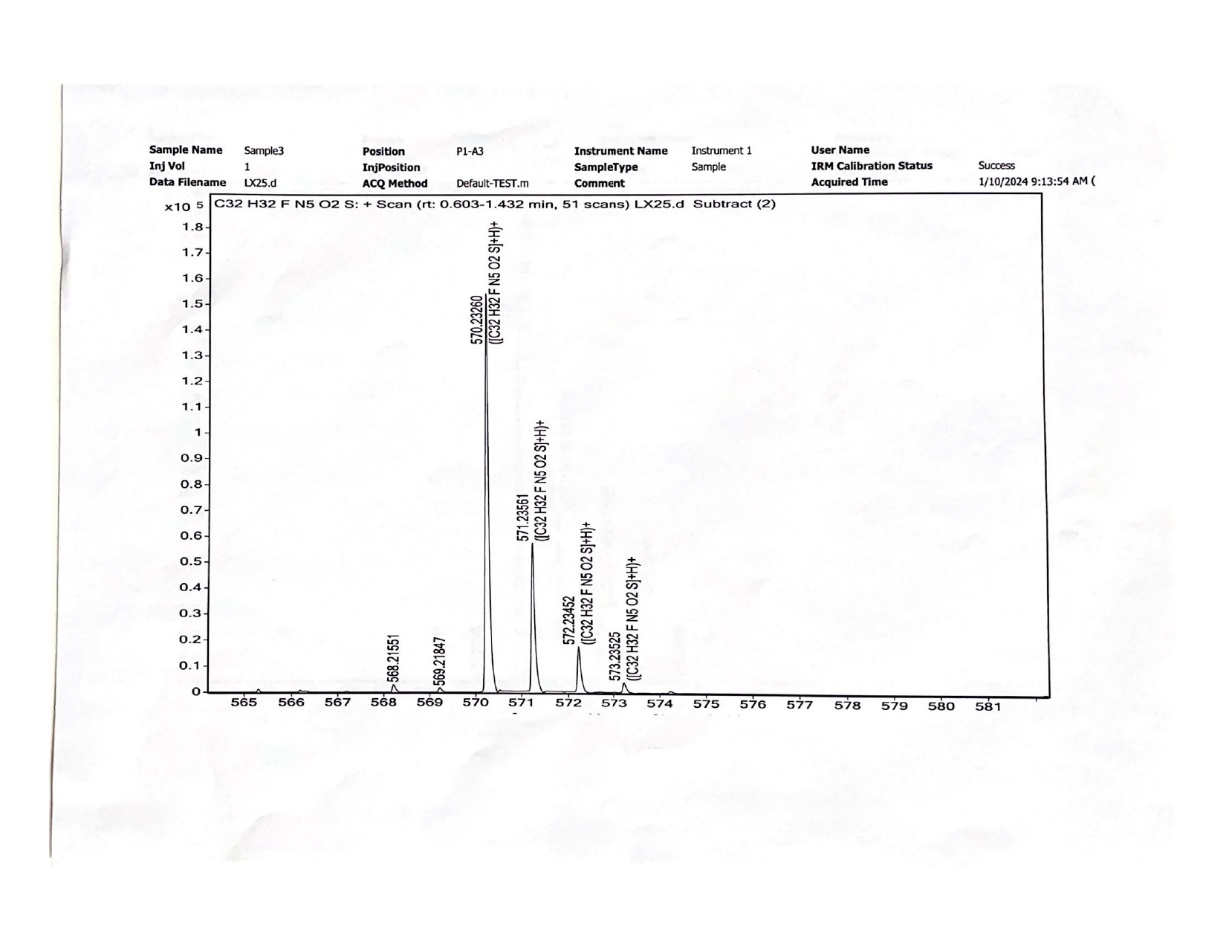

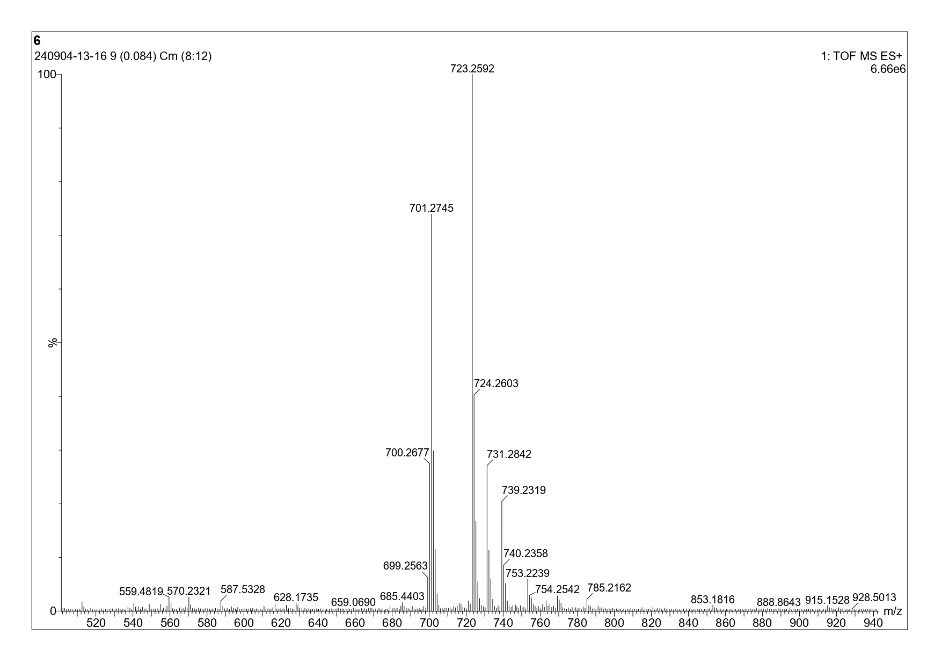

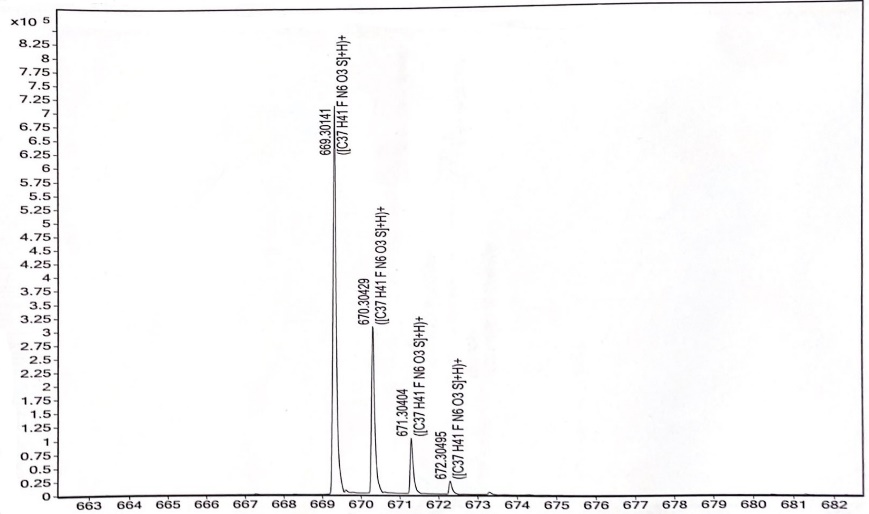

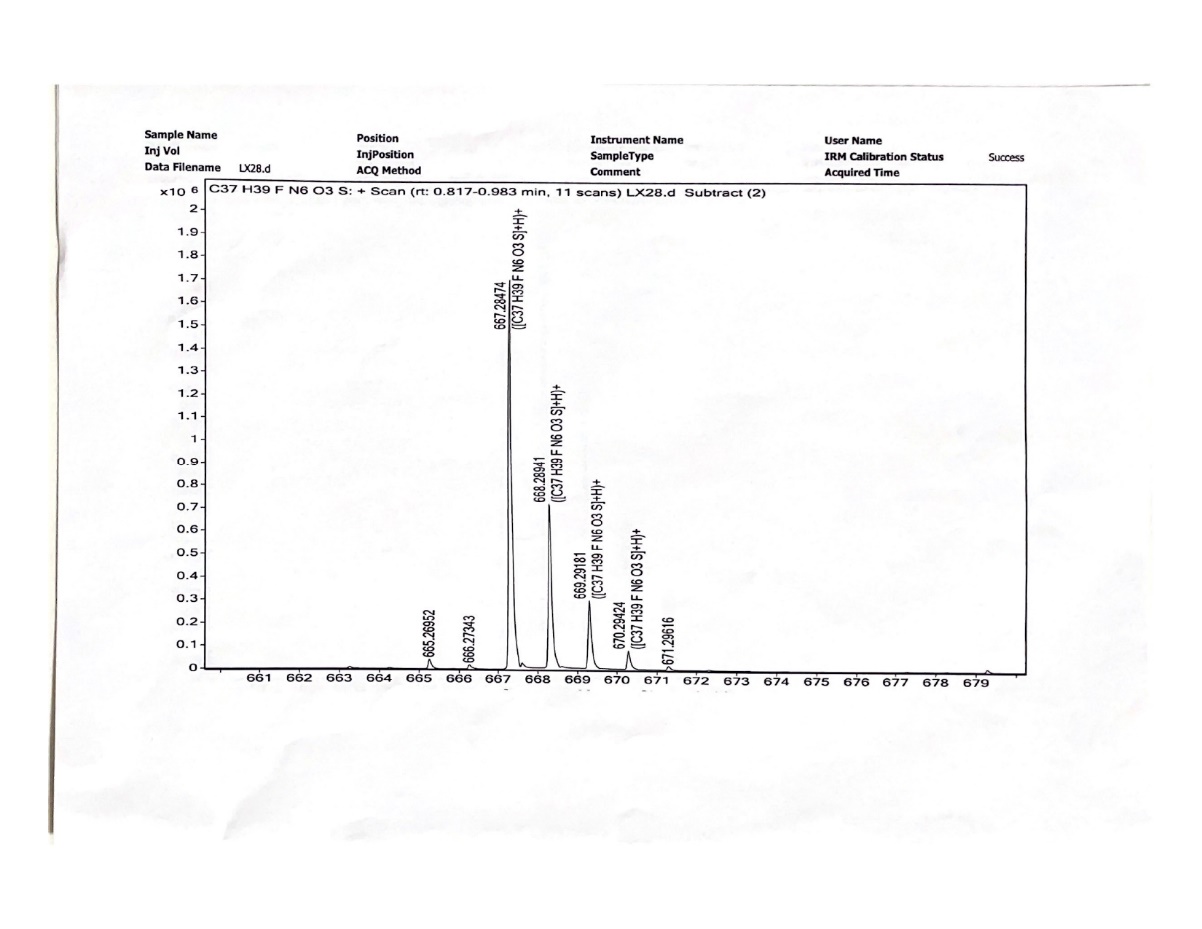


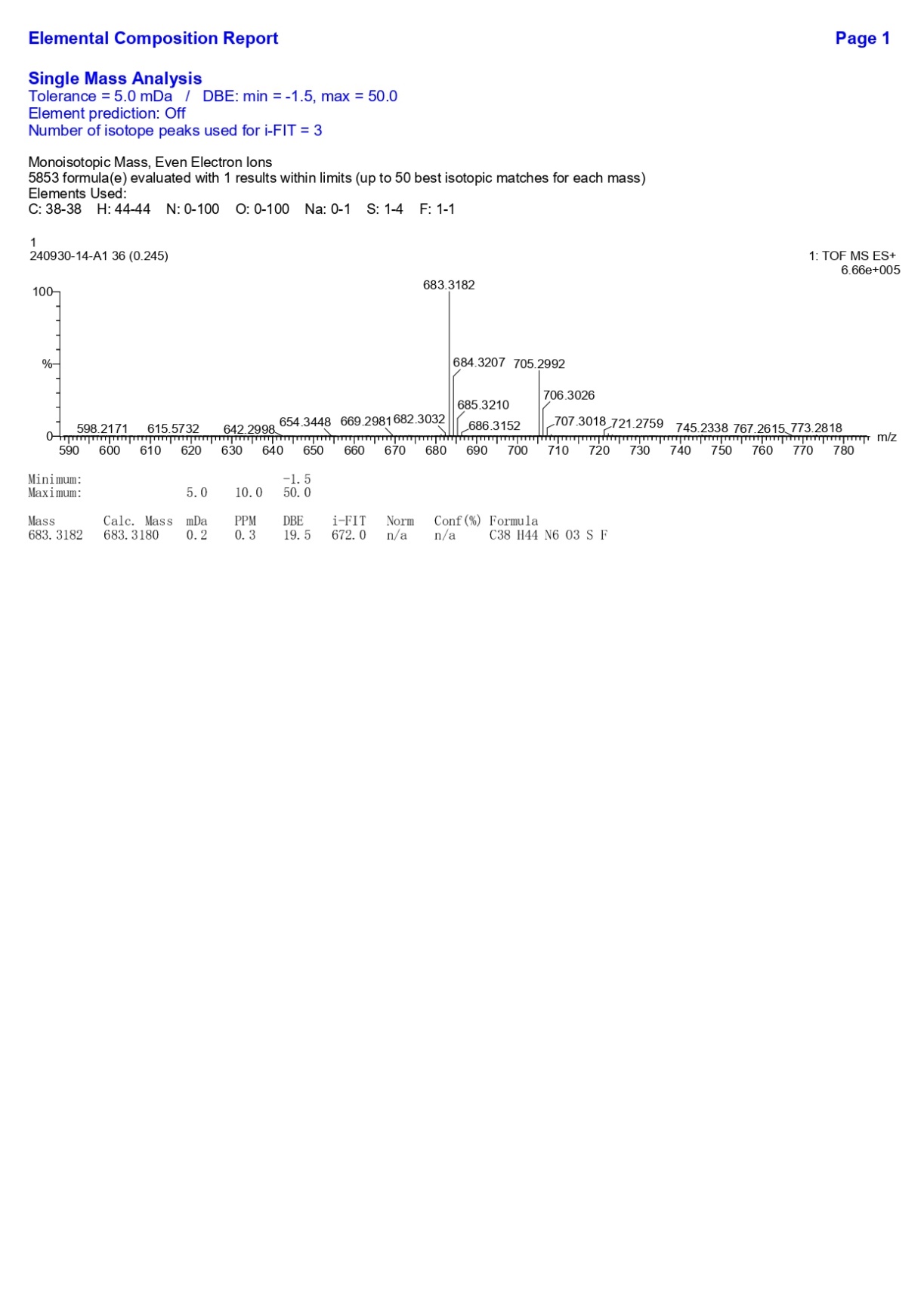

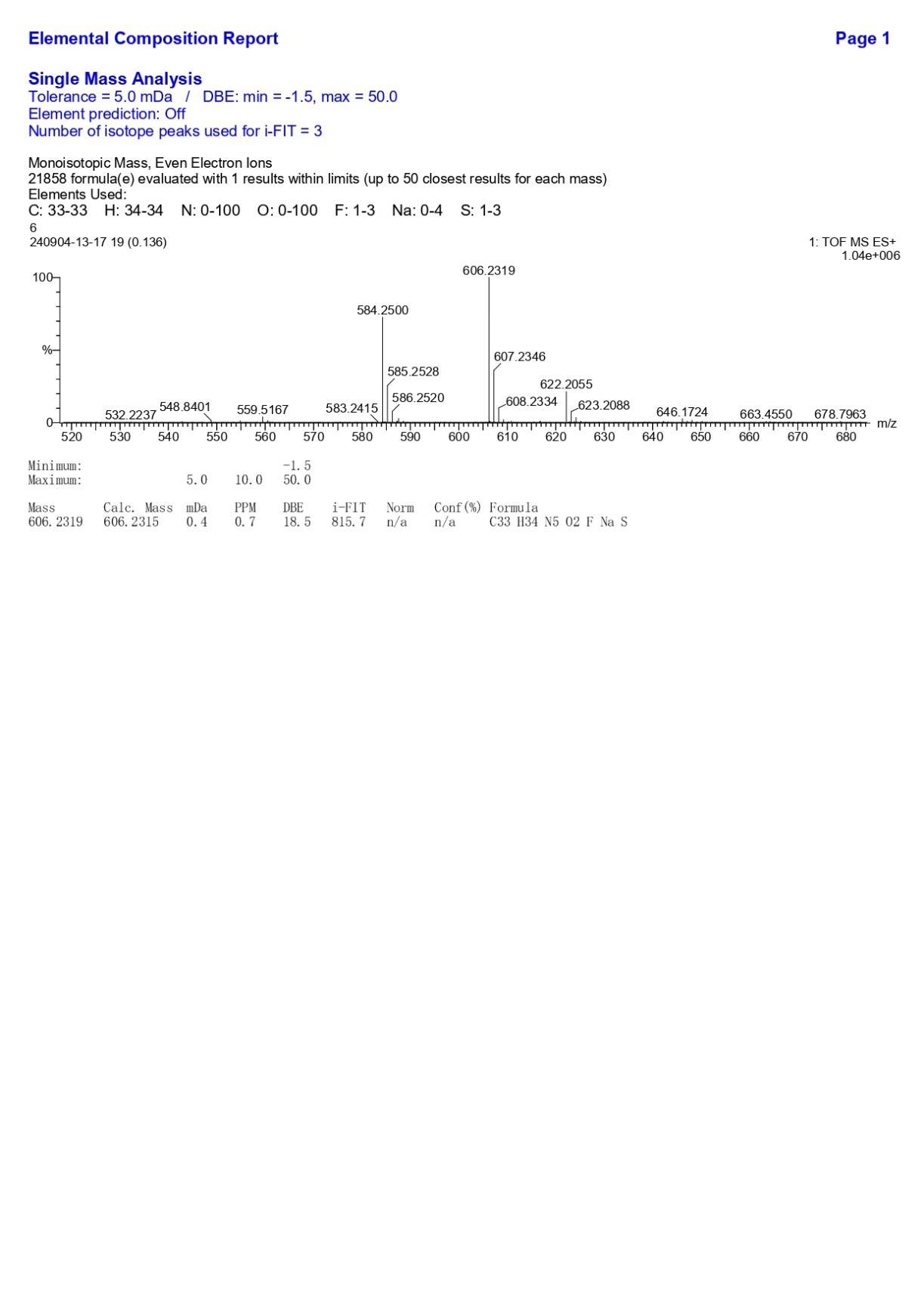

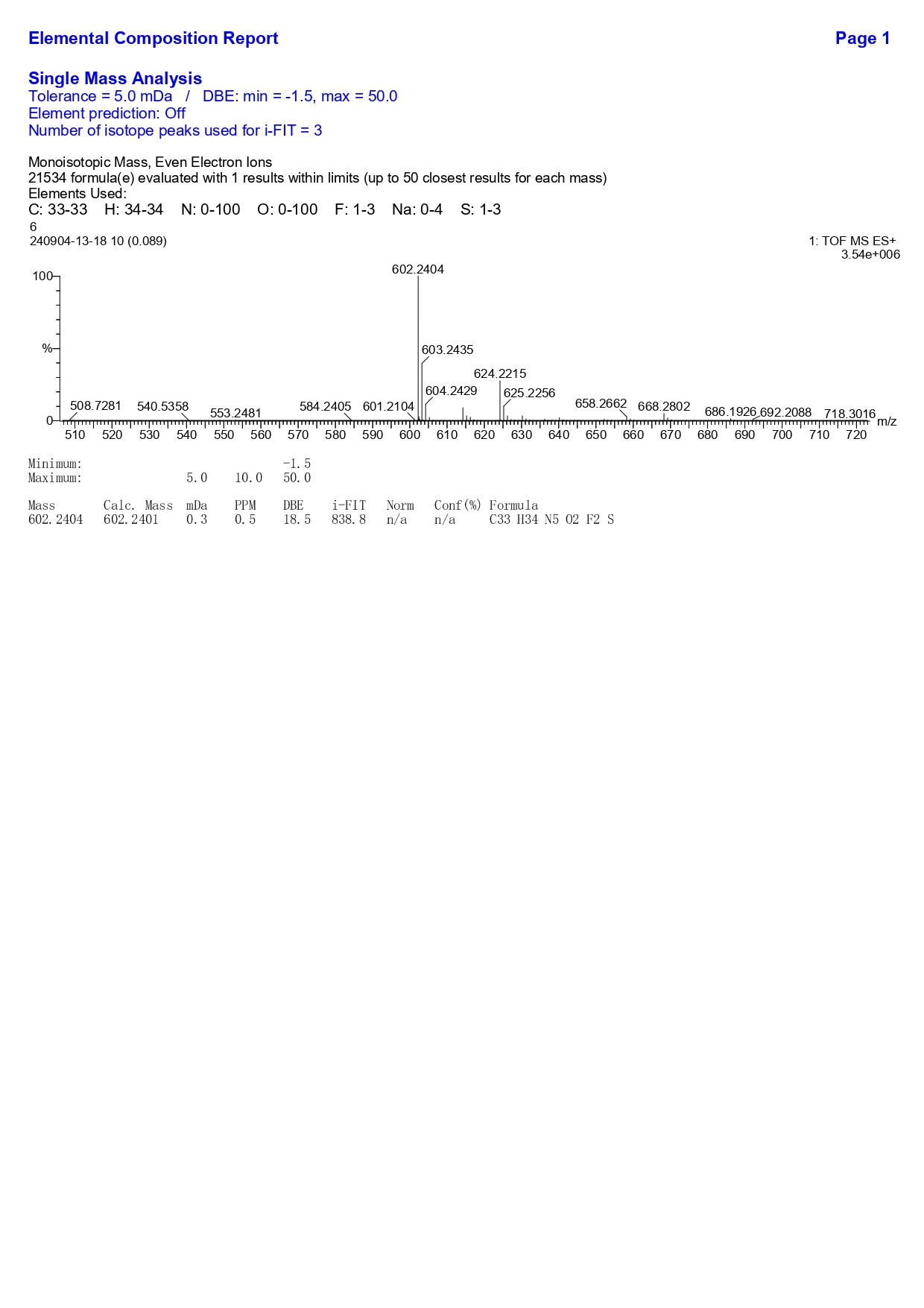

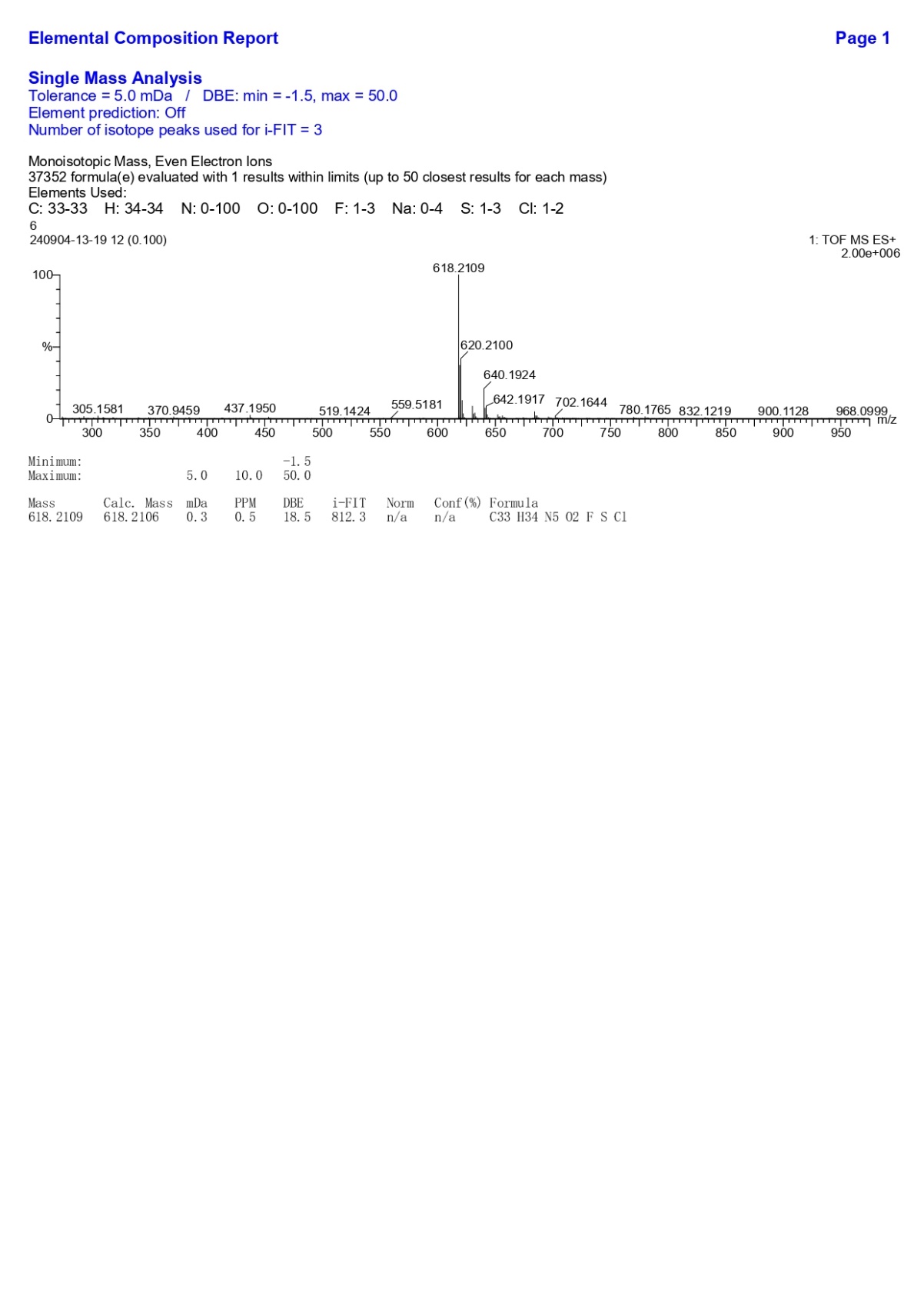

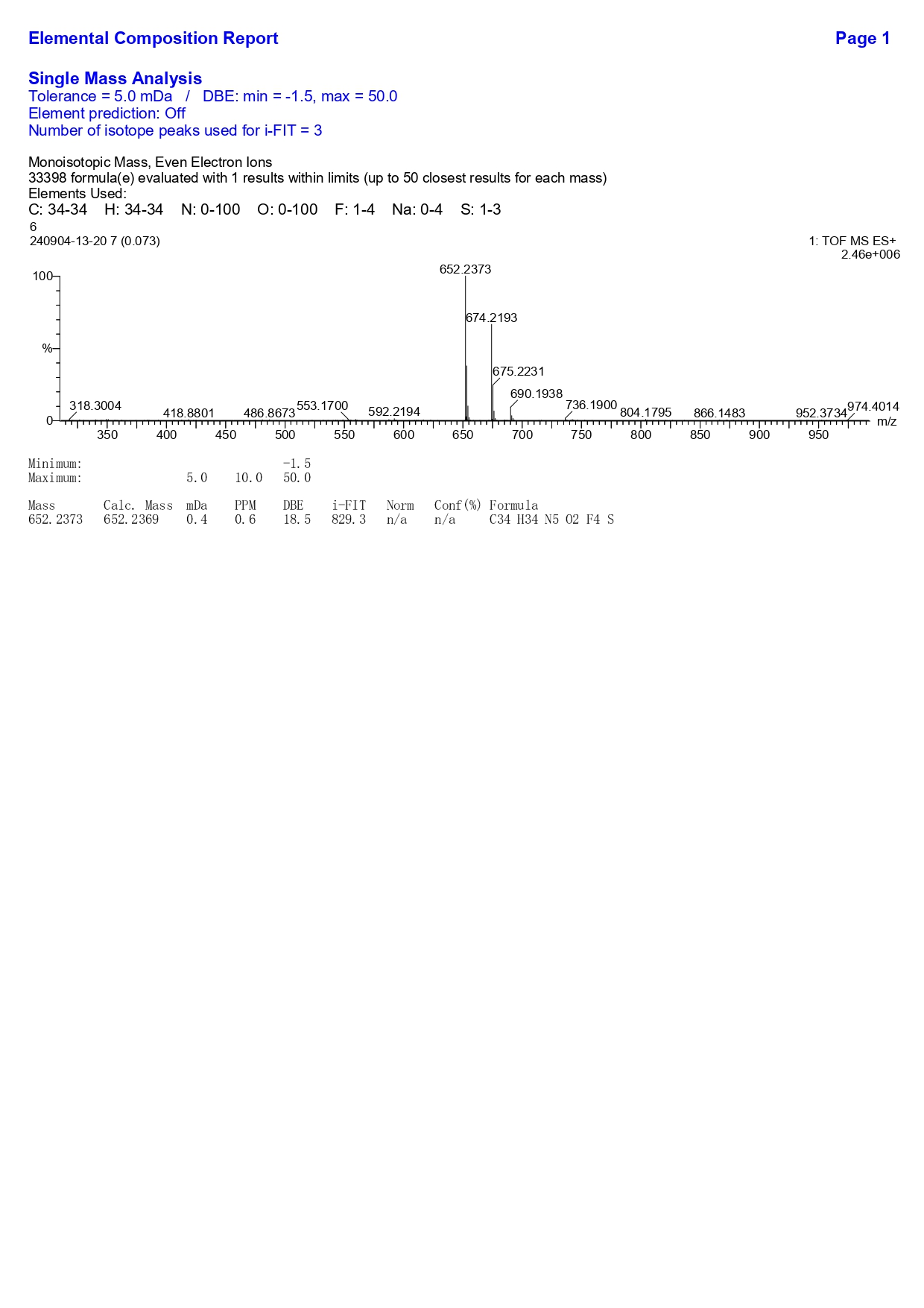


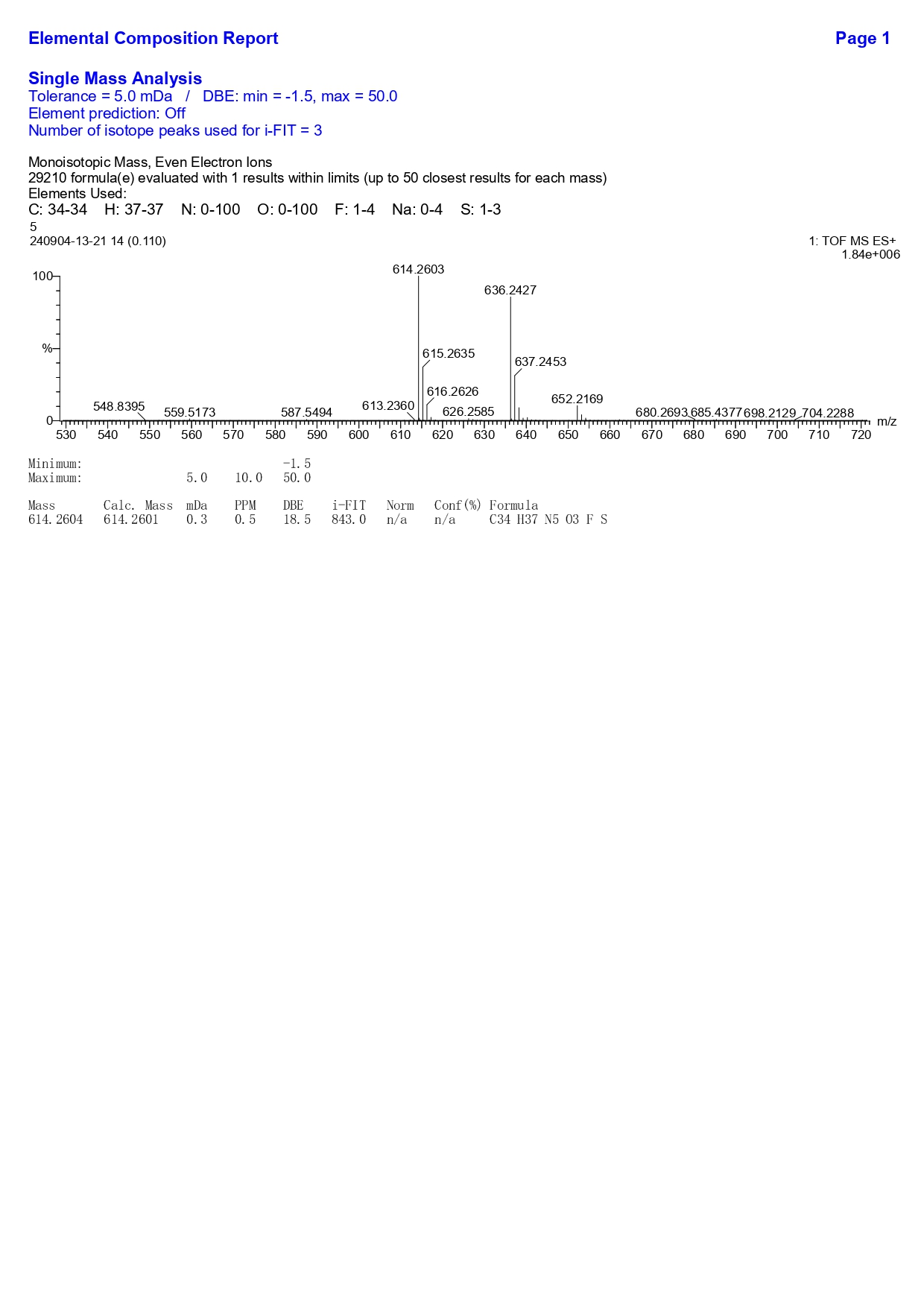


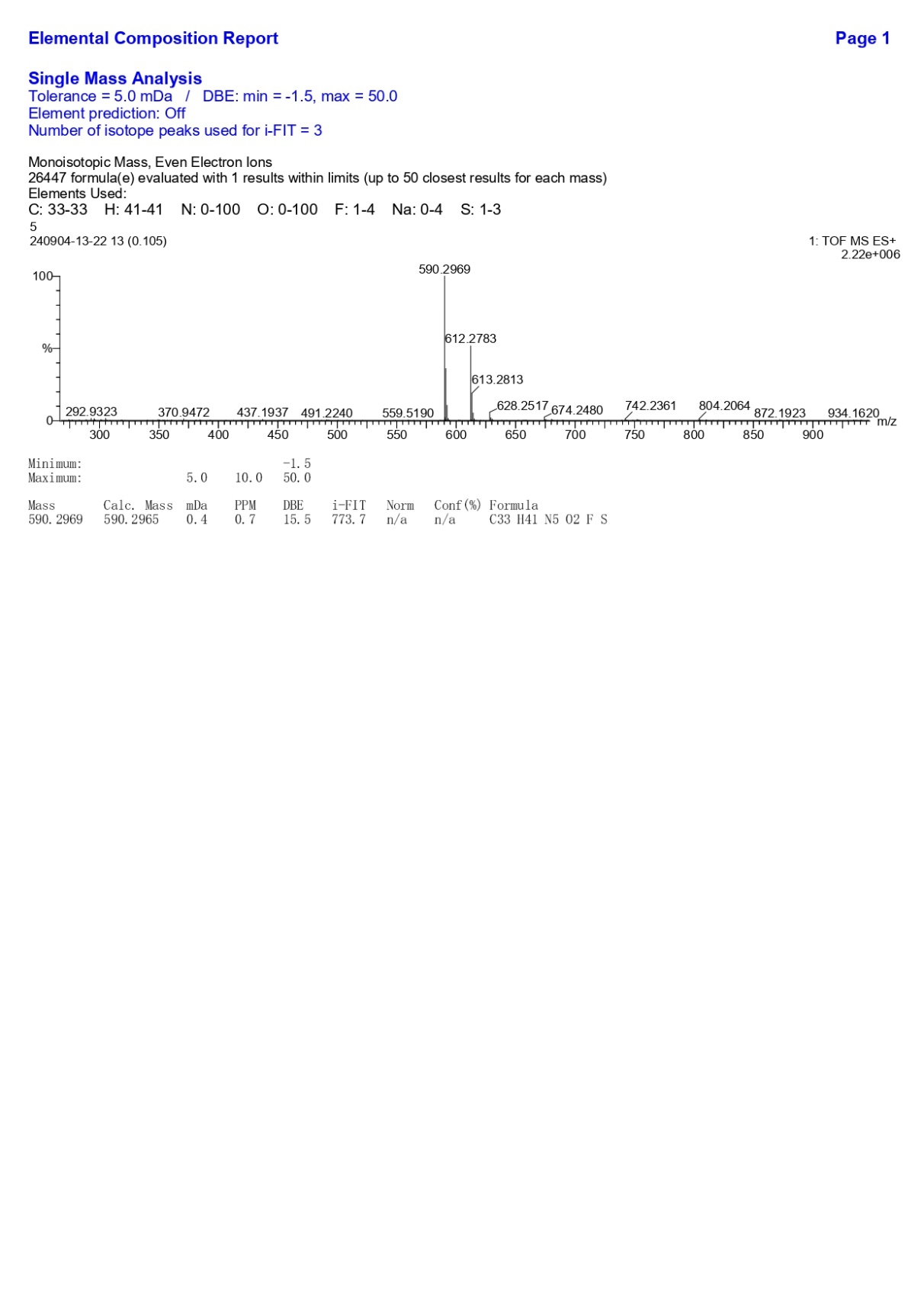


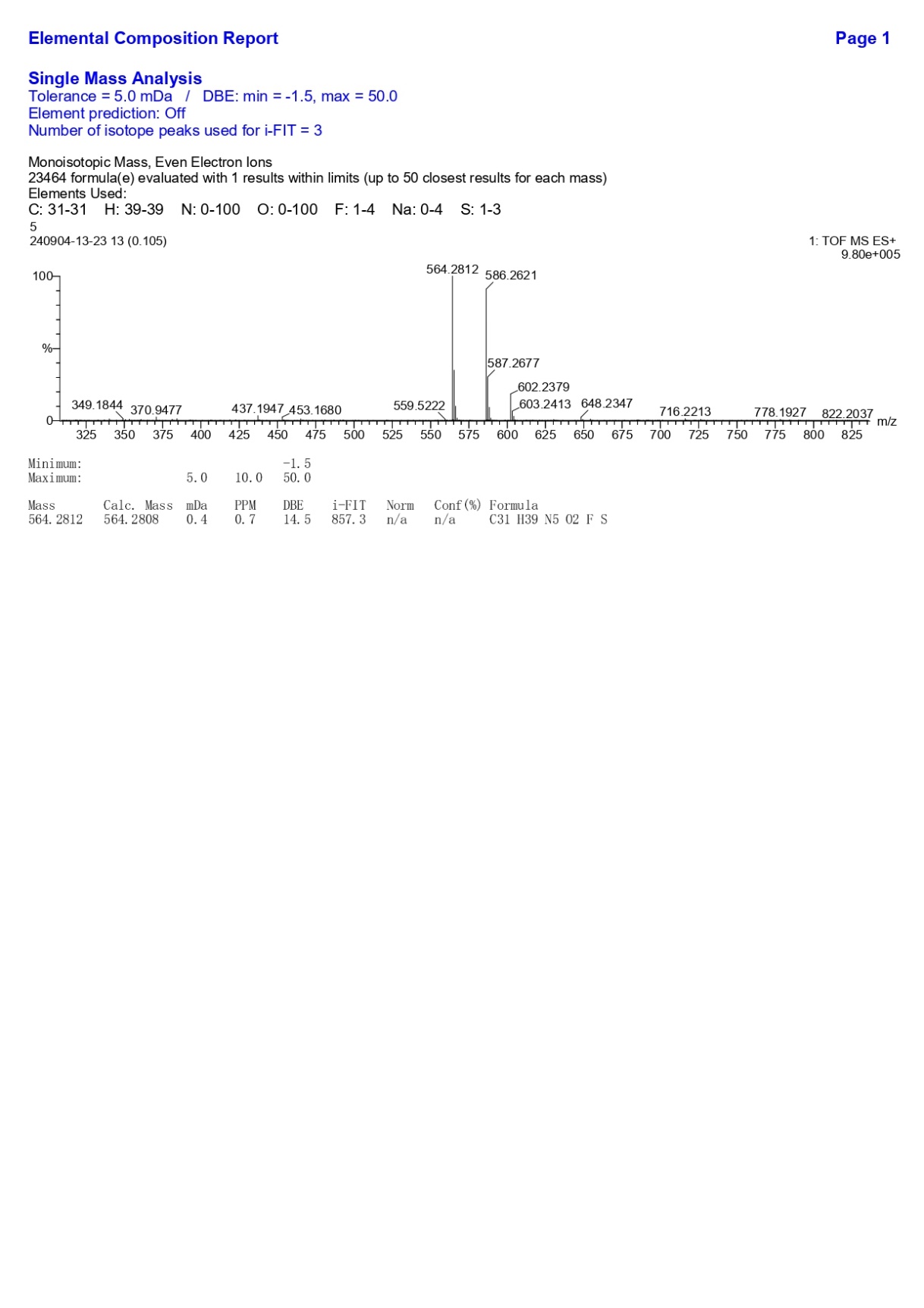


# Part D. HPLC Chromatograms of Tc3 Tested in Biological Assays.


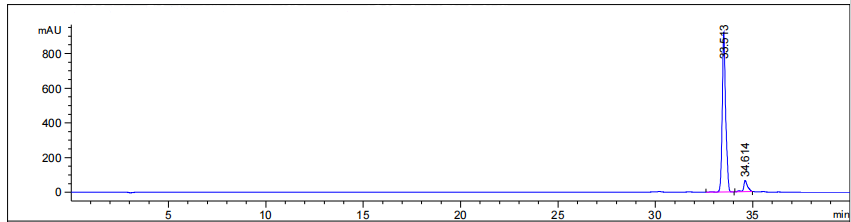


The HPLC method was developed on an UltiMate 3000 Dual-Gradient HPLC system (Sunnyvale, CA, USA), usting the column of ORBAX Eclipse XDB-C18 (5 µm, 4.6 × 150 mm, Agela Technologies). The elution consists of water (95%-5%) and methanol (5%-95%) at a flow rate of 1.0 mL/min. The column effluent was monitored at 210 nm and the chromatograms exhibited well resolved peak at reteantion times of 33.513 min. High purity **Tc3** was obtained by preparative liquid chromatography.
