# Supplementary material for: Discovery of indole analogue Tc3 as a potent pyroptosis inducer and identification of its combination strategy against hepatic carcinoma
Source: Theranostics. 2025 Jan 1;15(4):1285–303. doi: 10.7150/thno.102228 (PMC11729550; doi:10.7150/thno.102228)

original image

Figure 4

Uncropped gels for Western Blot in Figure 4

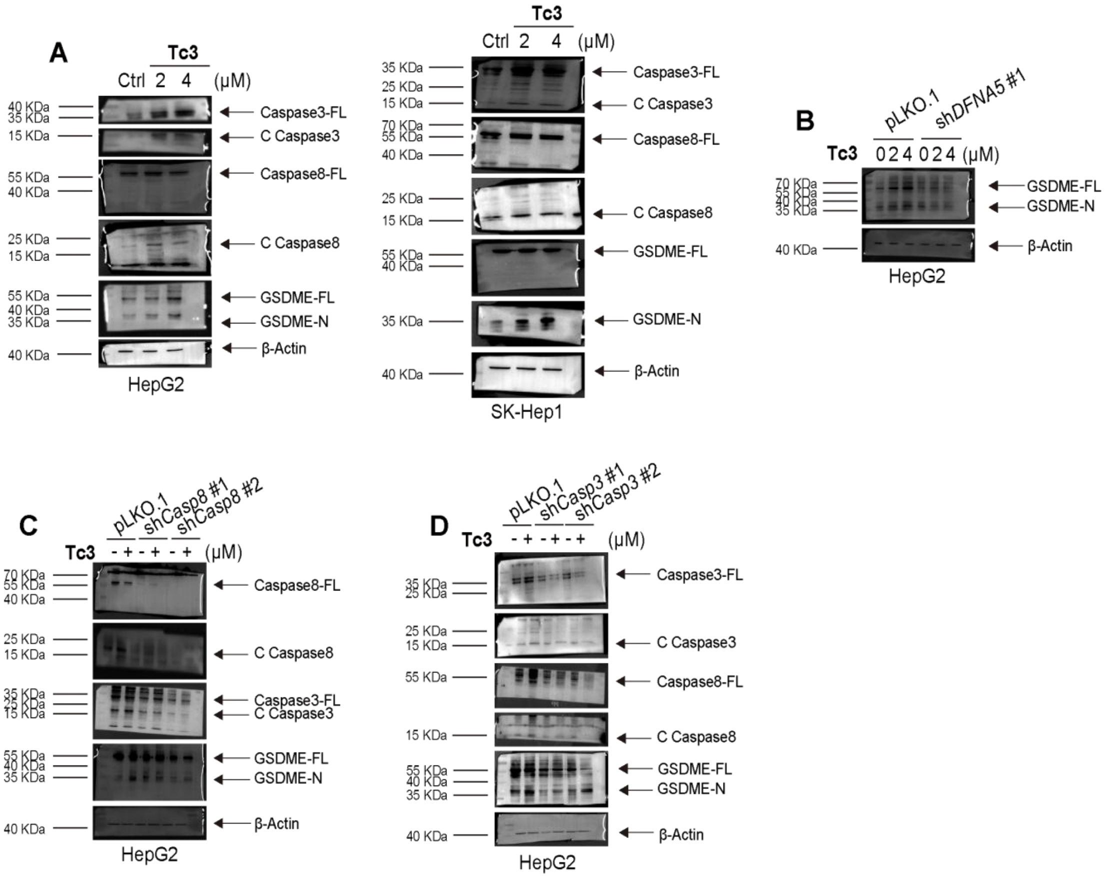

Figure 5

Uncropped gels for Western Blot in Figure 5

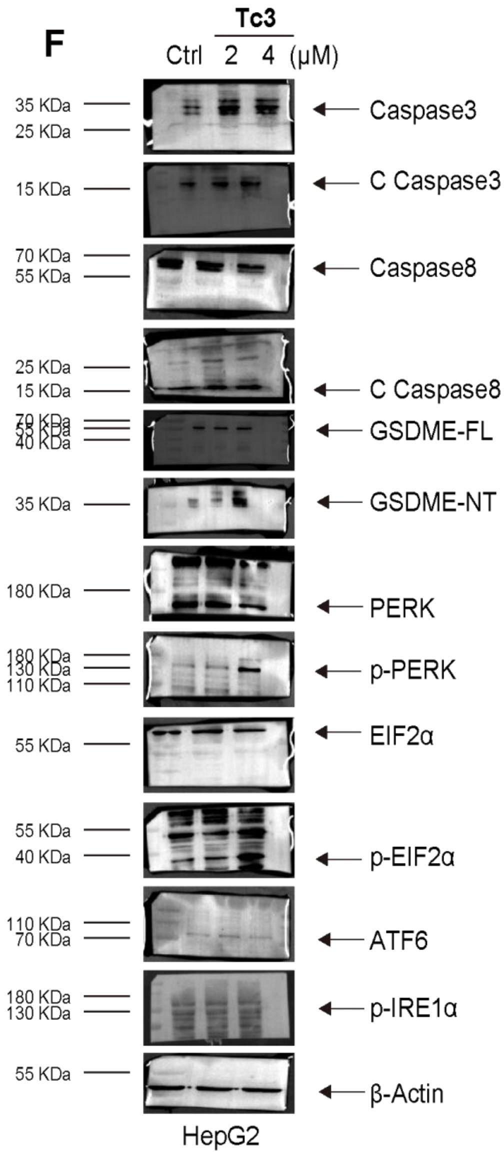

Figure 6

Uncropped gels for Western Blot in Figure 6

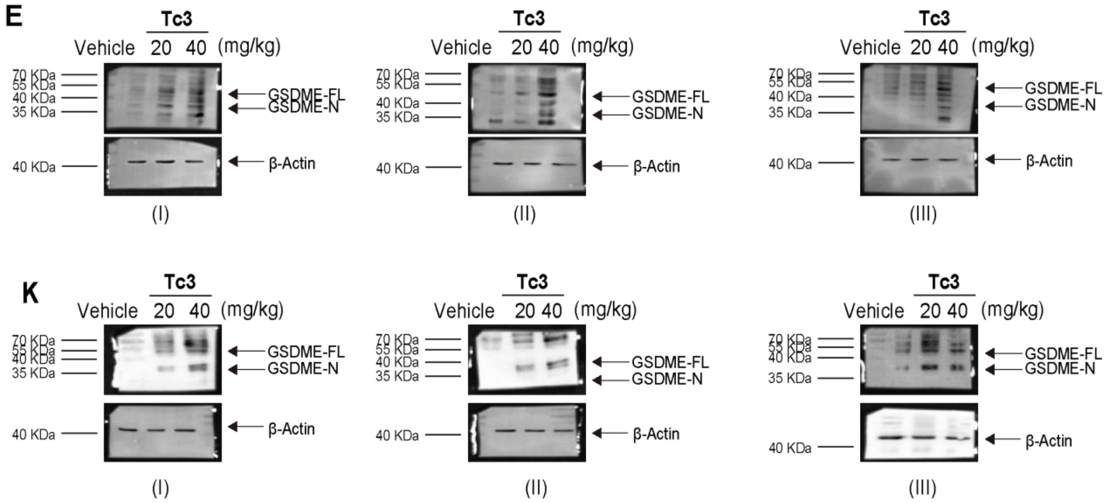

Figure 8

Uncropped gels for Western Blot in Figure 8

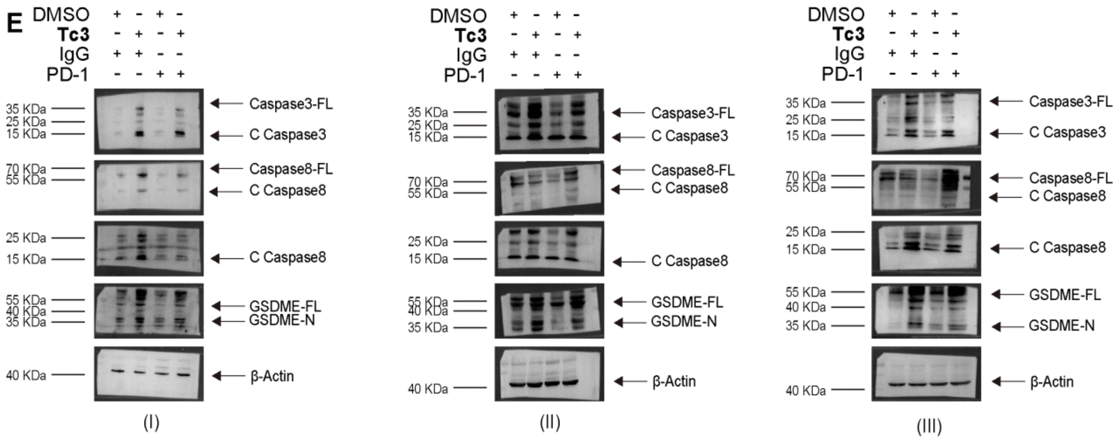

Figure S4

Uncropped gels for Western Blot in Figure S4

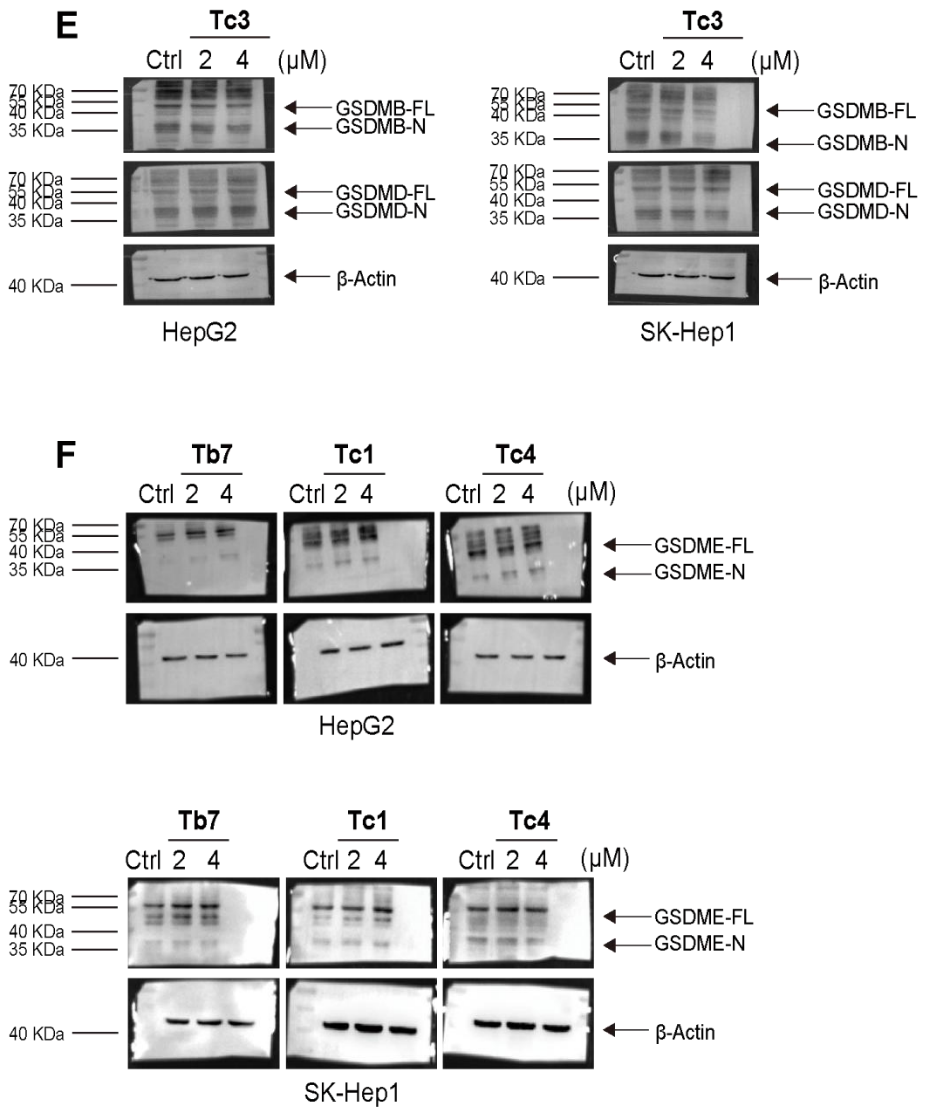

Figure S5

Uncropped gels for Western Blot in Figure S5

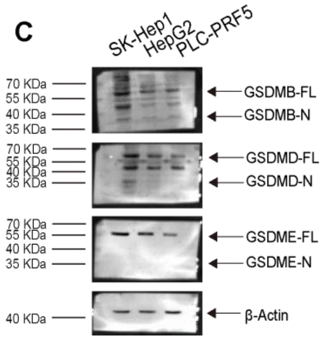

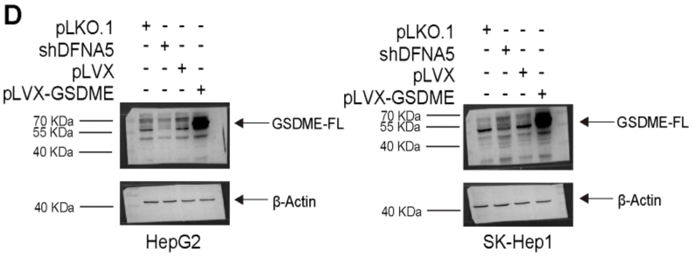

Figure S6

Uncropped gels for Western Blot in Figure S6

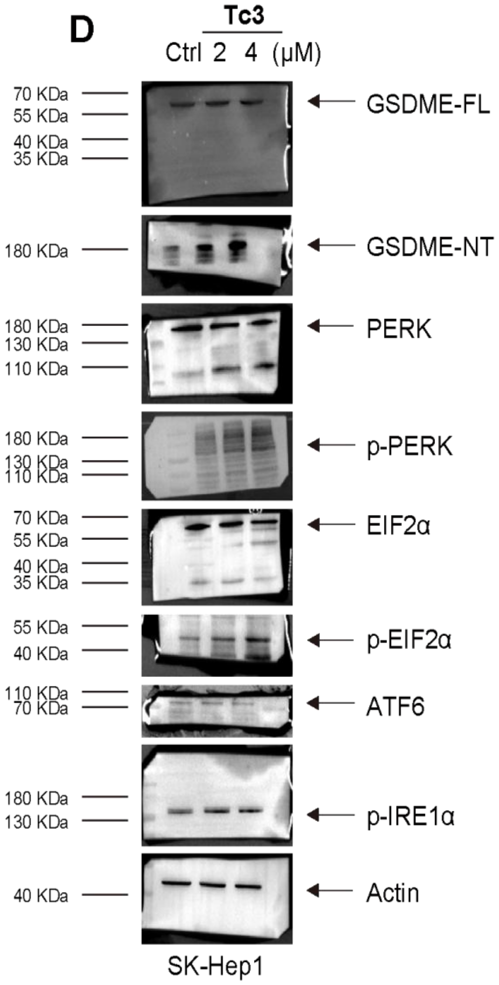

Figure S7

Uncropped gels for Western Blot in Figure S7

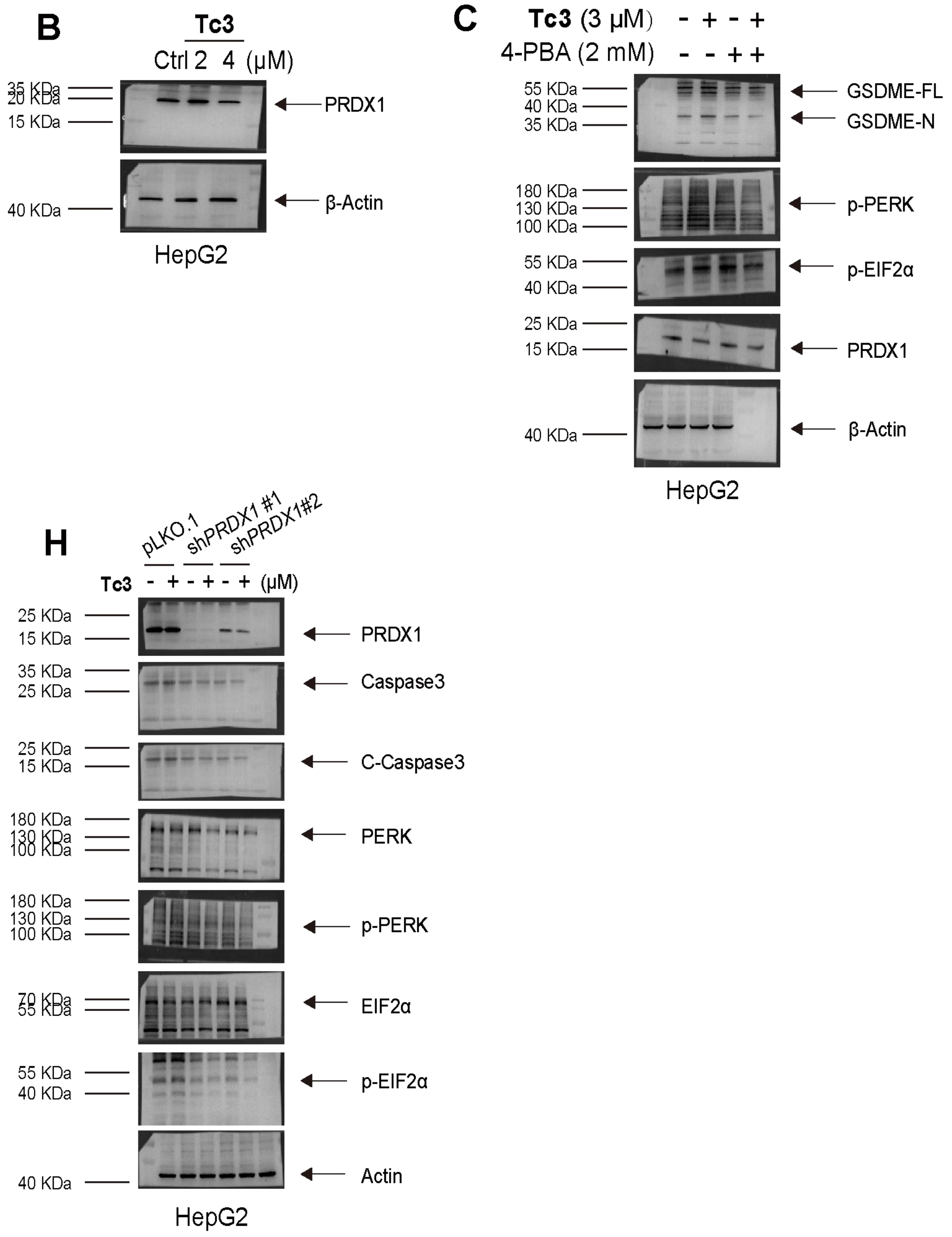

Supplement: Supplementary file 1 — Supplementary methods, figures and tables. [file thnov15p1285s1.zip › original wb image.pdf]
