# Supplementary material for: Discovery of indole analogue Tc3 as a potent pyroptosis inducer and identification of its combination strategy against hepatic carcinoma
Source: Theranostics. 2025 Jan 1;15(4):1285–303. doi: 10.7150/thno.102228 (PMC11729550; doi:10.7150/thno.102228)
Supplement: Supplementary file 1 — Supplementary methods, figures and tables. [file thnov15p1285s1.zip › Supporting Information.docx]

Supporting Information

**Discovery of indole analogue Tc3 as a potent pyroptosis inducer and identification of its combination strategy** **against** **hepatic carcinoma**

Xiao Hu^1†^, Xiaomei Tang^1†^, Xiaoman Tian^1^, Xing Lv^2^, Yuanyuan Zhang^1^, Yingyue Pang^1^, Weilong Deng^1^, Yali Wang^1^, Changliang Shan^1*^, Luqing Shang^1*^

^†^ These authors contributed equally to this work.

* Corresponding authors

E-mail addresses: changliangshan@nankai.edu.cn (Changliang Shan), shanglq@nankai.edu.cn (Luqing Shang).

^1^*State Key Laboratory of Medicinal Chemical Biology, College of Pharmacy,* *Nankai University, Tianjin 300353, People’s Republic of China*

^2^*Asymchem Pharmaceuticals (Tianjin) Co., Ltd., Tianjin 300457, People's Republic of China*

**Materials and Methods**

**Data and code availability**

All data are provided in the manuscript and supplement. The sequence and sample data have been deposited in NCBI database under Sequence Read Archive (SRA) (Bioproject identification number: PRJNA1145582), which includes our RNA-sequencing (RNA-seq).

**Anti-tumor activity assay (IC_50_ value)**

For half maximal inhibitory concentrations (IC_50_) analysis, the hepatic carcinoma cells (HepG2, SK-Hep1 and PLC-PRF5) were seeded in 24-well plates respectively, then treated with drugs at concentration gradient for three days. The cells were counted with hemocytometer. Cell inhibition rate = (A-B)/A × 100%. A is the cell quantity of the control group and B is the cell quantity of the drug treated group. The cell inhibition rate was transformed using its logarithm. And the drug IC_50_ value were calculated by Graphpad Prsim 9 software with curve fitting of the drug concentration and log (cell inhibition rate).

**Cell migration assay**

For transwell assay, the hepatic carcinoma cells were seeded in wells and put wells into 24-well plates pre-loaded DMEM medium supplemented with 20% FBS. The wells were added only DMEM medium with **Tc3**. The cells migrated through the membrane of wells in two days. Next, the wells were fixed by paraformaldehyde, stained by crystal violet and photographed for statistical analysis. For cell scratch assay, the hepatic carcinoma cells were seeded in 6-well plates and treated with **Tc3**. The scratch lines were obtained before the cell fusion reached almost 100%, and the cells were photographed at time gradient points for statistical analysis.

**Western blot assay**

The whole-cell protein was extracted by RIPA lysis buffer contained with protease and phosphatase inhibitor cocktail (MCE, HY-K0010) from cells treated by indole analogues. Proteins were separated by sodium dodecyl sulfate-polyacrylamide gel electrophoresis (SDS-PAGE) gel, and then blotted onto polyvinylidene difluoride membrane (Millipore, IPVH00010). The membranes were incubated with the primary antibodies and the following horseradish peroxidase-conjugated (HRP) secondary antibodies. Next, membranes were washed and developed by ECL regent (Millipore, WBKLS0500).

**Reactive Oxygen Species detection**

The hepatic carcinoma cells were seeded in 6-well plates and treated with **Tc3** for 24 h. The cells were then stained by DCFH-DA (10 μM) for 20 min, washed with PBS buffer for two times, and the fluorescence intensity was captured by a confocal microscope (Leica, TCS SP8).

**The stability study of Tc3**

The stability of **Tc3** was evaluated by an UltiMate 3000 Dual-Gradient HPLC system (Sunnyvale, CA, USA). The samples of solution were chromatographed on ZORBAX Eclipse XDB-C18 column(5 µm, 4.6 × 150 mm, Agela Technologies), using an eluent composed of: water (95%) and acetonitrile (5%) at a flow rate of 1.0 mL/min. The column effluent was monitored at 210 nm and the chromatograms exhibited well resolved peaks at retention times of 1.6058 min. The hydrolysis of **Tc3** was carried out in a phosphate buffer of PH 7.4 (50 ug/ml, initial concentration of 5 mM in methanol) over 150 minutes.

**Generation of cell lines with specific gene knockdown**

Briefly, the pLKO.1 or shRNA plasmids with psPAX2 and PMD2.G were transfected into HEK-293T cells via DNA transfection reagent (SignaGen®Laboratories, SL100688). And the cell supernatant contained Lenti-virus was collected after 24 hours. The Lenti-virus of shRNAs were used to infect the hepatic carcinoma cells and the stable-transfection cell lines were screened by puromycin (1 μg/mL) (Solarbio, P8230).

**Quantitative Real-time Polymerase Chain Reaction (qRT-PCR)**

The total RNA was extracted (Solarbio, R1100) and transcripted reversely to total cDNA (Yeasen, 11141ES60). Quantitative PCR was performed by using Hieff qPCR SYBR Green Master Mix (Yeasen, 11202ES08). The specific mRNA level was normalized to those of β*-Actin*. The relative quantitative analysis was analyzed by 2-*ΔΔ*Ct method.

**Toxicity test of Tc3 *in vivo***

For subsequent subchronic toxicity assay, Balb/c mice (female, 6-week-old) were divided into four groups randomly (n = 5) and treated with vehicle and **Tc3** (20 mg/kg, 40 mg/kg and 60 mg/kg) by i.p. The state, mortality and body weight of mice were monitored during 14 days. And the organ tissues of mice were collected for further H&E staining.

**H&E and immunohistochemistry (IHC) staining**

The formalin‐fixed and paraffin‐embedded tissues were cut into 5 μm sections. The sections were firstly soaked in xylene for removing paraffin and soaked in alcohol at decreasing concentration for tapping water. In the H&E staining assay, the sections were stained in Harris’s hematoxylin reagent and eosin reagent in turn. In the IHC staining, the sections were conducted antigen retrieval process and blocked with normal goat serum. Next, sections were incubated with the primary antibodies overnight and incubated with HRP-conjugated secondary antibodies. The tissue images were captured by a microscope.

**Immunofluorescence staining (IF)**

The mice tumor tissues were embedded into the OCT reagent and sliced into 10 μm sections via the freezing microtome. The sections were fixed by paraformaldehyde, blocked by 3% bovine serum albumin (BSA, Beyotime, ST2254), and incubated with the primary antibodies overnight. In the second day, the sections were incubated with the fluorescent‐conjugated antibodies and 4′,6‐diamidino‐2‐phenylindole (DAPI, Solarbio, C0065) for nuclei. The tissue fluorescent images were captured by a confocal microscope (Leica, TCS SP8) and analyzed by imageJ software.

**KEY RESOURCES TABLE**

| REAGENT or RESOURCE | SOURCE | IDENTIFIER |
| --- | --- | --- |
| Reagents | | |
| z-VAD-FMK | MCE | HY-16658B |
| 3-Methyladenine | MCE | HY-19312 |
| Deferoxamine mesylate | MCE | HY-B0988 |
| Cisplatin | MCE | HY-17394 |
| Oxaliplatin | MCE | HY-17371 |
| Raltitrexed | MCE | HY-10821 |
| Etoposide | MCE | HY-13629 |
| Camptothecin | MCE | HY-16560 |
| Gemcitabine | MCE | HY-17026 |
| 4-PBA | MCE | HY-A0281 |
| Regorafenib | MCE | HY-10331 |
| Cabozantinib | proteintech | CM03472 |
| Lenvatinib | proteintech | CM00941 |
| Axitinib | proteintech | CM00084 |
| Sorafenib  Necrostatin-1 | Proteintech  MCE | CM00088  HY-15760 |
| Antibodies |  |  |
| GSDME-N-terminal | abcam | ab215191 |
| DFNA5 | Santa Cruz | sc-393162 |
| GSDMB | abcam | ab215729 |
| GSDMD | abcam | ab209845 |
| cleaved-caspase3 (Asp175) | Cell Signaling Technology | #9661S |
| caspase8 | proteintech | 13423-1-AP |
| Ki67 | proteintech | 27309-1-AP |
| PERK | abcam | ab229912 |
| p-PERK (Thr982) | Affinity | #DF7576 |
| EIF2α | abcam | ab169528 |
| p-EIF2α | S51, ZENBIO | R22946 |
| ATF6 | Cell Signaling Technology | #65880 |
| p-IRE1α | S724, abcam | ab124945 |
| β-actin | YEASEN | 30101ES50 |
| *InVivo* MAb anti-mouse PD-1 (CD279) | BioXcell, RMP1-14 | Cat# BE0146 |
| *InVivo* MAb rat IgG2a isotype control | 2A3 | Cat# BE0089 |
| FITC anti-mouse/human CD11b | BioLegend | 101205 |
| APC anti-mouse F4/80 | BioLegend | 123115 |
| PE anti-mouse CD86 | BioLegend | 105007 |
| Alexa Flour® 700 anti-mouse CD206 (MMR) | BioLegend | 141773 |
| APC anti-mouse Ly-6G | BioLegend | 127613 |
| FITC anti-mouse CD45 | BioLegend | 103107 |
| APC anti-mouse CD8a | BioLegend | 100711 |
| Pacific Blue^TM^ anti-mouse IFN-γ | BioLegend | 505817 |
| Alexa Fluor® 647 anti-mouse CD8a | BioLegend | 100727 |
| Alexa Fluor® 647 anti-mouse Ly-6G | BioLegend | 127609 |
| purified anti-mouse CD16/32 | BioLegend | 101301 |
| Plasmids | | |
| pLKO.1 | TranSheepBio | N/A |
| shcaspase1 | TranSheepBio | N/A |
| shcaspase3 | TranSheepBio | N/A |
| shcaspase4 | TranSheepBio | N/A |
| shcaspase8 | TranSheepBio | N/A |
| shGSDMB | TranSheepBio | N/A |
| shGSDMC | TranSheepBio | N/A |
| shGSDMD | TranSheepBio | N/A |
| shDFNA5 | TranSheepBio | N/A |
| shDFNB59 | TranSheepBio | N/A |
| psPAX2 | TranSheepBio | N/A |
| PMD2.G | TranSheepBio | N/A |
| pLVX-vector | General Biology | N/A |
| pLVX-GSDME | General Biology | N/A |
| Oligonucleotides | | |
| caspase1 F | CATCCCACAATGGGCTCTGT | N/A |
| caspase1 R | GCATCTGCGCTCTACCATCT | N/A |
| caspase3 F | GATGCAGCAAACCTCAGGGA | N/A |
| caspase3 R | ACCATGGCTCAGAAGCACAC | N/A |
| caspase4 F | AGTTTGACCATCTGCCTCCG | N/A |
| caspase4 R | GGTCTGGTAGCAAATGCCCT | N/A |
| caspase8 F | CAGAGCCTGAGAGAGCGATG | N/A |
| caspase8 R | AGGCTGAGGCATCTGTTTCC | N/A |
| GSDMA F | GCTCACCTTCAGACCCAACA | N/A |
| GSDMA R | GCGCTATCTGGCATTTCTGC | N/A |
| GSDMB F | GAAGAGCAGCAGTTTGTGGC | N/A |
| GSDMB R | TGCCTCAGGGTCATAGTCCA | N/A |
| GSDMC F | GAGGGGACAACCTGTACGTG | N/A |
| GSDMC R | TCTGAAGAGTCAGCGCCTTC | N/A |
| GSDMD F | TAGTCCGGAGAGTGGTCCAG | N/A |
| GSDMD R | ACCATGAGCTTGAGGGCTTC | N/A |
| DFNA5 F | CTGGAACCAGTGTGCGATGA | N/A |
| DFNA5 R | GCGCTATCTGGCATTTCTGC | N/A |
| DFNB59 F | CATGCTGGAATTCGAGGGGA | N/A |
| DFNB59 R | GCAGTGCAAATGTTGCCGTT | N/A |
| shRNA | | |
| caspase1 | CCAGATATACTACAACTCAAT | N/A |
| caspase3 | GCGAATCAATGGACTCTGGAA, CCGAAAGGTGGCAACAGAATT | N/A |
| caspase4 | CCGAGGAATGGAGCTGACTTT | N/A |
| caspase8 | ACATGAACCTGCTGGATATTT, CACCAGGCAGGGCTCAAATTT | N/A |
| GSDMA | CTGAAGGAGATGCAAGATCAA | N/A |
| GSDMB | GCCTTGTTGATGCTGATAGAT | N/A |
| GSDMC | GCCACCAAATTACGTCAGTTT | N/A |
| GSDMD | CAGCACCTCAATGAATGTGTA | N/A |
| DFNA5 | GCATTCATAGACATGCCAGAT, GATGATGGAGTATCTGATCTT | N/A |
| DFNB59  PRDX1 | GGTTGAGAAATATCCTATTTG  GGCTTTCAGTGATAGGGCAGAA  GGATGAGACTTTGAGACTAGTT | N/A  N/A  N/A |

**Table S1. Chromatographic conditions for assay of Tc3**

| HPLC Parameter | Details |
| --- | --- |
| Column | ZORBAX Eclipse XDB-C18 (5 µm, 4.6 × 150 mm, Agela Technologies) |
| Flow rate | 1.0 mL/min |
| UV detection | 210 nm |
| Column temperature | 25 ^o^C |
| Injection volumn | 10.0 ul |
| Mobile phase | 95% water and 5% acetonitrile |
| Method | constant gradient elution |
| Run time | 150 min |

**Figure S1**


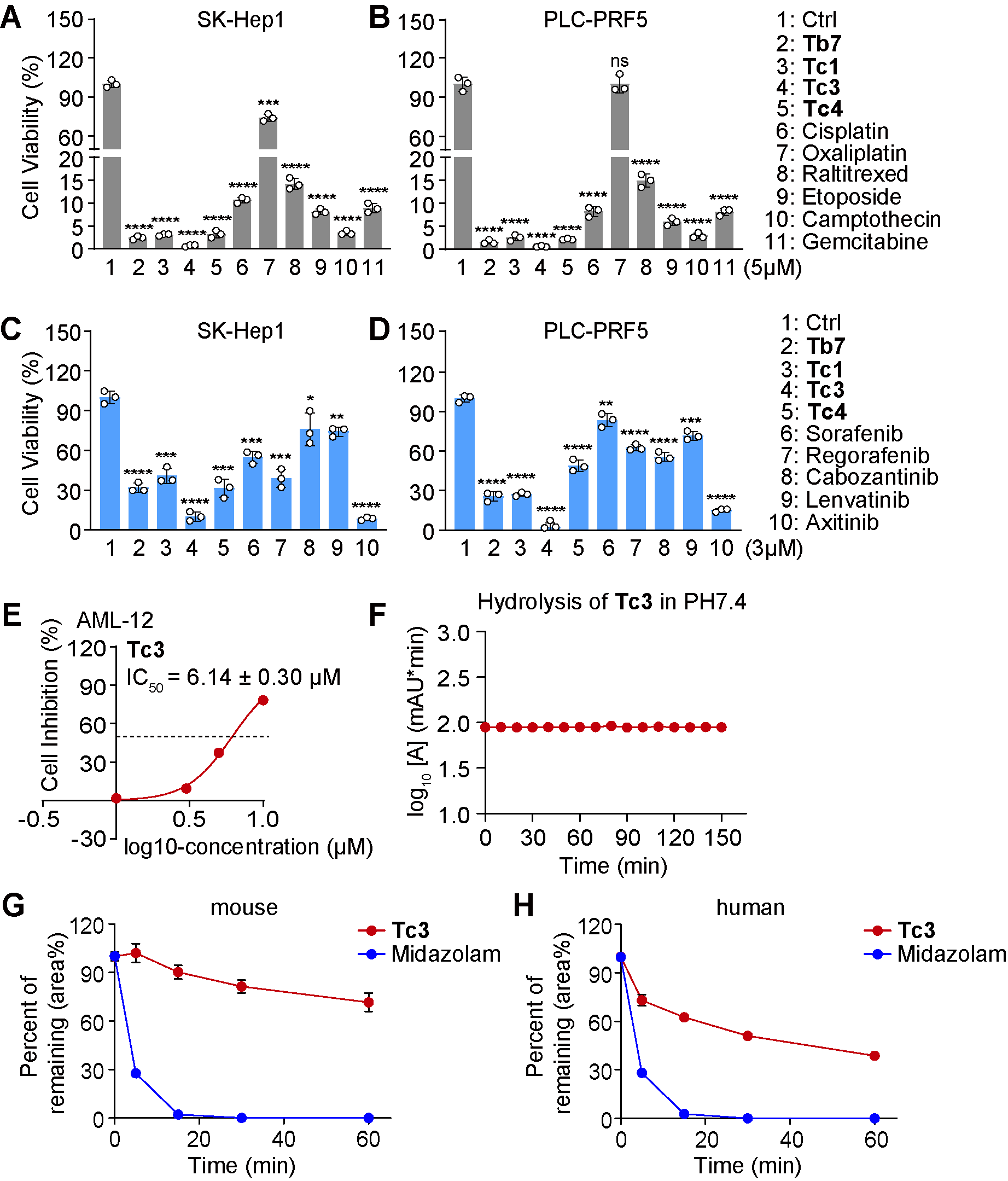


**Figure S1 Identification pharmacological properties of Tc3 *in vitro*.**

(A-B) The cell viability rate of SK-Hep1 (A) and PLC-PRF5 (B) cells treated by **Tb7**, **Tc1**, **Tc3**, **Tc4** and other clinical anti-tumor drugs.

(C-D) The cell viability rate of SK-Hep1 (C) and PLC-PRF5 (D) cells treated by **Tb7**, **Tc1**, **Tc3**, **Tc4** and other clinical anti-hepatoma drugs.

(E) Dose-response curves for **Tc3** inhibiting the growth of AML-12 cells.

(F) The hydrolytic stability of **Tc3** in phosphate buffer of PH 7.4.

(G-H) **Tc3**’s residual percentage (area%) in the mouse (G) and human (H) liver microsome incubation system.

(Data are mean ± SD of three biologically independent experiments. **p* < 0.05, ***p* < 0.01, ****p* < 0.001 and *****p* < 0.0001, ns, no significant).

**Figure S2**


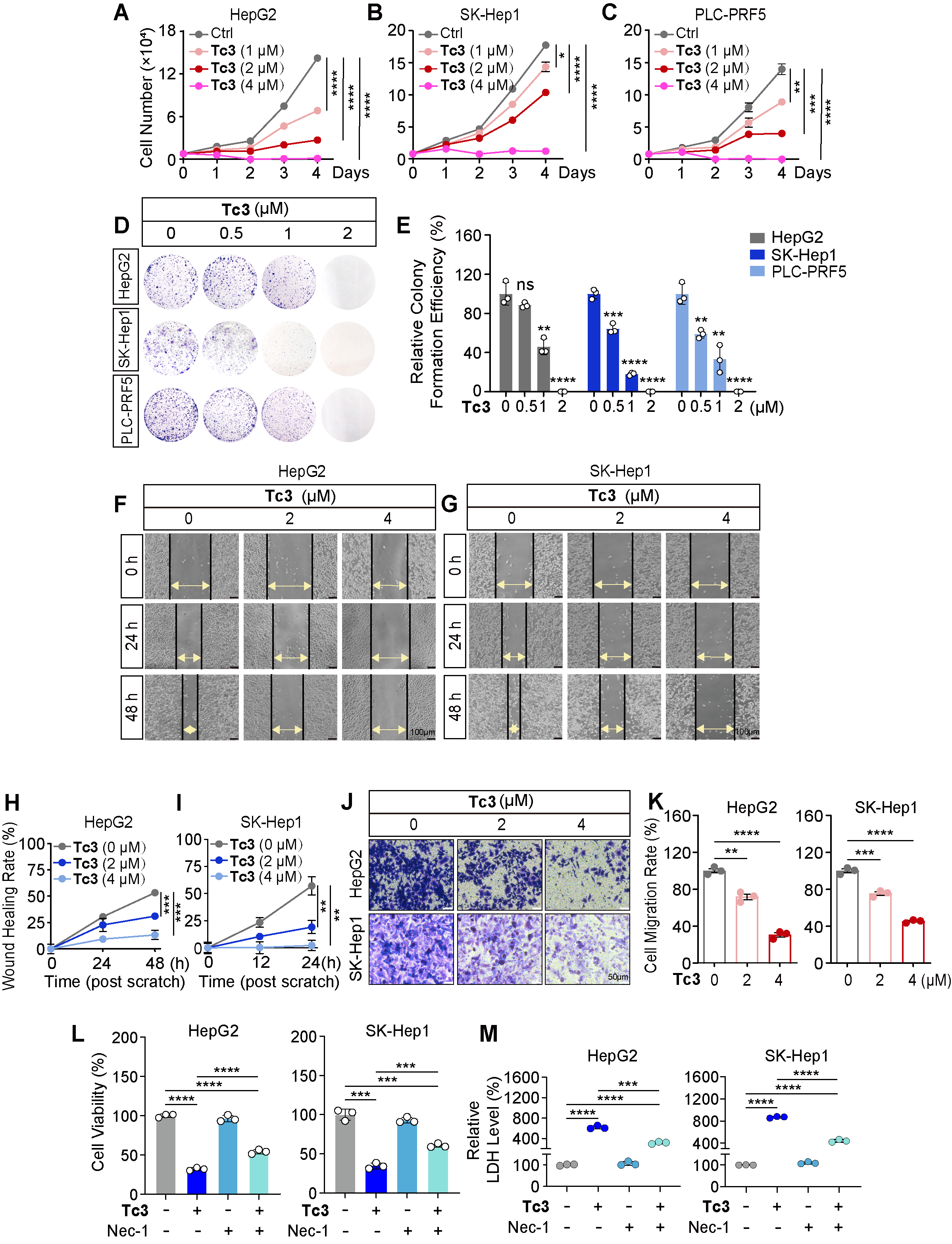


**Figure S2** **Cytotoxic effect of Tc3 on human hepatic carcinoma cells *in vitro***

1. C) Cell proliferation curves showing inhibition of HepG2 (A), SK-Hep1 (B) and PLC-PRF5 (C) cell growth by **Tc3** at a concentration gradient (0-4 μM).
2. E) Colony formation of HepG2, SK-Hep1 and PLC-PRF5 cells after treatment with **Tc3** at a concentration gradient (0-2 μM) (D), and the corresponding statistical analysis (E).

(F-I) Wound scratch assay of HepG2 (F) and SK-Hep1 (G) cells after treatment with **Tc3** at a concentration gradient (0-4 μM) and the statistical analysis of the wound healing rate (H-I). Scale bars: 100 μm.

1. K) Representative transwell images of HepG2 and SK-Hep1 cells after treatment with **Tc3** (J) and the quantitative statistics analysis (K). Scale bars: 50 μm.

(L-M) Cell viability test (L) and LDH release assay (M) of HepG2 and SK-Hep1 cells treated with **Tc3** (2 μM) alone or in combination with Necrostatin-1 (10 μM).

(Data are presented as mean ± SD of three independent biological experiments. **p* < 0.05, ***p* < 0.01, ****p* < 0.001 and *****p* < 0.0001, ns, no significant).

**Figure S3**


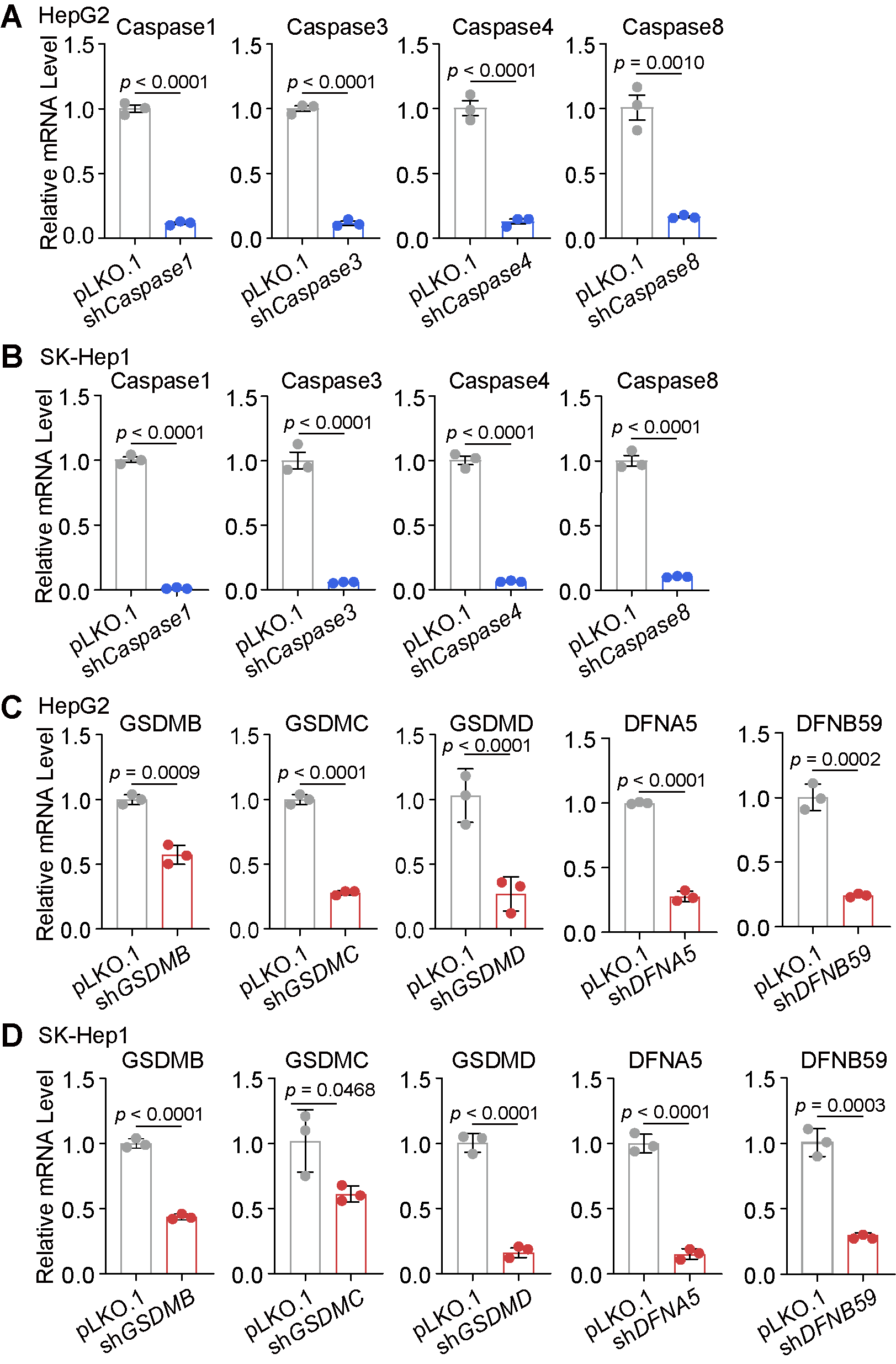


**Figure S3** **Identification of the knockdown efficacy in hepatic carcinoma cells**

(A-B) The mRNA level of *caspase1*, *caspase3*, *caspase4* and *caspase8* in the stable knockdown HepG2 (A) and SK-Hep1 (B) cells.

(C-D) The mRNA level of *GSDMB*, *GSDMC*, *GSDMD*, *DFNA5* and *DFNB59* in the stable knockdown HepG2 (C) and SK-Hep1 (D) cells.

(Data are mean ± SD of three biologically independent experiments).

**Figure S4**


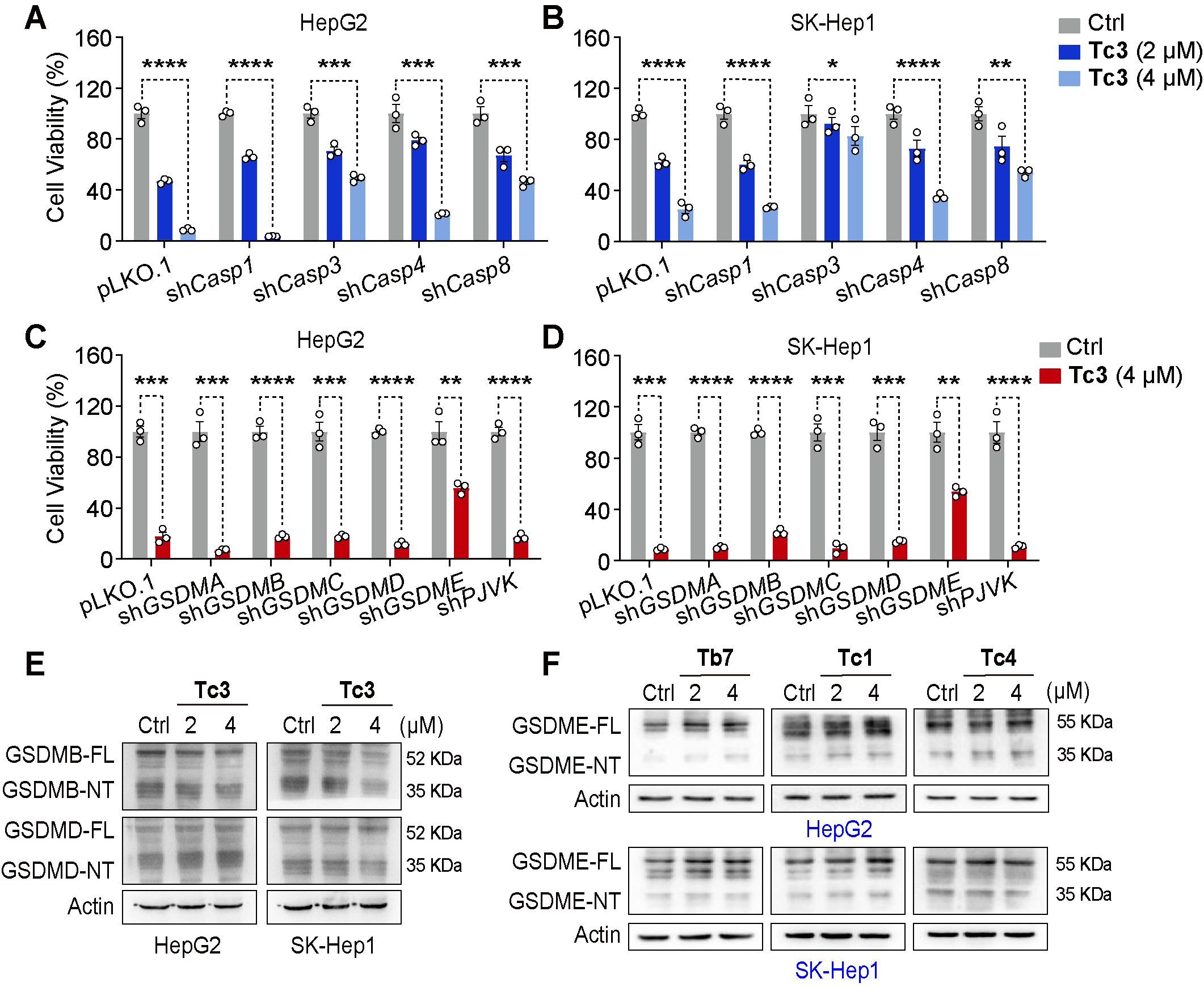


**Figure S4** **Tc3 activates the caspase8-caspase3-GSDME axis in** **hepatic carcinoma cells**

1. B) Cell viability rates of pLKO.1, sh*Caspase1*, sh*Caspase3*, sh*Caspase4* and sh*Caspase8* HepG2 (A), SK-Hep1 (B) cells treated with **Tc3**.

(C-D) Cell viability rates of pLKO.1, sh*GSDMA*, sh*GSDMB*, sh*GSDMC*, sh*GSDMD,* sh*GSDME* and sh*PJVK* HepG2 (C) and SK-Hep1 (D) cells treated with **Tc3**.

(E) Immunoblot analysis showing activation of GSDMB and GSDMD in HepG2 and SK-Hep1 cells treated with **Tc3**.

(F) Immunoblot analysis of GSDME in HepG2 and SK-Hep1 cells treated with **Tb7**, **Tc1** and **Tc4**.

(Data are presented as mean ± SD of three independent biological experiments. **p* < 0.05, ***p* < 0.01, ****p* < 0.001 and *****p* < 0.0001).

**Figure S5**


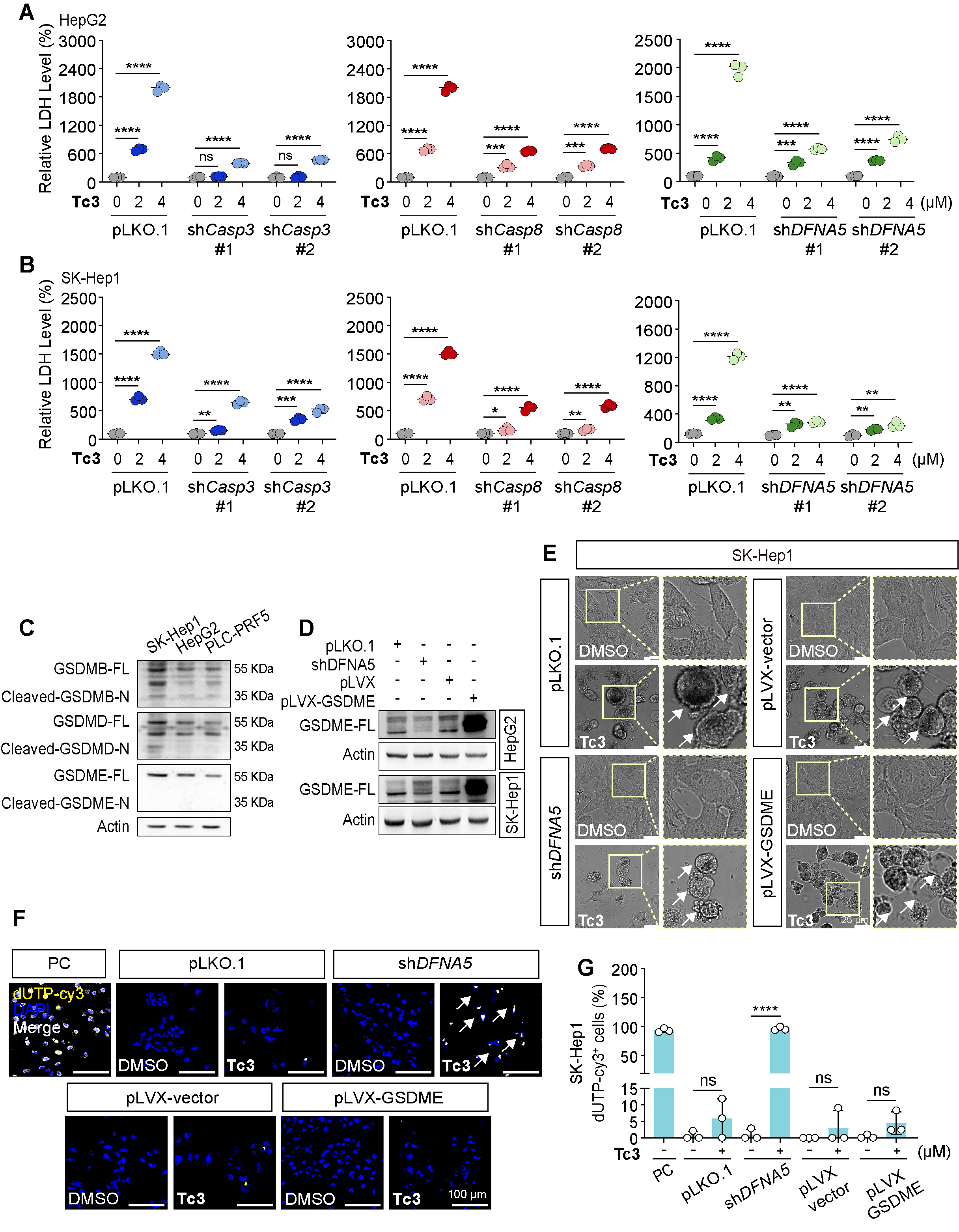


**Figure S5 The level of GSDME regulates the cytotoxic effect of Tc3 *in vitro***

1. LDH release assay of pLKO.1, sh*Caspase3,* sh*Caspase8* and sh*DFNA5* HepG2 cells after treatment with **Tc3**.
2. LDH release assay of pLKO.1, sh*Caspase3,* sh*Caspase8* and sh*DFNA5* SK-Hep1 cells after treatment with **Tc3**.
3. Immunoblotting of GSDMB, GSDMD and GSDME in HepG2, SK-Hep1 and PLC-PRF5 cells. The level of GSDME is highest in SK-Hep1 cells.
4. Immunoblotting of GSDME in pLKO.1, sh*DFNA5*, pLVX-vector and pLVX-GSDME HepG2 and SK-Hep1 cells.
5. Microscopy images of pLKO.1, sh*DFNA5*, pLVX-vector and pLVX-GSDME SK-Hep1 cells treated with **Tc3** and control medium. Scale bars: 25 μm.

(F-G) Fluorescence staining images of pLKO.1, sh*DFNA5*, pLVX-vector and pLVX-GSDME SK-Hep1 cells after treatment with **Tc3** (F). Apoptotic cells were labeled by dUTP-cy3, and cell nucleus were labeled by DAPI. Scale bars: 100 μm. The statistical analysis of dUTP-cy3 positive cells of SK-Hep1 cells (G). PC, positive control.

(Data are presented as mean ± SD of three independent biological experiments. **p* < 0.05, ***p* < 0.01, ****p* < 0.001 and *****p* < 0.0001, ns, no significant).

**Figure S6**


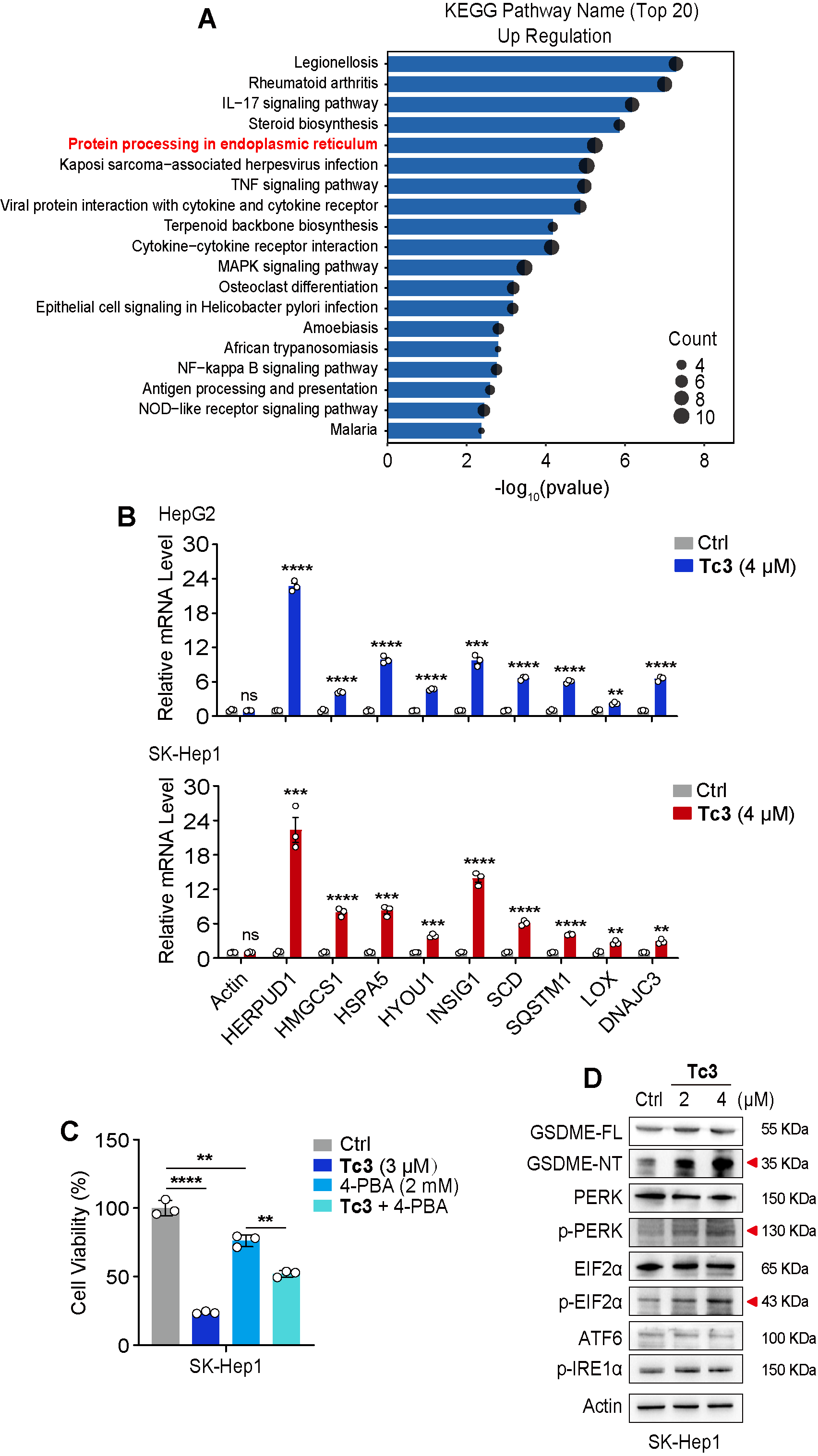


**Figure S6 Tc3 induces pyroptosis via activated ER stress in hepatic carcinoma cells**

1. KEGG pathway enrichment of up-regulated genes (fold change 2 and p adjusted< 0.05) revealed “Protein processing in endoplasmic reticulum” as one of the strongly associated biological process in the **Tc3** group.
2. The mRNA level of representative up-regulated genes in **Tc3** group in HepG2 and SK-Hep1 cells.
3. The cell viability rate of SK-Hep1 cells after treatment of **Tc3** combined with 4-PBA.
4. Immunoblotting of expression of GSDME, PERK, p-PERK, EIF2α, p-EIF2α, ATF6 and p-IRE1α in SK-Hep1 cells treated with **Tc3**.

(Data are mean ± SD of three biologically independent experiments. ***p* < 0.01, ****p* < 0.001 and *****p* < 0.0001, ns, no significant).

**Figure S7**


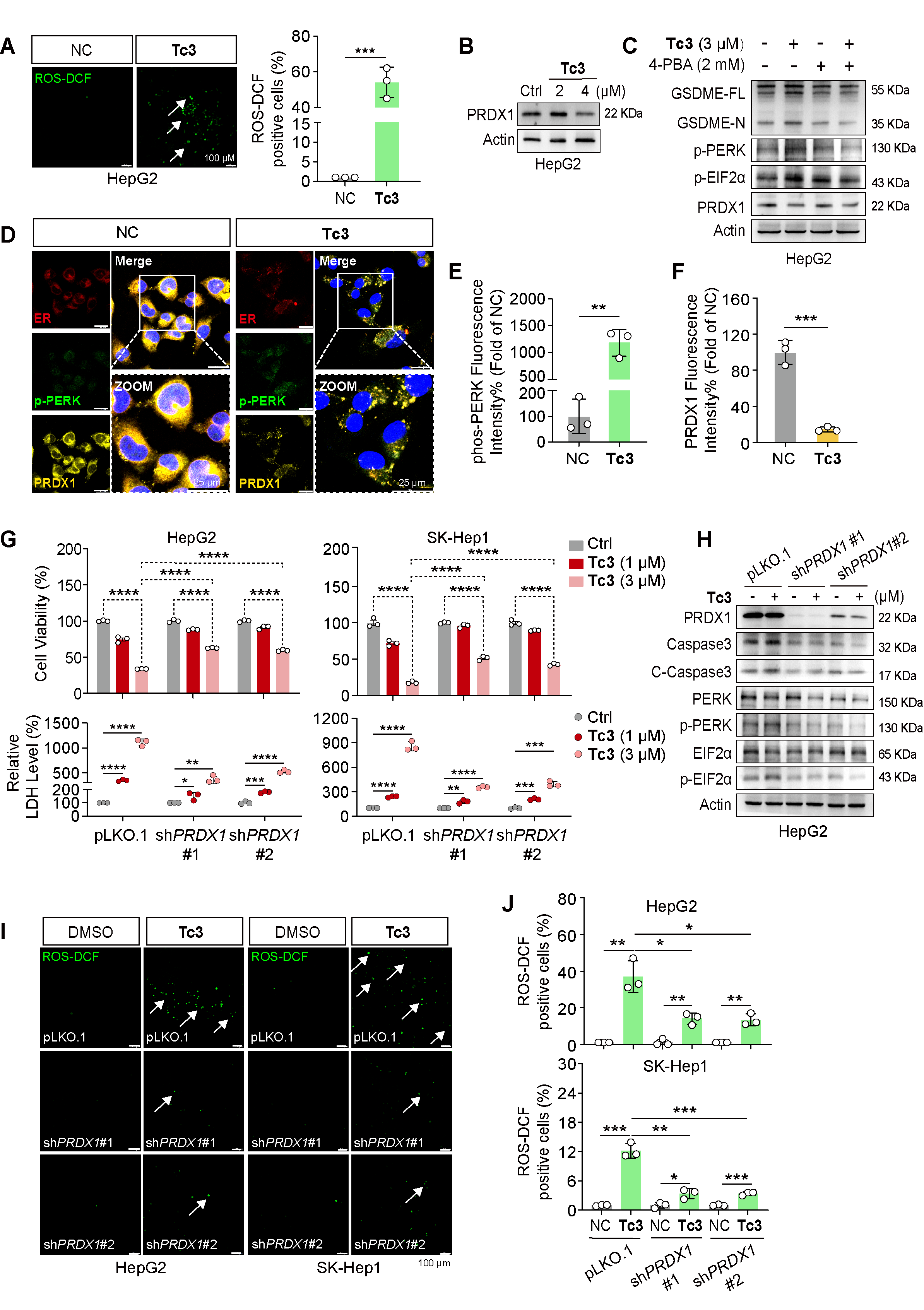


**Figure S7 Tc3 induces pyroptosis via activated ER stress in hepatic carcinoma cells**

1. The ROS detection of HepG2 cells treated with **Tc3** and the statistical analysis.
2. Immunoblotting showing inhibition of PRDX1 in HepG2 cells treated with **Tc3**.
3. Immunoblotting of expression of GSDME, p-PERK, p-EIF2α and PRDX1 in HepG2 cells treated with **Tc3** or combined with 4-PBA.
   (D-F) Representative fluorescence staining images of level and location of p-PERK and PRDX1 and ER in HepG2 cells treated with **Tc3** (D). Statistical analysis of fluorescence intensity of p-PERK (E) and PRDX1 (F).

(G) Cell viability test and LDH release assay of pLKO.1 and sh*PRDX1* HepG2 and SK-Hep1 cells treated with **Tc3**.

(H) Levels of PRDX1, caspase3, PERK, p-PERK, EIF2α, p-EIF2α in pLKO.1, sh*PRDX1* HepG2 cells treated with **Tc3**.

(I-J) The ROS detection of pLKO.1 and sh*PRDX1* HepG2 and SK-Hep1 cells treated with **Tc3** (I) and the statistical analysis (J).

(Data are mean ± SD of three biologically independent experiments. **p* < 0.05, ***p* < 0.01, ****p* < 0.001 and *****p* < 0.0001, ns, no significant).

**Figure S8**


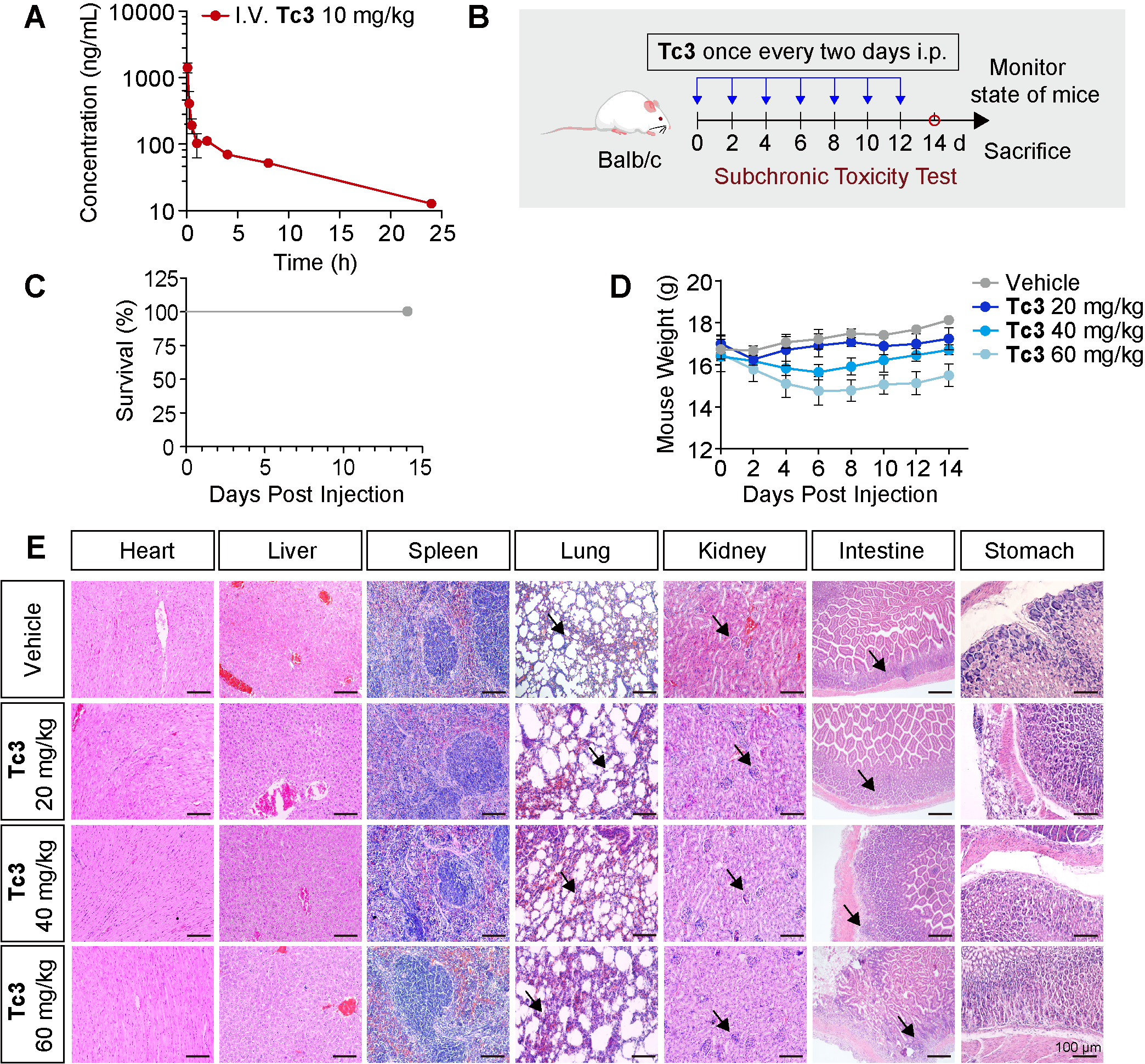


**Figure S8 The subchronic toxicity test in Balb/c mice treated with Tc3**

1. Blood concentration–time curve following vein administration of **Tc3** (10 mg/kg) in Balb/c mice.
2. Graphics depicting the subchronic toxicity process of injection of **Tc3** in Balb/c mice (n = 5).

(C-D) Mice survival (C) and weight (D) during the subchronic toxicity test.

1. Evaluation of the toxicity of **Tc3** in different organs by HE staining. Assays are tested in triplicate. Scale bars: 100 μm.

**Figure S9**


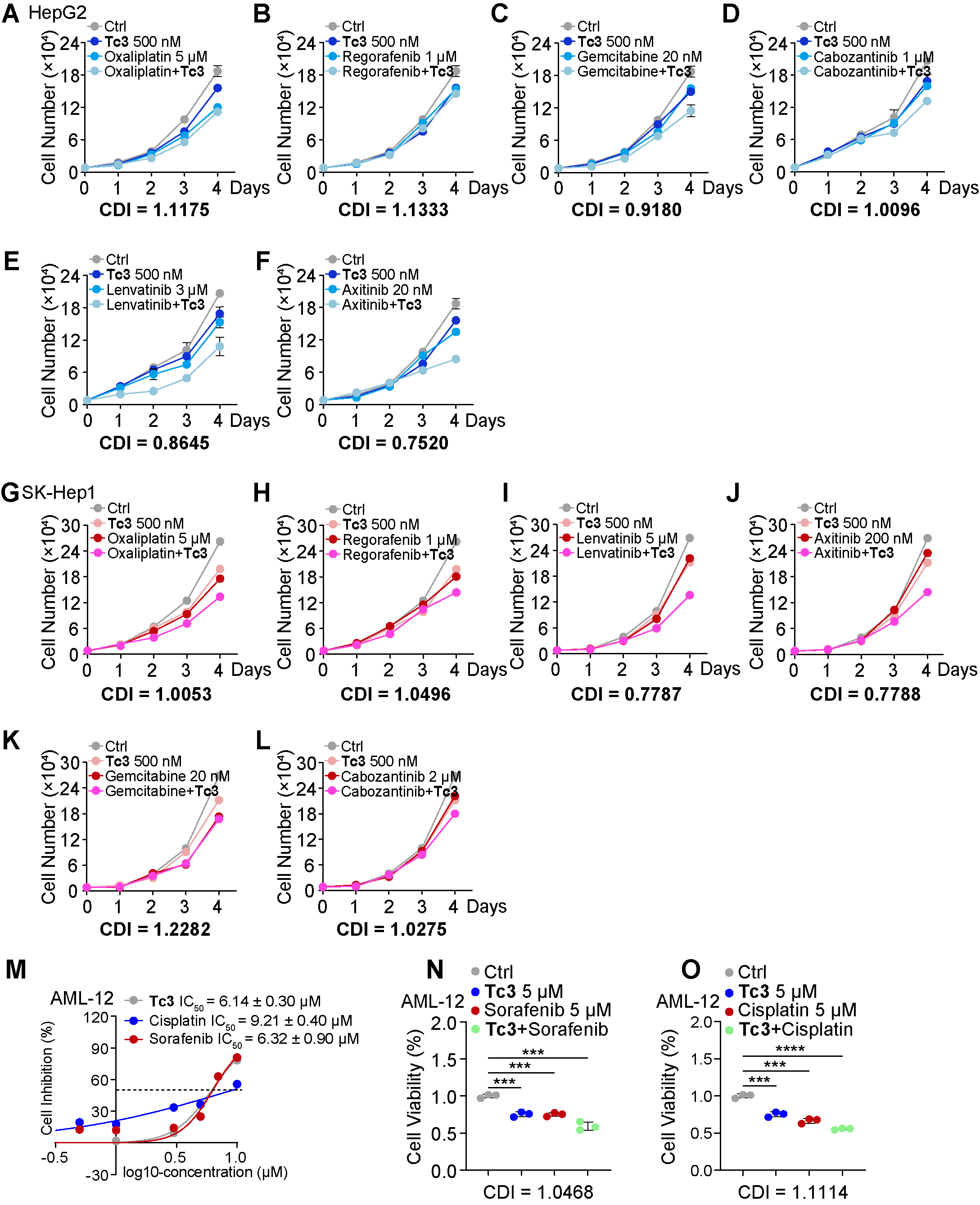


**Figure S****9 Detection of synergistic effect of Tc3 and other clinical drugs**

1. F) The combination of **Tc3** with oxaliplatin (A), regorafenib (B), gemcitabine (C), cabozantinib (D), lenvatinib (E) and axitinib (F) respectively in HepG2 cells.
2. L) The combination of **Tc3** with oxaliplatin (G), regorafenib (H), gemcitabine (I), cabozantinib (J), lenvatinib (K) and axitinib (L) respectively in SK-Hep1 cells.
3. Dose-response curves for **Tc3**, cisplatin and sorafenib inhibiting the growth of AML-12 cells.

(N-O) Combination effects of **Tc3** with cisplatin (N) and sorafenib (O) in AML-12 cells.

(Data are mean ± SD of three biologically independent experiments. ****p* < 0.001 and *****p* < 0.0001).

**Figure S10**


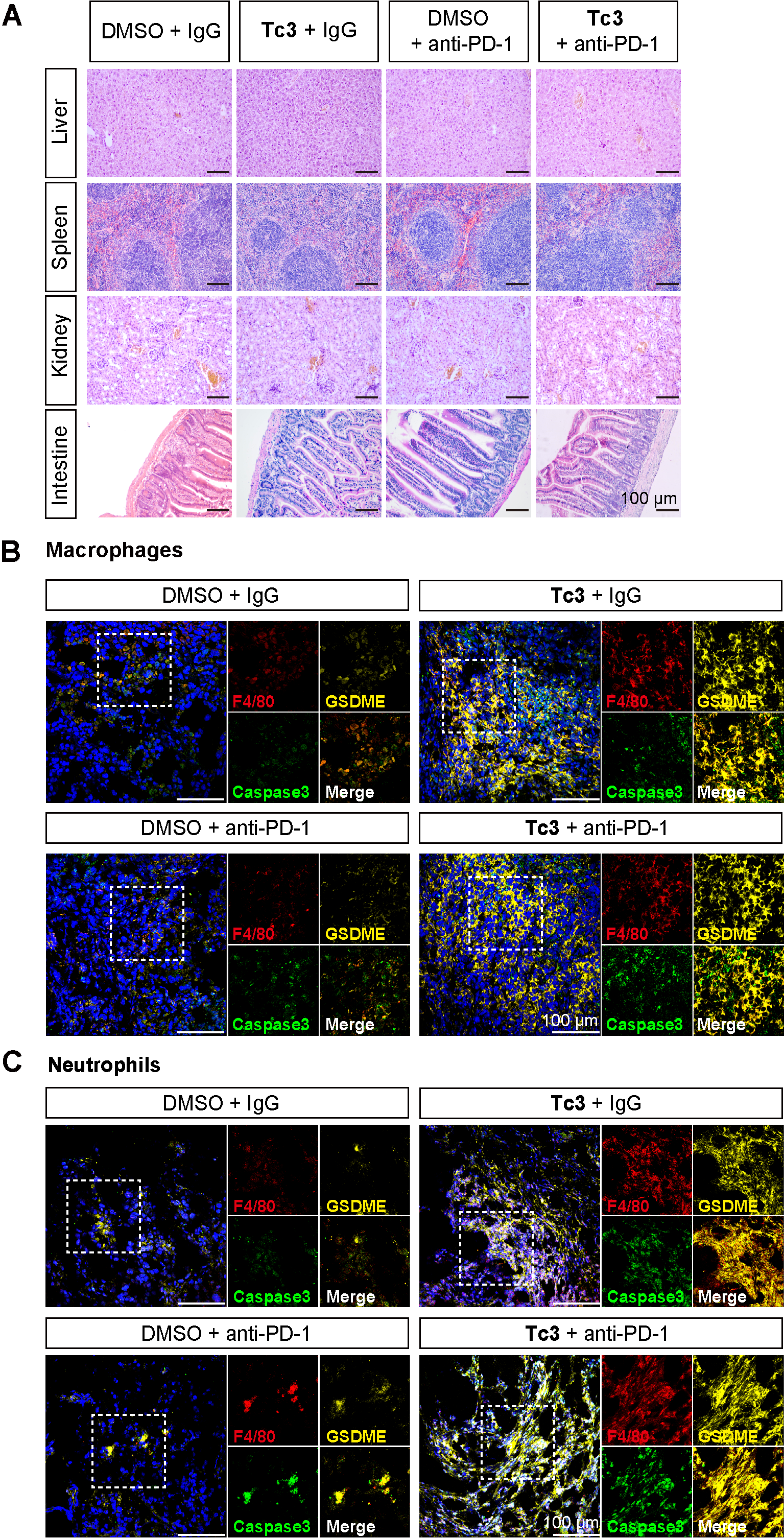


**Figure S****10** **Tc3 enhanced the infiltration of neutrophils and macrophages *in vivo***

(A) Evaluation of the toxicity of **Tc3** combined with anti-PD-1 antibody in different organs by HE staining. Scale bars: 100 μm.

(B) Representative fluorescence staining images of level and location of Ly6G, GSDME and caspase3 in tumor tissues.

(C) Representative fluorescence staining images of level and location of F4/80, GSDME and caspase3 in tumor tissues. Scale bars: 100 μm.

**Figure S11**


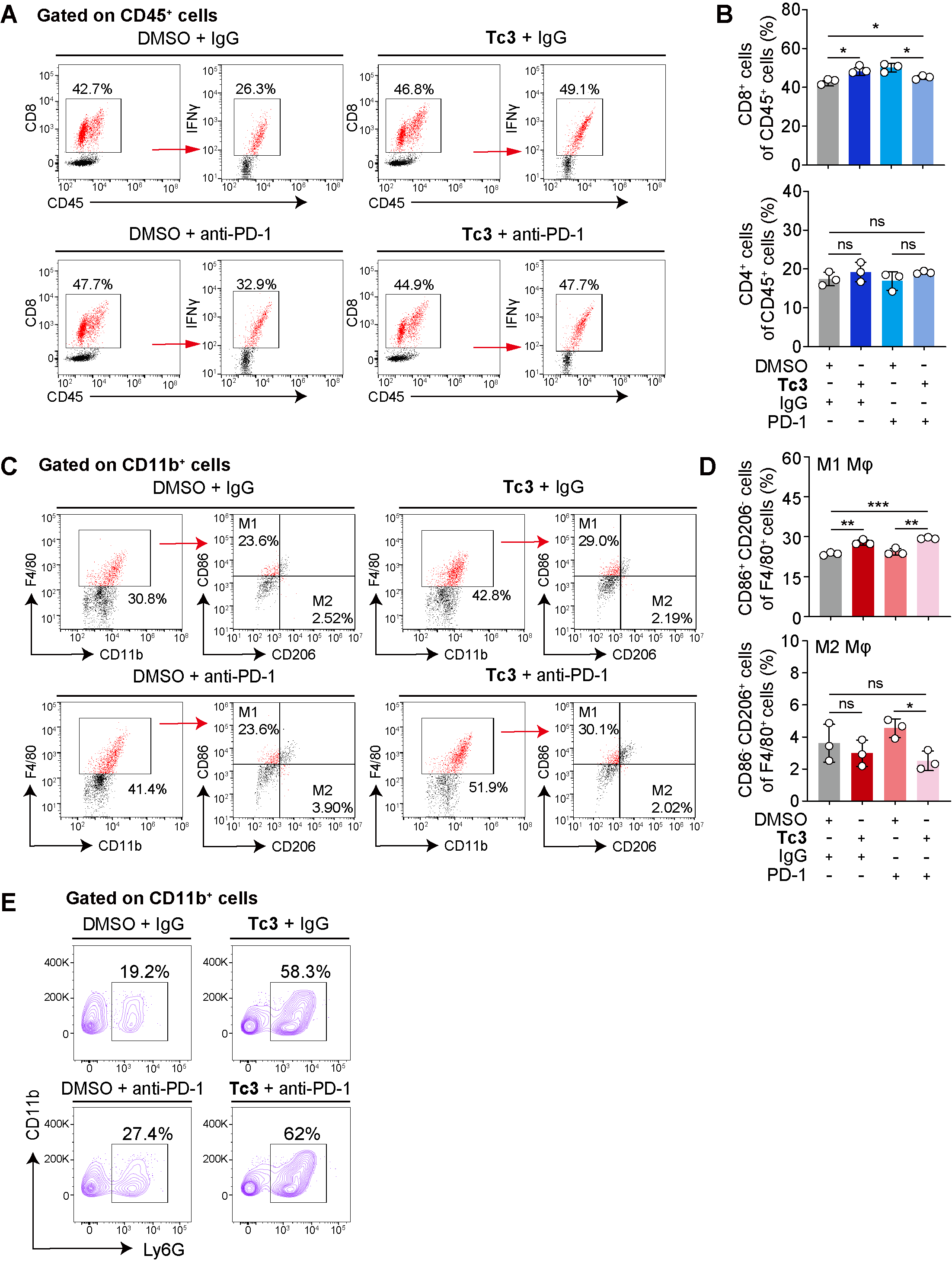


**Figure S11 Tc3** **synergizes anti-PD-1 therapy to activate the anti-tumor immunity *in vivo*.**

1. B) Flow cytometry data showing infiltration of CD8^+^ T cells and CD4^+^ T cells (A) and the statistical analysis (B).

(C-D) Flow cytometry data showing infiltration of M1 macrophages and M2 macrophages (C) and the statistical analysis (D).

(E) Flow cytometry data showing infiltration of neutrophils.

(Data are mean ± SD of three biologically independent experiments. **p* < 0.05, ***p* < 0.01 and ****p* < 0.001, ns, no significant).
